# Automatic classification of takeaway food outlet cuisine type using machine (deep) learning

**DOI:** 10.1016/j.mlwa.2021.100106

**Published:** 2021-12-15

**Authors:** Tom R.P. Bishop, Stephanie von Hinke, Bruce Hollingsworth, Amelia A. Lake, Heather Brown, Thomas Burgoine

**Affiliations:** a UKCRC Centre for Diet and Activity Research (CEDAR), MRC Epidemiology Unit, University of Cambridge School of Clinical Medicine, Box 285 Institute of Metabolic Science, Cambridge Biomedical Campus, Cambridge CB2 0QQ, UK; b School of Economics, University of Bristol, Bristol BS8 1TU, UK,; c Erasmus School of Economics, Erasmus University Rotterdam, Netherlands; d Health Research, Lancaster University, LA1 4YW, UK; e School of Health and Life Sciences, Centre for Public Health Research, Teesside University, Middlesbrough TS1 3BX, UK; f Fuse – Centre for Translational Research in Public Health, Newcastle NE1 4LP, UK; g Population Health Sciences Institute, Newcastle University, NE1 4LP, UK

**Keywords:** Takeaway (‘fast-’) food outlets, Cuisine type, Classification, Machine (deep) learning, Universal Language Model Fine-tuning (ULMFiT), Data science

## Abstract

**Background and purpose:**

Researchers have not disaggregated neighbourhood exposure to takeaway (‘fast-’) food outlets by cuisine type sold, which would otherwise permit examination of differential impacts on diet, obesity and related disease. This is partly due to the substantial resource challenge of manual classification of unclassified takeaway outlets at scale. We describe the development of a new model to automatically classify takeaway food outlets, by 10 major cuisine types, based on business name alone.

**Material and methods:**

We used machine (deep) learning, and specifically a Long Short Term Memory variant of a Recurrent Neural Network, to develop a predictive model trained on labelled outlets (n=14,145), from an online takeaway food ordering platform. We validated the accuracy of predictions on unseen labelled outlets (n=4,000) from the same source.

**Results:**

Although accuracy of prediction varied by cuisine type, overall the model (or ‘classifier’) made a correct prediction approximately three out of four times. We demonstrated the potential of the classifier to public health researchers and for surveillance to support decision-making, through using it to characterise nearly 55,000 takeaway food outlets in England by cuisine type, for the first time.

**Conclusions:**

Although imperfect, we successfully developed a model to classify takeaway food outlets, by 10 major cuisine types, from business name alone, using innovative data science methods. We have made the model available for use elsewhere by others, including in other contexts and to characterise other types of food outlets, and for further development.

## 1 Background and purpose

On average, takeaway (‘fast-’) food outlets sell energy-dense, nutrient-poor foods, which are typically served in large portions (Monsivais, & Drewnowski, 2007). Diets of regular takeaway food consumers tend to be higher in total energy than those who consume takeaway food less frequently (Adams et al., 2015), and frequent consumption of takeaway food has been associated with excess weight gain over time (Pereira et al., 2005). In the UK, only frequent use of takeaways selling hot food intended for consumption off the premises, and not use of cafes nor restaurants, was associated with obesity risk (Penney et al., 2017).

While a growing number of studies have demonstrated an association of neighbourhood exposure to unhealthy takeaway food outlets with poor diet, greater body weight and odds of obesity (Burgoine et al., 2016, Burgoine et al., 2014, Burgoine et al., 2018), the evidence base remains equivocal (Black et al., 2013, Fleischhacker et al., 2011, Wilkins et al., 2019). In some instances, this may be the result of exposure misclassification i.e. incorrect specification of a causally relevant environmental exposure (Cummins, Clary, & Shareck, 2017), which serves to mask true associations and potentially biases any observed associations towards the null (Hutcheon, Chiolero, & Hanley, 2010). Specifically, neighbourhood research studies to date have not disaggregated the broad ‘class’ of takeaway food outlet by cuisine type (Miura, Giskes, & Turrell, 2011). There are approximately 55,000 takeaway food outlets in England (Burgoine, Monsivais, & the Feat Development Team, 2017), belonging to multiple major takeaway cuisines, including chicken, kebab, and pizza shops, traditional British ‘greasy spoons’ (outlets specialising in fried foods), fish and chip shops, and those of Indian (South Asian), African, Chinese (Southeast & East Asian), and Caribbean origins (Shift, 2018). Although unhealthy overall, it is possible that neighbourhood exposure to takeaways selling some cuisines is more strongly associated with diet and health, as a result of differences in the nutritional composition and characteristics of foods sold.

A paucity of research on the impacts of exposure to takeaways of different types may be due to a lack of well-characterised takeaway food outlet data. Research studies are increasingly undertaken at scale, involving large numbers of participants. Therefore in any given study, large numbers of food outlets, to which many thousands of study participants are exposed, would be in need of classification by cuisine type to permit analysis. Although it has been historically possible in small studies (Lake, Burgoine, Greenhalgh, Stamp, & Tyrrell, 2010), manual classification of outlets by cuisine at scale is unrealistic, and characterisation by multiple researchers can result in inter-rater bias. Moreover, there may be insufficient information available on each outlet, even online, to permit accurate desk-based classification of cuisine type by a human.

To accomplish this classification task, there may be scope for the application of automated classification methods, which have been used in other areas of research. For example, machine learning, and specifically deep learning classifiers can automatically identify relevant literature from an initial broad set of review results, where manual identification might otherwise heavily burden systematic reviewers (Varghese, Agyeman-Badu, & Cawley, 2020). From a set of human-labelled records, a classifier will effectively learn the ‘language’ of how records are classified, to the extent that the classifier can be used to predict classification of records in unseen data. Although it is not known whether takeaway food outlet cuisine type can be accurately predicted from business name *alone*, elsewhere there is precedent for classifiers having been able to successfully make predictions from a similarly limited amount of data e.g. of nationality from surname only (Lee et al., 2017).

Our study was motivated by the need for detailed characterisation of takeaway food outlets by cuisine type, in order to overcome possible exposure misclassification in public health research that addresses the impacts of the neighbourhood food environment. Further, because manual classification by type would often be unfeasible, we sought to understand whether this task could be accurately accomplished and automated using data science methods, based on very limited information but that which would be commonly available to researchers. Therefore, we tested the feasibility of using innovative machine (deep) learning methods to automate prediction of takeaway food outlet cuisine type from business name alone (Section 2), and validated the accuracy of this approach (Section 3). As a case study of how this classifier could be applied to enrich existing data for the purposes of knowledge generation, we subsequently applied our predictive model to characterise nearly 55,000 takeaway food outlets in England by cuisine type, for the first time (Section 4). Section 5 contains a discussion of our results, followed by our conclusions in Section 6. We share our code so that other researchers can adapt and improve our model.

## 2 Material and methods

**Fig. 1:**
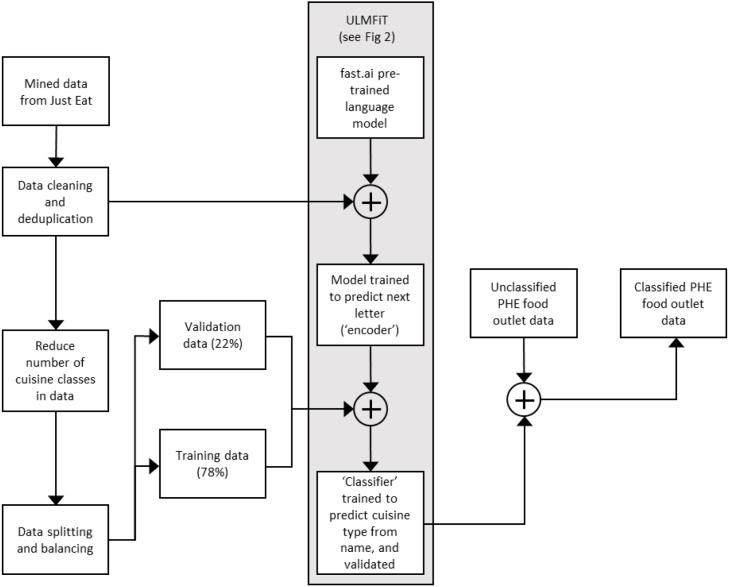
Flow chart showing key data preparation steps, leading to development of the classifier, and application to the target dataset.

Our overall approach (illustrated in Fig. 1) involved preparation and use of a training dataset, which in this case constituted a set of takeaway outlet business names with pre-annotated labels indicating cuisine type. This training data was used to build a model (a classifier) that automatically predicted takeaway outlet cuisine type in a validation dataset. The cuisine type of business names in the validation dataset are known, but they have not been used for model building. The validation dataset is used to assess the performance of the model, allowing refinements to be made and tested, and a final classifier to be developed, before application to an unclassified target dataset.

### 2.1 Data acquisition

Just Eat is the market leader for online ordering and delivery of foods prepared outside of the home in the UK. We developed training and validation datasets from labelled data on takeaway outlets mined from the Just Eat website (www.just-eat.co.uk). The use of these data for research purposes is permitted by an exemption to copyright from the Intellectual Property Office of the UK Government (Intellectual Property Office of the UK Government, 2014). We obtained data on 33,592 takeaway food outlets in November 2019.

On sign up to Just Eat, business owners are given the opportunity to assign up to three cuisine type labels to their listing (e.g. Cromwell’s Chinese Takeaway is labelled Chinese and Thai). These labels help website customers to filter the list of outlets willing to deliver food to them by cuisine type.

### 2.2 Data cleaning and pre-processing

We broadly followed the steps described by Ross (2018), to prepare business names for training and validation. Non-ASCII characters (e.g. ©, é) were removed or converted to an ASCII equivalent, and all characters were converted to lower case. Leading and trailing spaces were removed from business names. We then cleaned the data for duplicates, because this could bias our results if we had the same business names in training and validation datasets. Deduplication therefore ensures the validation dataset is entirely unseen. The Just Eat data also features outlets that are part of regional or national chains. The names of these businesses are therefore repeated in the data. Some chains such as Burger King were known *a priori*. When chains were not known, we noted that they often had the chain name followed by the location. The location was usually preceded by a hyphen or wrapped in brackets, which could be used to identify and remove them e.g. ‘Roosters Piri Piri – Stockwell’ and ‘Tops Pizza (Trumpington)’. Duplicates identified within chains were removed, leaving only one record. Finally, we removed duplicates that occurred simply as a result of common words and phrases in outlet names e.g. ‘Golden Wok’. Care had to be taken with deduplication as cuisine type labels were not necessarily the same across duplicates, even within chains. We retained the two labels that occurred most frequently across duplicates, and when a tie occurred, we retained the first two labels alphabetically.

### 2.3 Data classification

Takeaways were categorised by owners using a total of 147 cuisine labels, as shown in the middle column of Table A1. We assigned these 147 takeaway cuisine types to a 10-point takeaway cuisine classification system (as shown in the left hand column of Table A1), which describes either specific types of food sold or their region of origin, respectively: chicken, kebab, pizza, burger, multi fast food (see below), desserts, sandwich/café/bakeries, fish and chips; South Asian, Southeast & East Asian. This classification system was based on previous high street survey research that identified common cuisine types (Shift, 2018), while also accounting for the distribution of cuisine labels in Just Eat data; principally the existence of enough outlets of any given cuisine type on which to train (see 2.4 *Data splitting and balancing*). Cuisine types with too few outlets to permit training, for example those labelled as Russian (n = 7) or Tapas (n = 18), were excluded from our training dataset (i.e. not used for the purposes of classification, Table A1). The majority of business owners assign two labels to their outlet; the first label in our 10-point classification system was used as its type. For takeaway outlets with three labels, the third label assigned was always Halal. We discarded this label for the purposes of defining cuisine type.

To exploit all of the information available to us in the Just Eat dataset, we also used information contained within the business name to assist classification (as shown in the right hand column of Table A1). If an outlet had chicken, kebab, pizza or burger in the name, we prioritised this over owner assigned labels in determining cuisine type. If an outlet had more than one of chicken, kebab, pizza or burger in the name, we prioritised assignment to our multi fast food cuisine type.

Taking further priority over labels assigned by owners and information in business names, we assigned some cuisine types ourselves where an outlet belonged to a retail chain. These cuisine types were for outlets belonging to a major chain with more than 50 stores in the UK (as shown in the right hand column of Table A1), as follows: McDonald’s, Burger King (burger); KFC (chicken); Pizza hut, Papa John’s, Domino’s (pizza); Subway, Greggs (sandwich/café/bakeries). These cuisine types were assigned to ensure consistency of prediction across chains, where individual outlets might be classified differently, for example when belonging to a franchise, or where they were notably absent from our training dataset through not being present on Just Eat *at this time* (i.e. McDonalds, Domino’s, Greggs). Examples of classification rules applied to Just Eat data are shown in Table 1.

After the cleaning and classification process, we retained 18,145 food outlet records in our dataset for the purposes of training and validation.

**Table 1:** Examples of classification rules applied to Just Eat data.

Just Eat Data			Cuisine type	Rationale
Name	Label 1	Label 2		
Tom’s House	Burger	Healthy	Burger	Burger^a^ as a label
Tom’s Grill	Chicken	Burger	Chicken	Chicken^a^ as label 1; label 1 takes precedent over label 2
Tom’s Kebab House	South Asian	Pizza	Kebab	Kebab^a^ in name; name takes precedent over both labels
Tom’s Pizza and Chicken Shack	Burger	Kebab	Multi fast food	Pizza^a^ and chicken^a^ in name; name takes precedent over both labels
McDonald’s	No label	No label	Burger	Chain outlet not present in training data; label assigned

a Outlet cuisine type present in 10-point classification system.

### 2.4 Data splitting and balancing

We used a random sample of 400 business names per cuisine type for the purposes of validation, which left sufficient records for training even for the least frequently represented cuisine type (which had 822 records in total i.e. 422 for training and 400 for validation). However, some cuisine types contained many more outlets, with the largest having 4,439 (4,039 for training and 400 for validation). If the training was completed without further adjustment, the model would have performed well in predicting cuisine types with more example names in the training data, and less well on cuisine types with fewer example names. To ensure equal representation of all cuisine types in a balanced training dataset, we randomly resampled with replacement business names until all cuisine types contained 4,000 examples. Overall, the data contained 14,145 unique outlets for training and 4,000 outlets for validation.

### 2.5 Machine learning

We developed our model (a classifier) using deep learning, which is a variant of machine learning particularly suited to applications with complex input data such as images or text. Deep learning networks have many parameters, which are established via a process of trial and error where optimised settings are learned by examining pre-labelled data. Image processing uses standard feedforward neural networks where a single image is used to make an inference. When applied to text, a variant of a neural network is required that can process sequential data. A human infers understanding of a word in a sentence by looking at the previous words and the context they provide, rather than starting from scratch with each word. Therefore we required a Long Short Term Memory (LSTM) variant of a Recurrent Neural Network (RNN), which is capable of holding an internal state and therefore able to process inputs from extended sequences of data.

**Fig. 2:**
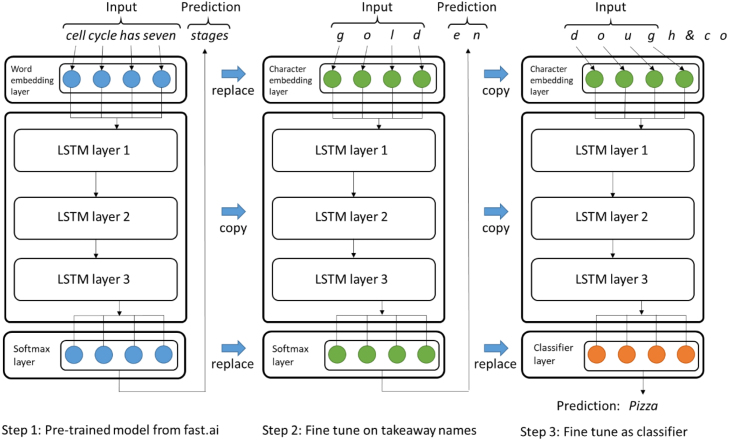
Illustration of our application of Universal Language Model Fine-tuning (ULMFiT).

Adapted from Howard, and Ruder (2018).

We broadly followed an established approach for Universal Language Model Fine-tuning (ULMFiT), which consists of refining a language model through transfer and semi-supervised learning (Howard & Ruder, 2018), and subsequent development of a character based model (Ross, 2018). These three steps are shown in Fig. 2, and have been described in detail previously (Faltl et al., 2019, Howard and Ruder, 2018). Briefly, for step 1, we began with transfer learning, which is a process whereby a language model (LM) previously trained on one dataset is fine-tuned for use on another, thus reducing the amount of new training required. We used the fast.ai platform version 1 (https://www.fast.ai/), which provides an LSTM language model that has previously been trained on a general text corpus for the task of predicting the next word in a series of words, after reading all the words before. For step 2, we took this model and trained it (a semi-supervised process) on the entire set of business names, but with the task of predicting the next character rather than the next word. Characters were used instead of words for two reasons. Firstly, business names are short, often containing only a few words, hence the task of accurately predicting the next word would be challenging. Secondly, the total vocabulary of the business names contains too few examples of each word to use a word-based model. The character based model required a bespoke tokenizer (a function to convert words to individual characters), as used by Ross (2018). For step 3, this model was modified for the task of classifying cuisine type from letters in the business name and fine-tuned on this task i.e. this is our ‘classifier’. Fine-tuning was halted when no further improvement was seen in validation accuracy (Table A2).

Hyperparameters are parameters that determine the learning process and the structure of a model. Unlike model parameters, these cannot be ‘learned’ during training. Typically, hyperparameters are set using best practice, rule of thumb or trial and error. We started with the hyperparameter values suggested by Howard and Ruder and refined these based on trial and error (Howard & Ruder, 2018).

**Fig. 3:**
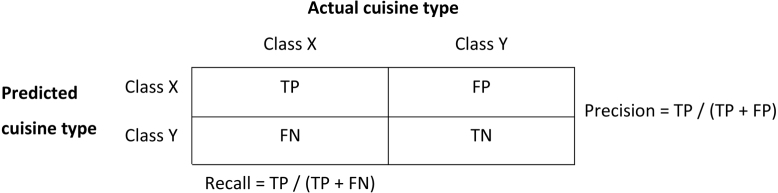
Calculation of recall and precision.

### 2.6 Statistical analysis

We tested the accuracy of our classifier on a validation dataset of 4,000 labelled outlets from Just Eat (400 outlets for each cuisine in the 10-point classification system), which we reserved for the purposes of validation. As aforementioned, none of the records in this validation data were present in the training data (i.e. the classifier had not ‘seen’ any of these takeaway outlet names before). We calculated recall (also known as sensitivity) and precision (also known as the positive predictive value (PPV)) (Lebel et al., 2017), both overall and by cuisine type (Fig. 3).

Recall measures the proportion of outlets of any given cuisine type that were correctly predicted (i.e. true positives / (true positive + false negatives)). We applied published cut-offs for describing sensitivity: <20% Very poor; 21%–30% Poor; 31%–50% Fair; 51%–70% Moderate; 71%–90% Good; >90% Excellent (Paquet, Daniel, Kestens, Léger, & Gauvin, 2008). Precision measures the proportion of predictions for any given cuisine type that were correct (i.e. true positives / (true positives + false positives)). It is possible for a model to have excellent recall but low precision, and vice versa. For example, a model might predict all chicken outlets correctly (excellent recall), but might achieve this by predicting all types of outlets as chicken outlets (low precision). We calculated overall recall and precision as the mean of recall and precision values, respectively, across all cuisine types.

We used confusion matrices to explore specific instances of misclassification. A confusion matrix compares actual to predicted classifications by cuisine type, with rows representing predicted values and columns representing actual values. Statistical analyses were conducted in Python 3.7.2.

### 2.7 Sensitivity analyses

We tested two other models as sensitivity analyses. We tested a six-point classification system, combining all outlets classified as chicken *or* pizza *or* kebab *or* burger into one cuisine type, alongside desserts, fish and chip shops, South Asian, and Southeast & East Asian outlets, and sandwich/café/bakeries. We did this to evaluate the performance of a model with fewer, broader cuisine classifications.

We also tested the performance of a ‘naïve’ classifier, manually derived from a list of words that were commonly used to describe each cuisine type. For example, we observed that the word ‘wok’ is common to Southeast and East Asian outlets, and ‘ocean’ is common to fish and chip shops. Common words such as ‘and’ and ‘takeaway’ were removed as these were common to all cuisine types. Words were given a score based on how frequently they occurred for any given cuisine type, and it was possible for a word to appear in more than one cuisine type. For each name in the validation dataset, the words it contained were used to generate a score, and the cuisine type with the highest score was used to assign the predicted label. The purpose of this model was to evaluate the added benefit of building a classifier using machine learning in this context.

## 3 Results

### 3.1 Classifier accuracy

Overall, our model had a cuisine type classification recall of 72% (“good”), with 72% precision (Table 2). This is to say, 72% of outlets had their cuisine type correctly predicted, and out of all predictions made by the model, 72% of those were correct. Prediction accuracy varied by cuisine type, and was highest for multi fast food (93% and 99% for recall and precision, respectively). In other words, out of all multi fast food outlets, 93% were predicted correctly as multi fast food, and out of all outlets predicted as multi fast food, 99% of those were actually multi fast food. Six out of 10 cuisine types were predicted with >71% (“good”) sensitivity. Recall for South Asian, Southeast and East Asian and multi fast food outlets were all >80% (near “excellent”). Eight out of 10 cuisine types were predicted with ≥65% precision.

**Fig. 4:**
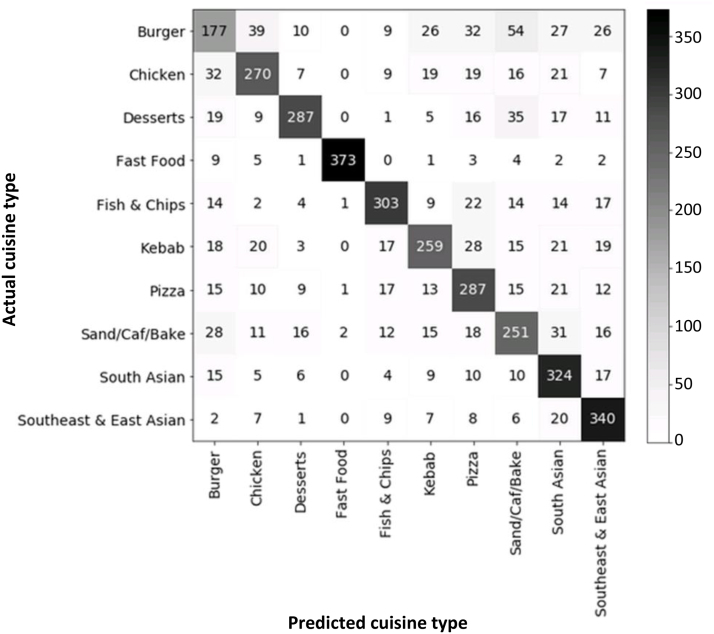
Confusion matrix, showing specific instances of misclassification. Rows total to 400 outlets.

Burger outlets had both lowest recall and precision (44% and 54%, respectively), resulting from the correct classification of only 177 of 400 burger outlets in testing, and the additional prediction of 152 outlets incorrectly as burger outlets, with the highest number of these being chicken shops (Fig. 4). Burger outlets were most often miscategorised as sandwich/café/bakeries (14% of predictions), pizza outlets (8%) or chicken shops (9%).

**Table 2:** Recall and precision results for the 10-point classifier, overall and by cuisine type.

Cuisine type	Recall, %	Precision, %
Burger	44	54
Sandwich/café/bakery	63	60
Chicken	68	71
Desserts	72	83
Kebab	65	71
Pizza	72	65
Fish and chips	76	80
South Asian	81	65
Southeast & East Asian	85	73
Multi fast food	93	99
**Overall**	**72**	**72**

Results of sensitivity analyses are shown in appendices. The naïve classifier performed relatively less well than the machine learning classifier (Table A3 and Fig A1), with 60% “moderate” (vs. 72% “good”) recall and 62% (vs. 72%) precision overall. The model was inferior in its recall across all cuisine types and inferior in its precision across all cuisine types except for burger (61% vs. 54%), pizza (66% vs. 65%) and South Asian (75% vs. 65%). The results of a machine learning model predicting a six-point classification system are shown in Table A4 and Fig A2. Compared to our 10-point model, overall recall and precision were improved (77% vs. 72% (both “good”) and 79% vs. 72%, respectively), alongside improvements in the majority of cuisine types according to both metrics. However, precision for multi fast food (56%) was markedly decreased vs. multi fast food or its constituent outlet types (except burger outlets) from the 10-point classifier, reflecting a tendency for multi fast food classification to be over-predicted, in particular as sandwich/café/bakeries.

## 4 Case study: application of the classifier to takeaway food outlet data for England

### 4.1 Background and methods

We applied our 10-point classifier to takeaway food outlet data for England, obtained from the Food Standards Agency (FSA) (Food Standards Agency, 2020b). These data, and their spatial accuracy and completeness, have been described in detail elsewhere (Kirkman et al., 2020). We wrote a Python script to collect data on 530,024 food outlets of all types in England from the FSA API in September 2019 (Food Standards Agency, 2020a). From these data we identified 54,237 takeaways using a method developed by Public Health England (PHE), described previously (Public Health England, 2018). Our aim was to provide a high-level description of the takeaway sector by cuisine type, across England overall and by lower-tier local authorities (LAs), for the first time. LAs represent the lowest level of government in England, with administrative responsibilities including appraisal of planning applications for new takeaway food outlets and hygiene inspections for all premises serving food to the public (Keeble et al., 2019). We present descriptive statistics for counts of outlets by cuisine type in England, and median counts within LAs. We then use mid-2019 population estimates from the Office for National Statistics (Office for National Statistics, 2019), to calculate counts of outlets per 100,000 resident population per LA, overall and by cuisine type. These adjusted rates were grouped into quintiles (Q5 = most outlets) and mapped within LA boundaries using a geographic information system (ArcGIS 10.5, ESRI).

### 4.2 Results

We found that Southeast & East Asian takeaway food outlets constituted the largest single takeaway food outlet cuisine type in England, with 10,254 outlets (18.9% of all takeaways), followed by pizza (16.1%) and fish and chip shops (15.4%), as shown in Table 3. Across 317 LAs, the overall takeaway outlet LA median (IQR) was 117 (80–221) outlets. The median (IQR) number of Southeast & East Asian takeaways per LA (24 (16–41)) was highest out of all cuisine types, and the highest count of any cuisine in a single LA was pizza (n = 197).

**Fig. 5:**
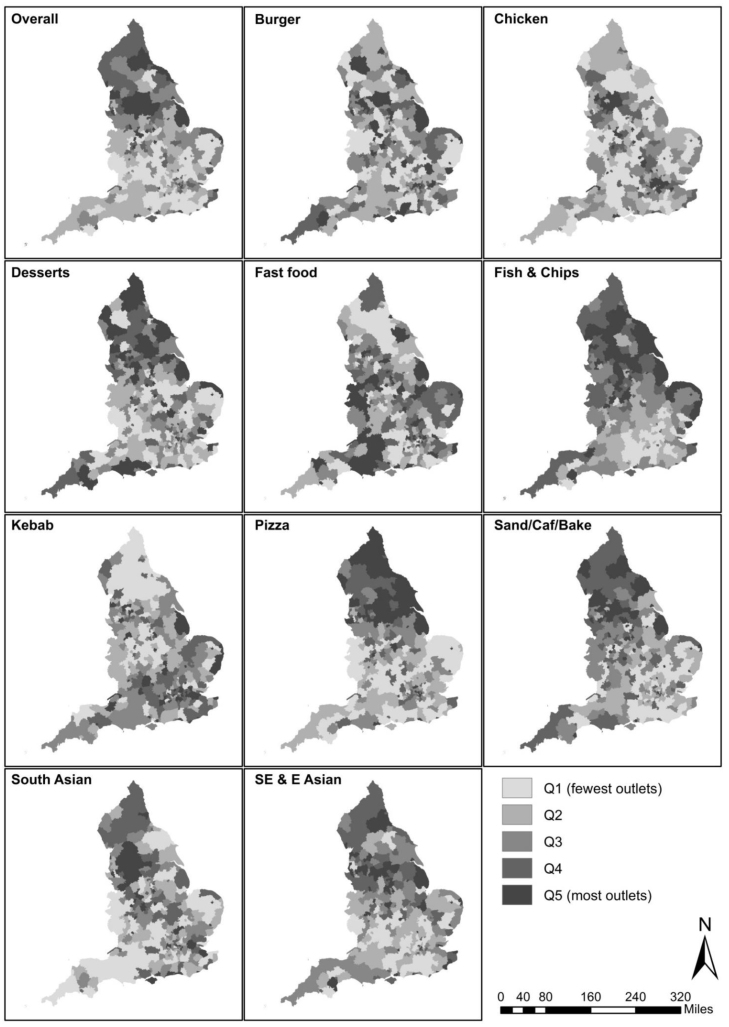
Number of takeaway food outlets per local authority per 100,000 population (quintiles (Q)), overall and by cuisine type. Source: Office for National Statistics licensed under the Open Government Licence v.3.0. Contains OS data ©Crown copyright and database right 2020.

There was variation in the geographic distribution of all takeaway food outlets per 100,000 population, and deviations from this patterning by cuisine type (Fig. 5, with large, high-resolution maps presented in Figs A3-12, and summary data for all LAs presented in Table A5). Broadly, clusters of South Asian takeaways were observed in the Northwest, across e.g. Tameside, Oldham and Blackburn with Darwen councils, and concentrated in the West Midlands (East Staffordshire and North Warwickshire), North of London (North Hertfordshire and Stevenage) and East of London (Brentwood, Basildon, Thurrock and Havering), and in the North East (South Tyneside, Gateshead and Sunderland). South Asian takeaways were relatively less common in Greater London; similarly for Southeast and East Asian takeaways, with the exception of three LAs (Tower Hamlets, Camden and Southwark). Southeast and East Asian takeaways were more concentrated in LAs in West Yorkshire (Doncaster, Wakefield, Barnsley, Sheffield), and along a corridor extending West from High Peak, through Tameside, Salford, Wigan, and St Helens to Liverpool. While also available inland (although relatively less common in Greater London), fish and chip shops were observed in high numbers along the East coast of England, e.g. in LAs such as Great Yarmouth and Scarborough.

**Table 3:** Descriptive statistics, overall and by cuisine type, for England overall and across local authorities in England (n = 317).

Cuisine type	England	Local authority	
	Outlets, n (%)	Outlets, median (IQR)	Outlets, min–max
Burger	4,323 (8.0)	10 (6–18)	0–79
Chicken	3,836 (7.1)	7 (3–15)	0–98
Desserts	1,036 (1.9)	2 (1–4)	0–32
Fast food	1,027 (1.9)	2 (1–4)	0–18
Fish and chips	8,340 (15.4)	20 (13–31)	3–130
Kebab	3,335 (6.1)	7 (4–14)	0–56
Pizza	8,728 (16.1)	18 (12–35)	0–197
Sandwich/café/bakery	6,889 (12.7)	16 (9–26)	0–120
South Asian	6,469 (11.9)	14 (9–26)	0–112
Southeast & East Asian	10,254 (18.9)	24 (16–41)	0–177
**Overall**	**54,237 (100.0)**	**117 (80–221)**	**3–919**

**Fig. 6:**
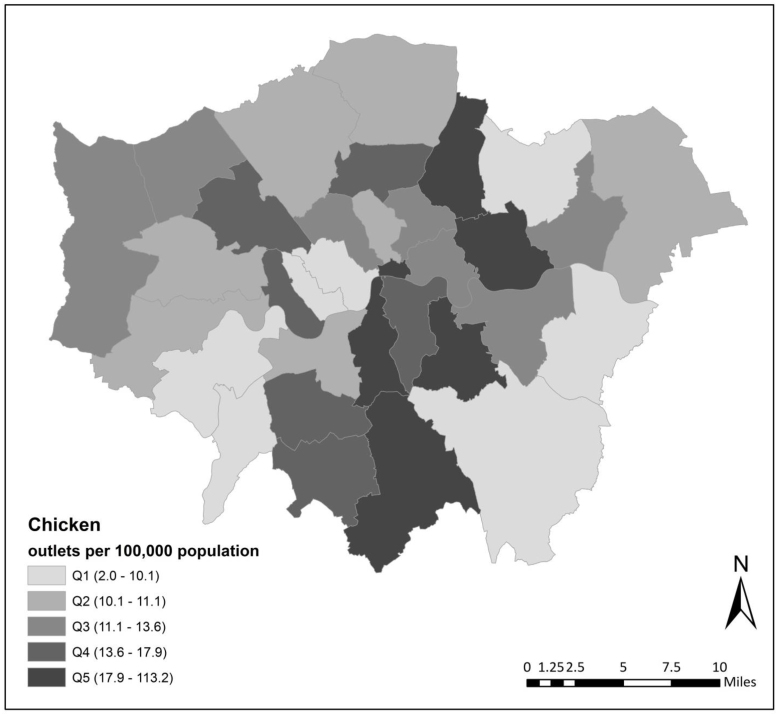
Number of chicken takeaway food outlets per local authority in Greater London per 100,000 population (quintiles (Q) relative to 33 Greater London LAs). Source: Office for National Statistics licensed under the Open Government Licence v.3.0. Contains OS data ©Crown copyright and database right 2020.

LAs with the most chicken shops were typically observed in Northwest England, in particular in the areas between Bradford, Manchester and Blackburn with Darwen councils, and especially so in LAs in Greater London. Here, 28 of 33 London councils were among the top fifth of LAs in England with respect to number of chicken shops. Relative to other local authorities *in Greater London* (Fig. 6), the City of London, Waltham Forest, Newham, Lewisham, Lambeth, and Croydon, were LAs with the highest concentrations of these outlets.

Compared to chicken shops, other types of takeaways per 100,000 population such as pizza, kebab and burger outlets were less concentrated in Greater London (Fig. 5). These outlets showed a more distributed spatial patterning across the country. To a large extent this was also true for dessert outlets, and sandwich/café/bakeries.

In addition to this high level description, we have made this classified data available publicly on GitHub (https://github.com/tombisho/takeaways). Each record in the classified data is annotated with an estimate of prediction accuracy i.e. how confidently the model made any given cuisine classification.

## 5 Discussion

In this study, we tested the feasibility of using machine learning methods to automatically predict takeaway food outlet cuisine type based on business name alone. Using labelled training data from an online takeaway food delivery service and a 10-point cuisine type classification system, we developed a model that predicted cuisine type correctly approximately three out of four times. Six out of 10 cuisine types were predicted with greater than 71% (“good”) recall, and eight types with greater than 65% precision. Prediction accuracies for South Asian, Southeast and East Asian, and multi fast food cuisines in particular, were high. Burger outlets had both lowest recall (44%) and lowest precision (54%). Low recall resulted from burger outlets being most commonly miscategorised as sandwich/café/bakeries, pizza outlets or chicken shops, and low precision resulted from the frequent prediction of chicken shops, in particular, as burger outlets. The model performed better than a naïve classification approach based only on key words, which justifies the application of machine learning in this context.

Typically, in any similar application of machine learning, there is a trade-off between classification accuracy and the amount of data available with which to make predictions. With no further human input, the optimal model is able to classify accurately using only routinely available information. It was not known whether business name alone would permit the level of discrimination by takeaway cuisine type that would be desired for data to be subsequently useful. However, we were able to build a model that accurately predicted 10 cuisine types using only this information. This model performed only marginally less well than a classifier that predicted fewer (six) cuisine types. Use of machine learning itself was justified through the additional accuracy offered when compared to our naïve classifier, which primarily used words commonly associated with cuisine types to make classifications. As well being more accurate, the machine learning model was also less labour-intensive to develop as it did not require hand-crafting of rules to manage the classification process. Moreover, the final machine learning model could be adapted to categorise other types of food outlets including restaurants, and tailored to classify takeaway food outlets in other countries. We have made the model publicly available to allow other researchers to make improvements (https://github.com/tombisho/takeaways), as well as to modify it for their own purposes (see *Availability of data and materials*).

It is possible that neighbourhood exposure to takeaway outlets selling particular cuisines is more strongly associated with diet and health outcomes. While takeaways generally sell large portions in excess of UK recommended daily allowances (Jaworowska et al., 2014, Robinson et al., 2018), studies have shown highly variable nutritional profiles for ‘indicator’ dishes (that broadly represent a cuisine) from different types of takeaway. In one city where 489 takeaway meals were analysed from across 274 independent takeaways (Jaworowska et al., 2014), energy per portion was greatest across indicator dishes from pizza outlets (mean 1,820 kcal), followed by South Asian (1,391 kcal), Southeast & East Asian outlets (1,161 kcal) and kebab shops (1,125 kcal). Meals from South Asian and pizza takeaways have been shown to contain on average 70–75 g of total fats and 13–14 g of total sugars, as compared to 37 g (total fats) and 9 g (total sugars) in meals from Southeast and East Asian takeaways (Jaworowska et al., 2014). While it was also observed that kebab shops sell meals that are comparatively low in total sugar content, these meals tend to be higher on average in trans-fatty acids (Jaworowska et al., 2014), consumption of which has been linked to cardiovascular disease incidence (de Souza et al., 2015). Energy density and nutritional composition notwithstanding, the regular consumption of red and processed meats, which are more common to some types of takeaway food outlets, has been linked to greater cardio-metabolic risk such as incidence of type 2 diabetes, coronary heart disease and stroke, and certain types of cancer (Bouvard et al., 2015, Micha et al., 2010, Micha et al., 2011).

Aside from nutritional composition, differences in the *characteristics* of food served by cuisine (e.g. packaging, preparation time, cost), may also influence use, and use among specific consumer groups, further suggesting the possibility of inequitable impacts on diet and health. For example, chicken, burger and kebab shops all sell food that is prepared and served quickly, designed to be eaten on the move, and typically available throughout long store opening hours (Thompson, Ponsford, Lewis, & Cummins, 2018). Therefore, they may be used more frequently than for example South Asian takeaways, thus potentially contributing more influentially to total dietary intake. Elsewhere, the relatively low cost of meals served in chicken shops might exaggerate their appeal to some price-sensitive groups (Bagwell, 2011), and thus their potential impacts. In one study of a chicken shop in East London, the average consumer spend was just £2.21 (Shift, 2013). When combined with targeted discounts (Bagwell, 2011), this may explain why 30% of all chicken shop visitors in this same study were less than 12 years of age (Shift, 2013).

As a case study example of the classifier’s application for the purpose of knowledge generation, we used our model to provide a high level description of the landscape of takeaway food outlets by cuisine type in England for the first time. To our knowledge, previous research has only described (less) disaggregated takeaway outlet data across a single ward in one English city (Blow, Gregg, Davies, & Patel, 2019). We applied our classifier to FSA data, which have significant research potential, owing to both its contents (e.g. business name, address, coordinates, hygiene rating) and its attributes (e.g. national coverage, completeness, real-time updates, no restrictions on reuse, zero cost) (Kirkman et al., 2020). Automated classification of takeaway food outlet records in this database by cuisine type only serves to enhance its utility. Although only a demonstration of our classifier’s potential, we observed that Southeast and East Asian cuisine constituted the largest single takeaway cuisine type in England, followed by pizza and fish and chip shops. Accounting for population, regional clusters of Southeast and East Asian, South Asian, chicken, and fish and chip shops, in particular, were observed. While the prevalence of chicken shops in Greater London has been observed in previous regional research (Bagwell, 2011, Shift, 2013), these new data have enabled the first observation of the extent of this clustering in a national context. Outside of research, cuisine-classified FSA data also have potential surveillance and decision-making applications. The National Planning Policy Framework, for example, requires local risk factors be taken into account alongside scientific research evidence when developing local authority planning policies (Ministry of Housing, Communities & Local Government, 2018). However there are no up-to-date food environment data with detailed characterisation by cuisine type available to local authorities, which could be used to assist their decision making in pursuit of improved public health.

Our study is not without limitations. We developed a food outlet cuisine type training dataset and a validation dataset for subsequent testing, based primarily on cuisine labels assigned by owners for the purposes of listing their businesses on an online delivery platform. We treated this as a ‘gold standard’, as our hypothesis was that owners know their businesses best, and would be well placed to accurately summarise what type of food was being sold. However, there may be commercial or historic reasons why these descriptions were not made accurately, for example to increase the number of searches that their business is returned in on Just Eat, or due to diversification of their product portfolio since site listing. With the resources available, we were not able to manually classify the 18,145 outlets available to us for training and validation. Future research might consider the use of data from business websites and/or outlet menus to more accurately classify food outlets prior to model training and testing. We also assigned only one cuisine type per outlet, to streamline model training. We presumed owners would label their business with the cuisine most representative of the food sold within their outlet first, but this may not be the case. Outlets may also specialise in multiple cuisines. It is possible to build a classifier that predicts multiple labels for a single takeaway outlet, and this should be explored in future work. However, at the time of this study, it was hard to assess model performance for multi-label models, as the ability to generate confusion matrices (which are necessary for development work in testing and refining model iterations) was limited.

We were unable to train our model to classify outlets with little representation (i.e. those with fewer than 422 outlets) in the training data, for example outlets labelled as Mexican. This means that they were not able to form a cusine type of their own, and that in practice these outlets would be assigned to another cuisine type. Outlets labelled as Mexican would probably be assigned to burger or multi fast food. However, although unknown, if Just Eat data are representative of the wider takeaway food sector in terms of cuisine type mix, the number of misclassified outlets in the latter would be relatively small. Future work might integrate other labelled data during model training, from additional online delivery platforms such as Deliveroo or Uber Eats (although there is likely to be significant overlap in records contained), or from other countries, in order to increase the amount of data available for training and prediction of less common cuisine types.

A common limitation of a deep neural network approach to classification, as used, is that it is hard to understand model performance i.e. why a model performs well in some instances and not others. For example, it is not easy to determine why we saw poorer performance with the burger cuisine type compared to others. Again, it is likely that the classifier would make more accurate predictions if it were given a larger amount of training data. Additional sources of training data might include unstructured text from menus or website HTML code, or business location from address data, as prevalence of outlets by cuisine type is likely to vary by region and neighbourhood socioeconomic status. Exploring the integration of such data to improve prediction accuracy will be the subject of future research. Importantly, larger volumes of data are unlikely to challenge typical computing resources. Moreover, since this work was completed, fast.ai platform version 2 has become available, offering enhanced model performance and incorporating the latest developments in deep learning.

## 6 Conclusions

In this study, we described the development of a new model to automatically classify takeaway food outlets, by 10 major cuisine types, from business name alone, using innovative data science methods. Although accuracy of prediction varied by cuisine type, overall this model was correct approximately three out of four times. As a case study of how the classifier could be used in combination with existing data for the purposes of knowledge generation, we provided a high-level description of the takeaway food outlet sector in England, constituting nearly 55,000 outlets, by cuisine type, for the first time. We have made the model publicly available for use by others for research and public health decision-making purposes, for tailoring to other contexts and for characterisation of other types of food outlets, and to permit further development and improvement.

## CRediT authorship contribution statement

**Tom R.P. Bishop:** Conceptualization, Methodology, Software, Validation, Formal analysis, Investigation, Data curation, Writing – Original draft, Visualisation, Funding acquisition. **Stephanie von Hinke:** Conceptualization, Writing – review & editing, Funding acquisition. **Bruce Hollingsworth:** Conceptualization, Writing – review & editing, Funding acquisition. **Amelia A. Lake:** Conceptualization, Writing – review & editing, Funding acquisition. **Heather Brown:** Conceptualization, Writing – review & editing, Funding acquisition. **Thomas Burgoine:** Conceptualization, Methodology, Formal analysis, Data curation, Writing – Original draft, Visualisation, Supervision, Project administration, Funding acquisition.

## Declaration of Competing Interest

The authors declare that they have no known competing financial interests or personal relationships that could have appeared to influence the work reported in this paper.

## Data Availability

The classifier code and the classified FSA FHRS data supporting the conclusions of this article are available on GitHub (https://github.com/tombisho/takeaways). We are making this code available with no restrictions on access, enabling the opportunity for collaborative development by the research community. The code has an introductory Readme file and the code contains comments on how to use it. The FSA FHRS data are published with an Open Government License that permits their copying, publishing, distribution, transmission, adaptation, and exploitation for both commercial and non-commercial applications, providing that the original source of the data is attributed (Food Standards Agency, 2020c) and where possible, providing a link to details of the Open Government License (Gov.uk, 2020). Data from Just Eat are not eligible for sharing.

## Appendix A Supplementary data

MMC S1

## References

[b1] Adams J., Goffe L., Brown T., Lake A.A., Summerbell C., White M., Adamson A.J. (2015). Frequency and socio-demographic correlates of eating meals out and take-away meals at home: cross-sectional analysis of the UK National Diet and Nutrition Survey, waves 1-4 (2008-12). International Journal of Behavioral Nutrition and Physical Activity.

[b2] Bagwell S. (2011). The role of independent fast-food outlets in obesogenic environments: a case study of east London in the UK. Environment and Planning A.

[b3] Black C., Moon G., Baird J. (2013). Dietary inequalities: what is the evidence for the effect of the neighbourhood food environment?. Health and Place.

[b4] Blow J., Gregg R., Davies I.G., Patel S. (2019). Type and density of independent takeaway outlets: a geographical mapping study in a low socioeconomic ward, Manchester. BMJ Open.

[b5] Bouvard V., Loomis D., Guyton K.Z., Grosse Y., Ghissassi F.E., Benbrahim-Tallaa L., ... A.J., Straif K., International Agency for Research on Cancer Monograph Working Group (2015). Cancinogenicity of consumption of red and processed meat. The Lancet Oncology.

[b6] Burgoine T., Forouhi N.G., Griffin S.J., Brage S., Wareham N.J., Monsivais P. (2016). Does neighborhood fast-food outlet exposure amplify inequalities in diet and obesity? A cross sectional study. American Journal of Clinical Nutrition.

[b7] Burgoine T., Forouhi N.G., Griffin S.J., Wareham N.J., Monsivais P. (2014). Associations between exposure to takeaway food outlets, takeaway food consumption, and body weight in Cambridgeshire, UK: population based, cross sectional study. BMJ.

[b8] Burgoine T., Monsivais P., the Feat Development Team S.J. (2017). http://www.feat-tool.org.uk.

[b9] Burgoine T., Sarkar C., Webster C., Monsivais P. (2018). Examining the interaction of fast-food outlet exposure and income on diet and obesity: evidence from 51, 361 UK Biobank participants. International Journal of Behavioral Nutrition and Physical Activity.

[b10] Cummins S., Clary C., Shareck M. (2017). Enduring challenges in estimating the effect of the food environment on obesity. American Journal of Clinical Nutrition.

[b11] Faltl S., Schimpke M., Hackober C. (2019). https://humboldt-wi.github.io/blog/research/information_systems_1819/group4_ulmfit/.

[b12] Fleischhacker S.E., Evenson K.R., Rodriguez D.A., Ammerman A.S. (2011). A systematic review of fast food access studies. Obesity Reviews.

[b13] Food Standards Agency (2020). https://api.ratings.food.gov.uk/help.

[b14] Food Standards Agency (2020). https://ratings.food.gov.uk/.

[b15] Food Standards Agency (2020). https://data.food.gov.uk/catalog/datasets/38dd8d6a-5ab1-4f50-b753-ab33288e3200.

[b16] Gov.uk (2020). http://www.nationalarchives.gov.uk/doc/open-government-licence/version/3/.

[b17] Howard, J., & Ruder, S. (2018). Universal language model fine-tuning for text classification. In *Proceedings of the 56th annual meeting of the assocation for computional linguistics* (pp. 328-339).

[b18] Hutcheon J.A., Chiolero A., Hanley J.A. (2010). Random measurement error and regression dilution bias. BMJ.

[b19] Intellectual Property Office of the UK Government (2014). https://www.gov.uk/guidance/exceptions-to-copyright.

[b20] Jaworowska A., Blackham T.M., Long R., Taylor C., Ashton M., Stevenson L., Davies I.G. (2014). Nutritional composition of takeaway food in the UK. Nutrition & Food Science.

[b21] Keeble M., Adams J., White M., Summerbell C., Cummins S., Burgoine T. (2019). Correlates of English local government use of the planning system to regulate hot food takeaway outlets. a cross-sectional analysis. International Journal of Behavioral Nutrition and Physical Activity.

[b22] Kirkman S., Hollingsworth B., Lake A., von Hinke S., Sorrell S., Burgoine T., Brown H. (2020). Field validity and spatial accuracy of food standards agency food hygiene rating scheme data for England. Journal of Public Health.

[b23] Lake A.A., Burgoine T., Greenhalgh F., Stamp E., Tyrrell R. (2010). The foodscape: classification and field validation of secondary data sources. Health and Place.

[b24] Lebel A., Daepp M.I.G., Block J.P., Walker R., Lalonde B., Kestens Y., Subramanian S.V. (2017). Quantifying the foodscape: a systematic review and meta-analysis of the validity of commercially available business data. PLoS One.

[b25] Lee, J., Kim, H., Ko, M., Choi, D., Choi, J., & Kang, J. (2017). Name nationality classification with recurrent neural networks. In *Proceedings of the twenty-sixth international joint conference on artificial intelligence* (pp. 2081-2087).

[b26] Micha R., Wallace S.K., Mozaffarian D. (2010). Red and processed meat consumption and risk of incident coronary heart disease, stroke, and diabetes mellitus. Circulation.

[b27] Micha R., Wallace S.K., Mozzaffarian D. (2011). Red and processed meat consumption and risk of incident heart disease, stroke, and diabetes: a systematic review and meta-analysis. Circulation.

[b28] Ministry of Housing, Communities & Local Government (2018).

[b29] Miura K., Giskes K., Turrell G. (2011). Socio-economic differences in takeaway food consumption among adults. Public Health Nutrition.

[b30] Monsivais P., Drewnowski A. (2007). The rising cost of low-energy-density foods. Journal of the American Dietetic Association.

[b31] Office for National Statistics (2019). https://www.ons.gov.uk/peoplepopulationandcommunity/populationandmigration/populationestimates/bulletins/annualmidyearpopulationestimates/mid2019estimates.

[b32] Paquet C., Daniel M., Kestens Y., Léger K., Gauvin L. (2008). Field validation of listings of food stores and commercial physical activity establishments from secondary data. International Journal of Behavioral Nutrition and Physical Activity.

[b33] Penney T., Jones N.R.V., Adams J., Maguire E.R., Burgoine T., Monsivais P. (2017). Utilization of away-from-home food establishments, dietary approaches to stop hypertension dietary pattern, and obesity. American Journal of Preventive Medicine.

[b34] Pereira M.A., Kartashov A.I., Ebbeling C.B., Van Horn L., Slattery M.L., Jacobs D.R., Ludwig D.S. (2005). Fast-food habits, weight gain, and insulin resistance (the CARDIA study): 15-year prospective analysis. The Lancet.

[b35] Public Health England (2018). https://www.gov.uk/government/publications/fast-food-outlets-density-by-local-authority-in-england.

[b36] Robinson E., Jones A., Whitelock V., Mead B.R., Haynes A. (2018). (Over)eating out at major UK restaurant chains: observational study of energy content of main meals. BMJ.

[b37] Ross E. (2018). https://gist.github.com/EdwardJRoss/86b31848a7951411de56f10f55e9de4e.

[b38] Shift (2013). http://www.shiftdesign.org.uk/content/uploads/2014/09/SHIFT_Chicken-Shop_Research_.pdf.

[b39] Shift (2018). https://shiftdesign.org/content/uploads/2017/05/MappingFastFoodHackney.pdf.

[b40] de Souza R.J., Mente A., Maroleanu A., Cozma A.I., Ha V., Kishibe T., ... D.S., Anand S.S. (2015). Intake of saturated and trans unsaturated fatty acids and risk of all cause mortality, cardiovascular disease, and type 2 diabetes: systematic review and meta-analysis of observational studies. BMJ.

[b41] Thompson C., Ponsford R., Lewis D., Cummins S. (2018). Fast-food, everyday life and health: a qualitative study of ’chicken shops’ in east London. Appetite.

[b42] Varghese A., Agyeman-Badu G., Cawley M. (2020). Deep learning in automated text classification: a case study using toxicological abstracts. Environment Systems and Decisions.

[b43] Wilkins E., Radley D., Morris M., Hobbs M., Christensen A., Marwa W.L., Griffiths C. (2019). A systematic review employing the GeoFERN framework to examine methods, reporting quality and associations between the retail food environment and obesity. Health and Place.

