# Supplementary material for: Automatic classification of takeaway food outlet cuisine type using machine (deep) learning
Source: Mach Learn Appl. 2021 Dec 15;6:None. doi: 10.1016/j.mlwa.2021.100106 (PMC8700226; doi:10.1016/j.mlwa.2021.100106)
Supplement: MMC S1 [file mmc1.docx]

**Additional Tables and Figures**

| **Table of contents** | **Page** |
| --- | --- |
| Table A1: Mapping of owner-selected cuisine labels (up to two labels per outlet) and business name data from Just Eat, to 10-point cuisine type classification system. | 2 |
| Table A2: Loss and accuracy results of classifier fine-tuning process. | 5 |
| Table A3: Recall and precision results for the naïve classifier, overall and by cuisine type. | 6 |
| Table A4: Recall and precision results for the six-point classifier, overall and by cuisine type. | 7 |
| Table A5: Summary data for local authorities and England. | 8 |
| Fig A1: Confusion matrix, showing specific instances of misclassification, according to the naïve classifier. Rows total to 400 outlets. | 19 |
| Fig A2: Confusion matrix, showing specific instances of misclassification, according to the six-point classifier. Rows total to 400 outlets. | 20 |
| Fig A3: Number of burger outlets per local authority per 100,000 population (quintiles (Q)). Excludes the City of London as an extreme outlier, with 318.9 outlets / 100,000 population. | 21 |
| Fig A4: Number of chicken outlets per local authority per 100,000 population (quintiles (Q)). Excludes the City of London as an extreme outlier, with 113.2 outlets / 100,000 population. | 22 |
| Fig A5: Number of dessert outlets per local authority per 100,000 population (quintiles (Q)). Excludes the City of London as an extreme outlier, with 41.1 outlets / 100,000 population. | 23 |
| Fig A6: Number of multi fast-food outlets per local authority per 100,000 population (quintiles (Q)). | 24 |
| Fig A7: Number of fish & chip shops per local authority per 100,000 population (quintiles (Q)). Excludes the City of London as an extreme outlier, with 298.3 outlets / 100,000 population. | 25 |
| Fig A8: Number of kebab shops per local authority per 100,000 population (quintiles (Q)). Excludes the City of London as an extreme outlier, with 185.2 outlets / 100,000 population. | 26 |
| Fig A9: Number of pizza outlets per local authority per 100,000 population (quintiles (Q)). Excludes the City of London as an extreme outlier, with 421.8 outlets / 100,000 population. | 27 |
| Fig A10: Number of sandwich shops/cafés/bakeries per local authority per 100,000 population (quintiles (Q)). Excludes the City of London as an extreme outlier, with 1172.7 outlets / 100,000 population. | 28 |
| Fig A11: Number of South Asian outlets per local authority per 100,000 population (quintiles (Q)). Excludes the City of London as an extreme outlier, with 761.2 outlets / 100,000 population. | 29 |
| Fig A12: Number of Southeast & East Asian outlets per local authority per 100,000 population (quintiles (Q)). Excludes the City of London as an extreme outlier, with 586.4 outlets / 100,000 population. | 30 |

Table A1: Mapping of owner-selected cuisine labels (up to two labels per outlet) and business name data from Just Eat, to 10-point cuisine type classification system.

| **10-point Cuisine type, n (% outlets)** | **Just Eat owner-classification, n (% labels)** | **Business name, n (% outlets)** |
| --- | --- | --- |
| South Asian, 4,439 (23%) | Indian, 4,379 (11.32%)  Curry, 2,788 (7.21%)  Bangladeshi, 473 (1.22%)  Nepalese, 111 (0.29%)  Pakistani, 100 (0.26%)  South Indian, 45 (0.12%)  Sri-lankan, 28 (0.07%)  Balti, 7 (0.02%)  Punjabi, 7 (0.02%)  Biryani, 8 (0.02%) | n/a |
| Southeast & East Asian, 2,790 (14%) | Chinese, 2,246 (5.81%)  Oriental, 1,416 (3.66%)  Thai, 512 (1.32%)  Japanese, 325 (0.84%)  Noodles, 209 (0.54%)  Sushi, 205 (0.53%)  Vietnamese, 54 (0.14%)  Malaysian, 46 (0.12%)  Korean, 38 (0.10%)  Bubble Tea, 34 (0.09%)  Cantonese, 30 (0.08%)  Dim Sum, 21 (0.05%)  Singapore, 2 (0.01%)  Filipino, 5 (0.01%)  Taiwanese, 1 (0.00%) | n/a |
| Chicken, 1,111 (6%) | Chicken, 1,462 (3.78%)  Peri Peri, 480 (1.24%) | Chicken, 670 (3%) |
| Kebab, 1,438 (7%) | Kebab, 3,413 (8.82%)  Turkish, 425 (1.10%) | Kebab, 1,085 (6%) |
| Burger, 1,021 (5%) | Burgers, 1,808 (4.67%)  American, 683 (1.77%)  Gourmet Burgers, 28 (0.07%) | Burger, 323 (2%) |
| Sandwich / Cafe / Bakery, 1,001 (5%) | Breakfast, 851 (2.20%)  Sandwiches, 312 (0.81%)  Café, 257 (0.66%)  Deli, 63 (0.16%)  Wraps, 39 (0.10%)  Salads, 40 (0.10%)  Panini's, 27 (0.07%)  Bagels, 24 (0.06%)  Baguettes, 4 (0.01%) |  |
| Fish and chips, 1,354 (7%) | Fish & Chips, 1,635 (4.23%) | n/a |
| Desserts, 719 (4%) | Desserts, 909 (2.35%)  Ice Cream, 221 (0.57%)  Milkshakes, 163 (0.42%)  Cakes, 153 (0.40%)  Waffles, 33 (0.09%)  Crepes, 23 (0.06%) | n/a |
| Pizza, 3,450 (18%) | Pizza, 5,316 (13.74%)  Italian, 1,576 (4.07%)  Italian Pizza, 20 (0.05%) | Pizza, 2,369 (12%) |
| Multi fast food, 822 (4%) | n/a | More than one of the following (772 (4%)): Chicken, Burger, Kebab, Pizza, Fast food |
| Not used for the purposes of classification, 1,286 (7%) | Grill, 698 (1.80%); Caribbean, 423 (1.09%); British, 371 (0.96%); English, 344 (0.89%); Mediterranean, 322 (0.83%); Asian, 217 (0.56%); Lebanese, 206 (0.53%); Fast Food, 201 (0.52%); Halal, 188 (0.49%); Mexican, 189 (0.49%); Vegan, 187 (0.48%); Jamaican, 181 (0.47%); Vegetarian, 157 (0.41%); Healthy, 151 (0.39%); Pasta, 143 (0.37%); Lunch, 134 (0.35%); African, 119 (0.31%); Greek, 120 (0.31%); Middle Eastern, 108 (0.28%); Persian, 84 (0.22%); European, 72 (0.19%); Gourmet, 71 (0.18%); BBQ, 54 (0.14%); Steak, 54 (0.14%); Drinks, 38 (0.10%); Afghan, 33 (0.09%); Fusion, 34 (0.09%); Arabic, 34 (0.09%); Spanish, 35 (0.09%); Nigerian, 36 (0.09%); Seafood, 36 (0.09%); Street Food, 32 (0.08%); Burritos, 32 (0.08%); Iranian, 29 (0.07%); French, 22 (0.06%); Dinner, 25 (0.06%); Portuguese, 25 (0.06%); Smoothies, 25 (0.06%); Tex Mex, 19 (0.05%); Sweets, 18 (0.05%); Tapas, 18 (0.05%); Moroccan, 21 (0.05%); English Breakfast, 14 (0.04%); Jerk, 15 (0.04%); Roast Dinners, 15 (0.04%); Romanian, 15 (0.04%); None, 16 (0.04%); Gluten Free, 16 (0.04%); Parmesans, 17 (0.04%); Polish, 17 (0.04%); Brazilian food, 11 (0.03%); Brunch, 12 (0.03%); South American, 12 (0.03%); Eastern European, 13 (0.03%); Indo-Chinese Fusion, 7 (0.02%); Pub Food, 7 (0.02%); Russian, 7 (0.02%); Ethiopian, 7 (0.02%); Kurdish, 8 (0.02%); Continental, 8 (0.02%); Pancakes, 8 (0.02%); Latin American, 9 (0.02%); Hot Dogs, 9 (0.02%); Syrian, 9 (0.02%); North African, 2 (0.01%); Pies, 5 (0.01%); Organic, 2 (0.01%); Kosher, 2 (0.01%); Hungarian, 2 (0.01%); Frozen Yogurt, 2 (0.01%); Peruvian, 2 (0.01%); South African, 2 (0.01%); German, 2 (0.01%); Colombian, 2 (0.01%); Authentic Pizza, 3 (0.01%); Bulgarian, 3 (0.01%); Scottish, 3 (0.01%); West African, 3 (0.01%); Retro Sweets, 3 (0.01%); Alcohol, 4 (0.01%); Argentinian, 4 (0.01%); Soup, 4 (0.01%); Egyptian, 5 (0.01%); Ukrainian, 1 (0.00%); Rotisserie, 1 (0.00%); Danish, 1 (0.00%); Sizzlers, 1 (0.00%); NEW, 1 (0.00%); Subways, 1 (0.00%); Low-Carb, 1 (0.00%); Belgian Waffles, 1 (0.00%); Best Bites, 1 (0.00%); Tex-Mex, 1 (0.00%); Groceries, 1 (0.00%); Ghanaian, 1 (0.00%); Iraqi, 1 (0.00%); Salt & Pepper, 1 (0.00%) | n/a |

Table A2: Loss and accuracy results of classifier fine-tuning process.

| **Epoch** | **Training loss** | **Validation Loss** | **Validation Accuracy** |
| --- | --- | --- | --- |
| 0 | 1.46 | 1.14 | 64% |
| 1 | 1.13 | 0.99 | 68% |
| 2 | 1.06 | 0.98 | 69% |
| 3 | 0.94 | 1.00 | 69% |
| 4 | 0.94 | 0.99 | 70% |
| 5 | 0.89 | 0.98 | 70% |
| 6 | 0.89 | 1.01 | 71% |
| 7 | 0.86 | 0.96 | 71% |
| 8 | 0.83 | 0.98 | 72% |
| 9 | 0.71 | 1.01 | 72% |
| 10 | 0.71 | 1.02 | 72% |
| 11 | 0.67 | 1.00 | 72% |
| 12 | 0.67 | 1.02 | 72% |
| 13 | 0.58 | 1.06 | 73% |
| 14 | 0.56 | 1.06 | 72% |
| 15 | 0.48 | 1.11 | 73% |
| 16 | 0.50 | 1.08 | 72% |
| 17 | 0.46 | 1.11 | 72% |
| 18 | 0.47 | 1.09 | 72% |
| 19 | 0.47 | 1.12 | 72% |

Table A3: Recall and precision results for the naïve classifier, overall and by cuisine type.

| **Cuisine type** | **Recall, %** | **Precision, %** |
| --- | --- | --- |
| Burger | 33 | 61 |
| Sandwich/Cafe/Bakery | 53 | 57 |
| Chicken | 57 | 71 |
| Desserts | 64 | 71 |
| Kebab | 51 | 46 |
| Pizza | 57 | 66 |
| Fish & Chips | 73 | 60 |
| South Asian | 68 | 75 |
| Southeast & East Asian | 68 | 67 |
| Multi fast food | 74 | 43 |
| **Overall** | **60** | **62** |

Table A4: Recall and precision results for the six-point classifier, overall and by cuisine type.

| **Cuisine type** | **Recall, %** | **Precision, %** |
| --- | --- | --- |
| Sandwich/Cafe/Bakery | 61 | 78 |
| Desserts | 67 | 89 |
| Fish & Chips | 80 | 90 |
| South Asian | 82 | 82 |
| Southeast & East Asian | 86 | 80 |
| Multi fast food | 85 | 56 |
| **Overall** | **77** | **79** |

| Table A5: Summary data for local authorities and England. | | | | | | | | | | | |
| --- | --- | --- | --- | --- | --- | --- | --- | --- | --- | --- | --- |
|  | **Outlets (n (%)) per 100,000 population by cuisine type** | | | | | | | | | | |
| **Local Authority** | **Burger** | **Chicken** | **Desserts** | **Fast food** | **Fish & Chips** | **Kebab** | **Pizza** | **Sand/Caf/Bake** | **S Asian** | **SE & E Asian** | **Overall** |
| Adur | 10.9 (12.3) | 6.2 (7.0) | 1.6 (1.8) | 1.6 (1.8) | 15.6 (17.5) | 6.2 (7.0) | 7.8 (8.8) | 4.7 (5.3) | 14.0 (15.8) | 20.2 (22.8) | 88.6 |
| Allerdale | 4.1 (4.1) | 0.0 (0.0) | 6.1 (6.1) | 1.0 (1.0) | 17.4 (17.3) | 7.2 (7.1) | 14.3 (14.3) | 21.5 (21.4) | 10.2 (10.2) | 18.4 (18.4) | 100.2 |
| Amber Valley | 5.5 (6.1) | 3.1 (3.5) | 2.3 (2.6) | 0.8 (0.9) | 17.2 (19.1) | 0.8 (0.9) | 14.8 (16.5) | 12.5 (13.9) | 7.8 (8.7) | 25.0 (27.8) | 89.7 |
| Arun | 9.3 (10.3) | 5.6 (6.2) | 1.9 (2.1) | 3.7 (4.1) | 15.6 (17.2) | 4.4 (4.8) | 11.8 (13.1) | 8.1 (9.0) | 14.3 (15.9) | 15.6 (17.2) | 90.2 |
| Ashfield | 4.7 (5.1) | 1.6 (1.7) | 0.8 (0.9) | 4.7 (5.1) | 20.3 (22.2) | 0.0 (0.0) | 16.4 (17.9) | 11.7 (12.8) | 7.0 (7.7) | 24.2 (26.5) | 91.5 |
| Ashford | 8.5 (13.4) | 3.8 (6.1) | 0.8 (1.2) | 1.5 (2.4) | 12.3 (19.5) | 6.2 (9.8) | 6.9 (11.0) | 3.8 (6.1) | 6.9 (11.0) | 12.3 (19.5) | 63.1 |
| Aylesbury Vale | 4.0 (6.6) | 4.5 (7.4) | 1.5 (2.5) | 1.0 (1.7) | 11.0 (18.2) | 6.5 (10.7) | 8.5 (14.0) | 6.0 (9.9) | 5.0 (8.3) | 12.5 (20.7) | 60.7 |
| Babergh | 4.3 (7.5) | 3.3 (5.7) | 2.2 (3.8) | 2.2 (3.8) | 7.6 (13.2) | 3.3 (5.7) | 12.0 (20.8) | 3.3 (5.7) | 9.8 (17.0) | 9.8 (17.0) | 57.6 |
| Barking and Dagenham | 5.6 (6.4) | 13.6 (15.4) | 0.9 (1.1) | 4.2 (4.8) | 11.3 (12.8) | 8.9 (10.1) | 8.9 (10.1) | 8.5 (9.6) | 9.4 (10.6) | 16.9 (19.1) | 88.3 |
| Barnet | 5.8 (8.0) | 10.6 (14.7) | 1.3 (1.7) | 1.3 (1.7) | 6.3 (8.7) | 6.1 (8.4) | 13.6 (18.9) | 7.3 (10.1) | 8.3 (11.5) | 11.6 (16.1) | 72.2 |
| Barnsley | 7.3 (6.2) | 7.7 (6.6) | 1.6 (1.4) | 4.1 (3.4) | 27.1 (23.1) | 2.4 (2.1) | 19.4 (16.6) | 13.8 (11.7) | 10.5 (9.0) | 23.5 (20.0) | 117.5 |
| Barrow-in-Furness | 11.9 (9.9) | 3.0 (2.5) | 1.5 (1.2) | 3.0 (2.5) | 20.9 (17.3) | 6.0 (4.9) | 14.9 (12.3) | 16.4 (13.6) | 10.4 (8.6) | 32.8 (27.2) | 120.8 |
| Basildon | 10.1 (10.9) | 6.4 (6.9) | 2.1 (2.3) | 4.8 (5.1) | 11.2 (12.0) | 4.3 (4.6) | 11.8 (12.6) | 13.4 (14.3) | 14.4 (15.4) | 15.0 (16.0) | 93.5 |
| Basingstoke and Deane | 4.5 (8.4) | 3.4 (6.3) | 0.6 (1.1) | 0.6 (1.1) | 4.5 (8.4) | 6.2 (11.6) | 10.2 (18.9) | 6.8 (12.6) | 6.8 (12.6) | 10.2 (18.9) | 53.8 |
| Bassetlaw | 4.3 (4.4) | 6.0 (6.1) | 0.9 (0.9) | 0.9 (0.9) | 23.0 (23.7) | 6.0 (6.1) | 17.0 (17.5) | 14.5 (14.9) | 6.0 (6.1) | 18.7 (19.3) | 97.1 |
| Bath and North East Somerset | 6.7 (9.0) | 4.7 (6.2) | 1.6 (2.1) | 1.0 (1.4) | 12.9 (17.2) | 2.1 (2.8) | 11.9 (15.9) | 9.3 (12.4) | 10.3 (13.8) | 14.5 (19.3) | 75.0 |
| Bedford | 9.2 (10.0) | 8.1 (8.8) | 3.5 (3.7) | 1.2 (1.2) | 15.6 (16.9) | 3.5 (3.7) | 14.4 (15.6) | 9.2 (10.0) | 13.3 (14.4) | 14.4 (15.6) | 92.3 |
| Bexley | 4.0 (4.9) | 7.2 (8.8) | 1.2 (1.5) | 2.4 (2.9) | 13.7 (16.6) | 8.9 (10.7) | 10.1 (12.2) | 6.4 (7.8) | 9.7 (11.7) | 18.9 (22.9) | 82.6 |
| Birmingham | 6.1 (8.6) | 8.6 (12.0) | 2.8 (3.9) | 1.4 (2.0) | 9.0 (12.7) | 4.9 (6.9) | 10.1 (14.1) | 8.1 (11.3) | 9.1 (12.8) | 11.2 (15.7) | 71.3 |
| Blaby | 6.9 (10.4) | 4.9 (7.5) | 1.0 (1.5) | 1.0 (1.5) | 12.8 (19.4) | 0.0 (0.0) | 10.8 (16.4) | 5.9 (9.0) | 5.9 (9.0) | 16.7 (25.4) | 66.0 |
| Blackburn | 10.7 (7.7) | 10.0 (7.2) | 4.7 (3.4) | 4.0 (2.9) | 18.7 (13.5) | 8.7 (6.3) | 16.0 (11.6) | 18.0 (13.0) | 26.1 (18.8) | 21.4 (15.5) | 138.3 |
| Blackpool | 20.1 (9.2) | 7.9 (3.6) | 7.9 (3.6) | 3.6 (1.6) | 42.3 (19.4) | 13.6 (6.2) | 30.8 (14.1) | 39.4 (18.1) | 16.5 (7.6) | 35.9 (16.4) | 218.0 |
| Bolsover | 12.4 (12.5) | 3.7 (3.8) | 5.0 (5.0) | 2.5 (2.5) | 16.1 (16.2) | 7.4 (7.5) | 14.9 (15.0) | 3.7 (3.8) | 9.9 (10.0) | 23.6 (23.7) | 99.3 |
| Bolton | 10.4 (7.9) | 12.2 (9.2) | 4.9 (3.7) | 1.7 (1.3) | 18.1 (13.6) | 6.3 (4.7) | 18.8 (14.2) | 13.9 (10.5) | 20.5 (15.5) | 25.7 (19.4) | 132.5 |
| Boston | 10.0 (12.1) | 2.9 (3.4) | 1.4 (1.7) | 1.4 (1.7) | 24.2 (29.3) | 5.7 (6.9) | 10.0 (12.1) | 12.8 (15.5) | 4.3 (5.2) | 10.0 (12.1) | 82.7 |
| Bournemouth, Christchurch and Poole | 10.4 (9.5) | 10.9 (10.0) | 1.0 (0.9) | 1.8 (1.6) | 15.7 (14.4) | 4.8 (4.4) | 19.2 (17.6) | 13.4 (12.3) | 9.6 (8.8) | 22.5 (20.6) | 109.3 |
| Bracknell Forest | 1.6 (2.9) | 1.6 (2.9) | 0.8 (1.5) | 0.8 (1.5) | 6.5 (11.8) | 9.8 (17.6) | 10.6 (19.1) | 7.3 (13.2) | 4.9 (8.8) | 11.4 (20.6) | 55.5 |
|  | **Outlets (n (%)) per 100,000 population by cuisine type** | | | | | | | | | | |
| **Local Authority** | **Burger** | **Chicken** | **Desserts** | **Fast food** | **Fish & Chips** | **Kebab** | **Pizza** | **Sand/Caf/Bake** | **S Asian** | **SE & E Asian** | **Overall** |
| Bradford | 12.4 (9.0) | 9.8 (7.2) | 3.7 (2.7) | 2.4 (1.8) | 20.9 (15.2) | 5.7 (4.2) | 21.9 (15.9) | 20.7 (15.1) | 20.7 (15.1) | 18.9 (13.8) | 137.3 |
| Braintree | 5.9 (7.9) | 2.0 (2.6) | 1.3 (1.8) | 2.6 (3.5) | 13.8 (18.4) | 4.6 (6.1) | 11.1 (14.9) | 5.9 (7.9) | 10.5 (14.0) | 17.0 (22.8) | 74.7 |
| Breckland | 7.1 (11.8) | 3.6 (5.9) | 0.0 (0.0) | 1.4 (2.4) | 15.7 (25.9) | 5.0 (8.2) | 4.3 (7.1) | 5.0 (8.2) | 5.0 (8.2) | 13.6 (22.4) | 60.7 |
| Brent | 10.0 (9.3) | 17.9 (16.6) | 4.9 (4.5) | 2.4 (2.3) | 6.4 (5.9) | 6.4 (5.9) | 19.7 (18.3) | 12.4 (11.5) | 15.8 (14.6) | 11.8 (11.0) | 107.7 |
| Brentwood | 5.2 (6.2) | 2.6 (3.1) | 2.6 (3.1) | 0.0 (0.0) | 11.7 (13.8) | 10.4 (12.3) | 11.7 (13.8) | 9.1 (10.8) | 16.9 (20.0) | 14.3 (16.9) | 84.4 |
| Brighton and Hove | 16.2 (13.5) | 6.9 (5.7) | 3.4 (2.9) | 1.7 (1.4) | 13.4 (11.2) | 9.3 (7.8) | 20.3 (17.0) | 14.4 (12.1) | 16.8 (14.1) | 17.2 (14.4) | 119.6 |
| Bristol | 9.9 (9.1) | 8.8 (8.2) | 1.3 (1.2) | 3.9 (3.6) | 11.0 (10.1) | 9.5 (8.7) | 17.5 (16.1) | 14.9 (13.7) | 13.2 (12.1) | 18.6 (17.1) | 108.6 |
| Broadland | 3.8 (8.3) | 2.3 (5.0) | 0.0 (0.0) | 2.3 (5.0) | 14.5 (31.7) | 1.5 (3.3) | 3.1 (6.7) | 1.5 (3.3) | 5.4 (11.7) | 11.5 (25.0) | 45.9 |
| Bromley | 6.3 (7.9) | 5.4 (6.8) | 0.3 (0.4) | 2.4 (3.0) | 11.4 (14.3) | 6.9 (8.7) | 12.0 (15.1) | 7.2 (9.1) | 10.2 (12.8) | 17.5 (21.9) | 79.7 |
| Bromsgrove | 12.0 (12.9) | 6.0 (6.5) | 0.0 (0.0) | 1.0 (1.1) | 15.0 (16.1) | 2.0 (2.2) | 13.0 (14.0) | 15.0 (16.1) | 12.0 (12.9) | 17.0 (18.3) | 93.1 |
| Broxbourne | 6.2 (6.3) | 7.2 (7.3) | 4.1 (4.2) | 3.1 (3.1) | 17.5 (17.7) | 6.2 (6.3) | 18.5 (18.7) | 10.3 (10.4) | 6.2 (6.3) | 19.5 (19.8) | 98.7 |
| Broxtowe | 7.0 (7.5) | 3.5 (3.8) | 1.8 (1.9) | 2.6 (2.8) | 16.7 (17.9) | 4.4 (4.7) | 17.5 (18.9) | 10.5 (11.3) | 12.3 (13.2) | 16.7 (17.9) | 93.0 |
| Burnley | 11.2 (6.6) | 13.5 (7.9) | 3.4 (2.0) | 3.4 (2.0) | 23.6 (13.8) | 9.0 (5.3) | 24.7 (14.5) | 29.2 (17.1) | 20.2 (11.8) | 32.6 (19.1) | 170.9 |
| Bury | 11.5 (8.5) | 8.9 (6.5) | 2.6 (1.9) | 1.0 (0.8) | 20.4 (15.0) | 4.2 (3.1) | 22.0 (16.2) | 19.4 (14.2) | 19.9 (14.6) | 26.2 (19.2) | 136.1 |
| Calderdale | 11.3 (8.1) | 9.9 (7.1) | 1.4 (1.0) | 2.4 (1.7) | 23.6 (16.9) | 4.3 (3.1) | 20.8 (14.9) | 24.1 (17.3) | 24.1 (17.3) | 17.5 (12.5) | 139.5 |
| Cambridge City | 10.4 (10.5) | 7.2 (7.3) | 4.8 (4.8) | 0.8 (0.8) | 11.2 (11.3) | 11.2 (11.3) | 12.0 (12.1) | 18.4 (18.5) | 7.2 (7.3) | 16.0 (16.1) | 99.4 |
| Camden | 25.9 (15.0) | 12.6 (7.3) | 5.9 (3.4) | 0.7 (0.4) | 9.3 (5.4) | 10.4 (6.0) | 25.2 (14.6) | 29.6 (17.2) | 21.8 (12.7) | 31.1 (18.0) | 172.6 |
| Cannock Chase | 9.9 (8.3) | 4.0 (3.3) | 0.0 (0.0) | 1.0 (0.8) | 26.8 (22.3) | 6.0 (5.0) | 11.9 (9.9) | 16.9 (14.0) | 14.9 (12.4) | 28.8 (24.0) | 120.1 |
| Canterbury City | 7.3 (8.3) | 4.2 (4.8) | 3.0 (3.4) | 1.8 (2.1) | 17.5 (20.0) | 4.2 (4.8) | 16.3 (18.6) | 7.9 (9.0) | 6.7 (7.6) | 18.7 (21.4) | 87.7 |
| Carlisle City | 7.4 (7.5) | 3.7 (3.8) | 0.9 (0.9) | 0.0 (0.0) | 14.7 (15.1) | 5.5 (5.7) | 19.3 (19.8) | 14.7 (15.1) | 8.3 (8.5) | 23.0 (23.6) | 97.5 |
| Castle Point | 6.6 (7.2) | 7.7 (8.4) | 2.2 (2.4) | 3.3 (3.6) | 14.4 (15.7) | 7.7 (8.4) | 11.1 (12.0) | 6.6 (7.2) | 11.1 (12.0) | 21.0 (22.9) | 91.8 |
| Central Bedfordshire | 4.2 (6.0) | 5.9 (8.5) | 0.3 (0.5) | 2.8 (4.0) | 11.4 (16.6) | 6.6 (9.5) | 9.4 (13.6) | 5.9 (8.5) | 8.7 (12.6) | 13.9 (20.1) | 68.9 |
| Charnwood | 5.4 (6.8) | 2.2 (2.7) | 2.2 (2.7) | 0.0 (0.0) | 12.4 (15.5) | 4.3 (5.4) | 18.8 (23.6) | 8.1 (10.1) | 11.3 (14.2) | 15.1 (18.9) | 79.6 |
| Chelmsford | 5.0 (7.5) | 3.9 (5.8) | 1.7 (2.5) | 0.6 (0.8) | 5.0 (7.5) | 2.8 (4.2) | 9.5 (14.2) | 11.2 (16.7) | 13.5 (20.0) | 14.0 (20.8) | 67.3 |
| Cheltenham | 6.0 (6.4) | 7.7 (8.3) | 1.7 (1.8) | 2.6 (2.8) | 15.5 (16.5) | 6.9 (7.3) | 12.9 (13.8) | 9.5 (10.1) | 15.5 (16.5) | 15.5 (16.5) | 93.7 |
| Cherwell | 8.6 (9.8) | 5.3 (6.1) | 1.3 (1.5) | 2.7 (3.0) | 13.3 (15.2) | 4.0 (4.5) | 16.6 (18.9) | 12.0 (13.6) | 5.3 (6.1) | 18.6 (21.2) | 87.7 |
| Cheshire East | 5.2 (6.0) | 4.2 (4.8) | 0.3 (0.3) | 1.8 (2.1) | 14.6 (16.7) | 4.4 (5.1) | 12.0 (13.7) | 11.5 (13.1) | 14.1 (16.1) | 19.5 (22.3) | 87.5 |
| Cheshire West and Chester | 6.4 (7.0) | 5.8 (6.4) | 1.7 (1.9) | 2.6 (2.9) | 13.1 (14.3) | 5.5 (6.1) | 14.0 (15.3) | 14.3 (15.6) | 9.0 (9.9) | 18.9 (20.7) | 91.5 |
| Chesterfield | 7.6 (5.2) | 8.6 (5.8) | 1.0 (0.6) | 3.8 (2.6) | 24.8 (16.9) | 3.8 (2.6) | 24.8 (16.9) | 27.6 (18.8) | 12.4 (8.4) | 32.4 (22.1) | 146.8 |
|  | **Outlets (n (%)) per 100,000 population by cuisine type** | | | | | | | | | | |
| **Local Authority** | **Burger** | **Chicken** | **Desserts** | **Fast food** | **Fish & Chips** | **Kebab** | **Pizza** | **Sand/Caf/Bake** | **S Asian** | **SE & E Asian** | **Overall** |
| Chichester | 10.7 (15.3) | 3.3 (4.7) | 0.8 (1.2) | 0.8 (1.2) | 14.9 (21.2) | 4.1 (5.9) | 9.1 (12.9) | 5.8 (8.2) | 9.1 (12.9) | 11.6 (16.5) | 70.2 |
| Chiltern | 3.1 (5.7) | 3.1 (5.7) | 0.0 (0.0) | 0.0 (0.0) | 9.4 (17.0) | 7.3 (13.2) | 9.4 (17.0) | 7.3 (13.2) | 7.3 (13.2) | 8.3 (15.1) | 55.3 |
| Chorley | 12.7 (11.1) | 7.6 (6.7) | 3.4 (3.0) | 0.0 (0.0) | 16.1 (14.1) | 5.1 (4.4) | 16.9 (14.8) | 18.6 (16.3) | 15.2 (13.3) | 18.6 (16.3) | 114.2 |
| City of London Corporation | 318.9 (8.2) | 113.2 (2.9) | 41.1 (1.1) | 0.0 (0.0) | 298.3 (7.7) | 185.2 (4.7) | 421.8 (10.8) | 1172.7 (30.1) | 761.2 (19.5) | 586.4 (15.0) | 3898.8 |
| Colchester | 5.1 (7.5) | 4.1 (6.0) | 0.5 (0.7) | 1.5 (2.2) | 11.8 (17.2) | 3.1 (4.5) | 9.2 (13.4) | 7.7 (11.2) | 9.2 (13.4) | 16.4 (23.9) | 68.8 |
| Copeland | 7.3 (7.1) | 4.4 (4.3) | 1.5 (1.4) | 1.5 (1.4) | 19.1 (18.6) | 5.9 (5.7) | 19.1 (18.6) | 13.2 (12.9) | 11.7 (11.4) | 19.1 (18.6) | 102.7 |
| Corby | 4.2 (9.1) | 2.8 (6.1) | 0.0 (0.0) | 1.4 (3.0) | 8.3 (18.2) | 6.9 (15.2) | 8.3 (18.2) | 8.3 (18.2) | 1.4 (3.0) | 4.2 (9.1) | 45.7 |
| Cornwall | 8.4 (10.1) | 2.8 (3.4) | 2.6 (3.1) | 1.1 (1.3) | 18.3 (21.8) | 4.7 (5.7) | 10.2 (12.2) | 12.5 (14.9) | 6.8 (8.2) | 16.3 (19.5) | 83.7 |
| Cotswold | 4.5 (7.1) | 1.1 (1.8) | 0.0 (0.0) | 1.1 (1.8) | 13.4 (21.4) | 6.7 (10.7) | 8.9 (14.3) | 11.1 (17.9) | 7.8 (12.5) | 7.8 (12.5) | 62.3 |
| Coventry | 7.0 (7.1) | 8.1 (8.2) | 1.1 (1.1) | 1.3 (1.4) | 15.1 (15.4) | 3.5 (3.6) | 14.8 (15.1) | 17.0 (17.3) | 13.7 (14.0) | 16.4 (16.8) | 98.0 |
| Craven | 5.3 (5.6) | 1.8 (1.9) | 1.8 (1.9) | 1.8 (1.9) | 24.5 (25.9) | 1.8 (1.9) | 14.0 (14.8) | 12.3 (13.0) | 17.5 (18.5) | 14.0 (14.8) | 94.5 |
| Crawley | 10.7 (13.5) | 5.3 (6.7) | 0.9 (1.1) | 0.0 (0.0) | 10.7 (13.5) | 6.2 (7.9) | 10.7 (13.5) | 14.2 (18.0) | 9.8 (12.4) | 10.7 (13.5) | 79.2 |
| Croydon | 8.3 (8.3) | 19.4 (19.5) | 1.0 (1.0) | 1.6 (1.6) | 8.0 (8.1) | 11.6 (11.7) | 16.5 (16.7) | 8.8 (8.9) | 8.8 (8.9) | 15.3 (15.4) | 99.3 |
| Dacorum | 5.8 (7.8) | 3.9 (5.2) | 2.6 (3.4) | 2.6 (3.4) | 11.0 (14.7) | 7.1 (9.5) | 12.3 (16.4) | 9.0 (12.1) | 9.7 (12.9) | 11.0 (14.7) | 75.0 |
| Darlington | 8.4 (6.0) | 2.8 (2.0) | 6.6 (4.7) | 0.0 (0.0) | 24.3 (17.3) | 6.6 (4.7) | 30.0 (21.3) | 23.4 (16.7) | 12.2 (8.7) | 26.2 (18.7) | 140.4 |
| Dartford | 9.8 (10.6) | 6.2 (6.7) | 2.7 (2.9) | 0.9 (1.0) | 15.1 (16.3) | 10.7 (11.5) | 11.5 (12.5) | 9.8 (10.6) | 8.9 (9.6) | 16.9 (18.3) | 92.4 |
| Daventry | 7.0 (11.1) | 0.0 (0.0) | 0.0 (0.0) | 1.2 (1.9) | 11.6 (18.5) | 2.3 (3.7) | 9.3 (14.8) | 8.1 (13.0) | 11.6 (18.5) | 11.6 (18.5) | 62.8 |
| Derby City | 8.2 (7.1) | 7.0 (6.1) | 4.7 (4.0) | 4.7 (4.0) | 15.5 (13.5) | 5.4 (4.7) | 17.9 (15.5) | 17.1 (14.8) | 12.4 (10.8) | 22.5 (19.5) | 115.4 |
| Derbyshire Dales | 4.1 (5.5) | 0.0 (0.0) | 1.4 (1.8) | 2.8 (3.6) | 27.7 (36.4) | 0.0 (0.0) | 9.7 (12.7) | 11.1 (14.5) | 4.1 (5.5) | 15.2 (20.0) | 76.0 |
| Doncaster | 9.3 (7.5) | 3.2 (2.6) | 1.9 (1.6) | 0.6 (0.5) | 20.8 (16.8) | 4.2 (3.4) | 27.6 (22.2) | 17.0 (13.7) | 12.8 (10.3) | 26.6 (21.4) | 124.1 |
| Dorset | 5.8 (7.1) | 3.4 (4.2) | 3.2 (3.9) | 3.2 (3.9) | 14.0 (17.0) | 5.5 (6.8) | 12.2 (14.8) | 12.2 (14.8) | 6.9 (8.4) | 15.9 (19.3) | 82.2 |
| Dover | 6.8 (7.1) | 5.9 (6.3) | 0.0 (0.0) | 3.4 (3.6) | 17.8 (18.8) | 5.9 (6.3) | 16.1 (17.0) | 9.3 (9.8) | 7.6 (8.0) | 22.0 (23.2) | 94.8 |
| Dudley | 7.2 (7.2) | 7.8 (7.9) | 2.2 (2.2) | 2.5 (2.5) | 22.7 (23.0) | 2.2 (2.2) | 15.5 (15.7) | 11.5 (11.6) | 8.1 (8.2) | 19.3 (19.5) | 98.9 |
| Durham | 5.8 (4.9) | 2.6 (2.2) | 1.7 (1.4) | 0.6 (0.5) | 24.0 (19.9) | 2.6 (2.2) | 28.9 (24.0) | 17.2 (14.3) | 13.6 (11.3) | 23.4 (19.4) | 120.4 |
| Ealing | 9.9 (12.5) | 11.1 (14.0) | 0.9 (1.1) | 2.9 (3.7) | 6.4 (8.1) | 7.6 (9.6) | 13.5 (16.9) | 9.1 (11.4) | 6.7 (8.5) | 11.4 (14.3) | 79.6 |
| East Cambridgeshire | 7.8 (11.9) | 1.1 (1.7) | 0.0 (0.0) | 2.2 (3.4) | 10.0 (15.3) | 6.7 (10.2) | 6.7 (10.2) | 3.3 (5.1) | 10.0 (15.3) | 17.8 (27.1) | 65.7 |
| East Devon | 6.2 (8.3) | 4.8 (6.4) | 1.4 (1.8) | 0.0 (0.0) | 13.0 (17.4) | 6.8 (9.2) | 15.0 (20.2) | 6.8 (9.2) | 4.8 (6.4) | 15.7 (21.1) | 74.5 |
| East Hampshire | 2.5 (4.2) | 3.3 (5.6) | 1.6 (2.8) | 0.0 (0.0) | 7.4 (12.5) | 7.4 (12.5) | 9.0 (15.3) | 4.9 (8.3) | 7.4 (12.5) | 15.5 (26.4) | 58.9 |
| East Hertfordshire | 3.3 (5.1) | 3.3 (5.1) | 1.3 (2.0) | 2.7 (4.1) | 8.7 (13.3) | 6.0 (9.2) | 10.0 (15.3) | 8.7 (13.3) | 8.7 (13.3) | 12.7 (19.4) | 65.4 |
|  | **Outlets (n (%)) per 100,000 population by cuisine type** | | | | | | | | | | |
| **Local Authority** | **Burger** | **Chicken** | **Desserts** | **Fast food** | **Fish & Chips** | **Kebab** | **Pizza** | **Sand/Caf/Bake** | **S Asian** | **SE & E Asian** | **Overall** |
| East Lindsey | 12.7 (7.7) | 5.6 (3.4) | 12.0 (7.3) | 4.2 (2.6) | 39.5 (24.0) | 14.1 (8.6) | 20.5 (12.4) | 18.3 (11.2) | 11.3 (6.9) | 26.1 (15.9) | 164.4 |
| East Northamptonshire | 3.2 (4.2) | 5.3 (7.0) | 0.0 (0.0) | 2.1 (2.8) | 13.8 (18.3) | 4.2 (5.6) | 10.6 (14.1) | 7.4 (9.9) | 11.6 (15.5) | 16.9 (22.5) | 75.1 |
| East Riding of Yorkshire | 5.6 (6.1) | 1.5 (1.6) | 1.2 (1.3) | 0.9 (1.0) | 23.4 (25.6) | 5.0 (5.4) | 20.2 (22.1) | 8.5 (9.3) | 7.3 (8.0) | 17.9 (19.6) | 91.4 |
| East Staffordshire | 13.4 (11.1) | 10.9 (9.0) | 1.7 (1.4) | 3.3 (2.8) | 20.9 (17.4) | 5.0 (4.2) | 13.4 (11.1) | 15.0 (12.5) | 19.2 (16.0) | 17.5 (14.6) | 120.2 |
| East Suffolk | 4.4 (5.0) | 4.4 (5.0) | 0.0 (0.0) | 2.4 (2.8) | 23.7 (27.1) | 8.4 (9.6) | 9.2 (10.6) | 8.0 (9.2) | 8.4 (9.6) | 18.4 (21.1) | 87.4 |
| Eastbourne | 8.7 (10.1) | 1.9 (2.2) | 1.9 (2.2) | 1.0 (1.1) | 15.4 (18.0) | 10.6 (12.4) | 11.6 (13.5) | 6.7 (7.9) | 7.7 (9.0) | 20.2 (23.6) | 85.8 |
| Eastleigh | 4.5 (6.5) | 1.5 (2.2) | 0.7 (1.1) | 0.7 (1.1) | 12.7 (18.5) | 5.2 (7.6) | 9.0 (13.0) | 6.0 (8.7) | 11.2 (16.3) | 17.2 (25.0) | 68.9 |
| Eden | 11.3 (12.2) | 3.8 (4.1) | 0.0 (0.0) | 0.0 (0.0) | 16.9 (18.4) | 1.9 (2.0) | 15.0 (16.3) | 13.1 (14.3) | 11.3 (12.2) | 18.8 (20.4) | 92.0 |
| Elmbridge | 5.8 (9.1) | 4.4 (6.8) | 0.7 (1.1) | 1.5 (2.3) | 8.8 (13.6) | 5.1 (8.0) | 13.2 (20.5) | 5.8 (9.1) | 5.1 (8.0) | 13.9 (21.6) | 64.3 |
| Enfield | 5.7 (7.4) | 11.1 (14.4) | 0.9 (1.2) | 2.7 (3.5) | 9.3 (12.1) | 10.8 (14.0) | 11.4 (14.8) | 9.0 (11.7) | 6.3 (8.2) | 9.9 (12.8) | 77.0 |
| Epping Forest | 5.3 (9.3) | 3.0 (5.3) | 0.8 (1.3) | 0.8 (1.3) | 9.9 (17.3) | 2.3 (4.0) | 6.8 (12.0) | 6.8 (12.0) | 9.9 (17.3) | 11.4 (20.0) | 57.0 |
| Epsom and Ewell | 7.4 (10.0) | 3.7 (5.0) | 1.2 (1.7) | 2.5 (3.3) | 7.4 (10.0) | 12.4 (16.7) | 11.2 (15.0) | 2.5 (3.3) | 9.9 (13.3) | 16.1 (21.7) | 74.4 |
| Erewash | 6.9 (6.5) | 3.5 (3.2) | 1.7 (1.6) | 1.7 (1.6) | 17.3 (16.1) | 6.1 (5.6) | 17.3 (16.1) | 18.2 (16.9) | 8.7 (8.1) | 26.0 (24.2) | 107.5 |
| Exeter City | 7.6 (8.9) | 6.1 (7.1) | 0.8 (0.9) | 1.5 (1.8) | 9.9 (11.6) | 9.1 (10.7) | 9.9 (11.6) | 12.2 (14.3) | 8.4 (9.8) | 19.8 (23.2) | 85.2 |
| Fareham | 3.4 (7.8) | 2.6 (5.9) | 0.0 (0.0) | 0.9 (2.0) | 9.5 (21.6) | 0.9 (2.0) | 5.2 (11.8) | 4.3 (9.8) | 7.7 (17.6) | 9.5 (21.6) | 43.9 |
| Fenland | 6.9 (7.6) | 4.9 (5.4) | 2.0 (2.2) | 2.0 (2.2) | 15.7 (17.4) | 7.9 (8.7) | 9.8 (10.9) | 8.8 (9.8) | 8.8 (9.8) | 23.6 (26.1) | 90.3 |
| Folkestone and Hythe | 7.1 (6.9) | 8.0 (7.8) | 0.9 (0.9) | 0.9 (0.9) | 19.5 (19.0) | 6.2 (6.0) | 11.5 (11.2) | 8.0 (7.8) | 12.4 (12.1) | 28.3 (27.6) | 102.7 |
| Forest of Dean | 1.2 (1.8) | 4.6 (7.1) | 0.0 (0.0) | 1.2 (1.8) | 16.1 (25.0) | 3.5 (5.4) | 10.4 (16.1) | 3.5 (5.4) | 8.1 (12.5) | 16.1 (25.0) | 64.5 |
| Fylde | 6.2 (4.9) | 3.7 (2.9) | 8.7 (6.9) | 2.5 (2.0) | 19.8 (15.7) | 12.4 (9.8) | 21.0 (16.7) | 27.2 (21.6) | 8.7 (6.9) | 16.1 (12.7) | 126.3 |
| Gateshead | 9.9 (7.2) | 4.0 (2.9) | 2.5 (1.8) | 1.5 (1.1) | 22.3 (16.1) | 6.4 (4.7) | 24.7 (17.9) | 23.3 (16.8) | 17.3 (12.5) | 26.2 (19.0) | 138.1 |
| Gedling | 4.2 (5.4) | 1.7 (2.2) | 0.0 (0.0) | 1.7 (2.2) | 11.9 (15.2) | 8.5 (10.9) | 15.3 (19.6) | 10.2 (13.0) | 10.2 (13.0) | 14.4 (18.5) | 78.0 |
| Gloucester City | 5.4 (6.1) | 13.2 (14.8) | 0.8 (0.9) | 1.5 (1.7) | 11.6 (13.0) | 8.5 (9.6) | 14.7 (16.5) | 8.5 (9.6) | 10.1 (11.3) | 14.7 (16.5) | 89.1 |
| Gosport | 3.5 (4.0) | 4.7 (5.3) | 0.0 (0.0) | 3.5 (4.0) | 21.2 (24.0) | 4.7 (5.3) | 3.5 (4.0) | 9.4 (10.7) | 13.0 (14.7) | 24.8 (28.0) | 88.4 |
| Gravesham | 5.6 (6.1) | 10.3 (11.2) | 0.9 (1.0) | 1.9 (2.0) | 14.0 (15.3) | 9.4 (10.2) | 13.1 (14.3) | 5.6 (6.1) | 13.1 (14.3) | 17.8 (19.4) | 91.6 |
| Great Yarmouth | 19.1 (10.9) | 6.0 (3.4) | 8.1 (4.6) | 13.1 (7.4) | 48.3 (27.4) | 13.1 (7.4) | 10.1 (5.7) | 17.1 (9.7) | 7.0 (4.0) | 34.2 (19.4) | 176.2 |
| Greenwich | 8.0 (9.3) | 11.8 (13.8) | 1.4 (1.6) | 1.4 (1.6) | 9.7 (11.4) | 7.6 (8.9) | 9.4 (11.0) | 10.8 (12.6) | 11.1 (13.0) | 14.2 (16.7) | 85.4 |
| Guildford | 3.4 (5.7) | 4.0 (6.8) | 0.7 (1.1) | 0.0 (0.0) | 5.4 (9.1) | 4.7 (8.0) | 12.1 (20.5) | 8.1 (13.6) | 8.1 (13.6) | 12.8 (21.6) | 59.1 |
| Hackney | 8.5 (9.3) | 11.7 (12.7) | 2.1 (2.3) | 3.2 (3.5) | 8.2 (8.9) | 9.2 (10.0) | 16.4 (17.8) | 10.0 (10.8) | 10.0 (10.8) | 12.8 (13.9) | 92.1 |
| Halton | 4.6 (5.3) | 6.2 (7.0) | 1.5 (1.8) | 1.5 (1.8) | 20.9 (23.7) | 3.1 (3.5) | 17.8 (20.2) | 12.4 (14.0) | 6.2 (7.0) | 13.9 (15.8) | 88.1 |
|  | **Outlets (n (%)) per 100,000 population by cuisine type** | | | | | | | | | | |
| **Local Authority** | **Burger** | **Chicken** | **Desserts** | **Fast food** | **Fish & Chips** | **Kebab** | **Pizza** | **Sand/Caf/Bake** | **S Asian** | **SE & E Asian** | **Overall** |
| Hambleton | 3.3 (4.9) | 1.1 (1.6) | 1.1 (1.6) | 0.0 (0.0) | 17.5 (26.2) | 2.2 (3.3) | 18.6 (27.9) | 12.0 (18.0) | 3.3 (4.9) | 7.6 (11.5) | 66.6 |
| Hammersmith and Fulham | 11.9 (12.4) | 14.0 (14.7) | 3.2 (3.4) | 0.0 (0.0) | 5.9 (6.2) | 6.5 (6.8) | 18.4 (19.2) | 12.4 (13.0) | 11.9 (12.4) | 11.3 (11.9) | 95.6 |
| Harborough | 6.4 (10.3) | 2.1 (3.4) | 1.1 (1.7) | 2.1 (3.4) | 12.8 (20.7) | 1.1 (1.7) | 9.6 (15.5) | 8.5 (13.8) | 5.3 (8.6) | 12.8 (20.7) | 61.8 |
| Haringey | 5.6 (6.6) | 16.8 (19.8) | 0.4 (0.4) | 3.7 (4.4) | 6.7 (7.9) | 10.1 (11.9) | 13.8 (16.3) | 6.7 (7.9) | 8.6 (10.1) | 12.3 (14.5) | 84.5 |
| Harlow | 5.7 (7.1) | 6.9 (8.6) | 0.0 (0.0) | 1.1 (1.4) | 10.3 (12.9) | 8.0 (10.0) | 17.2 (21.4) | 8.0 (10.0) | 5.7 (7.1) | 17.2 (21.4) | 80.4 |
| Harrogate | 5.6 (6.8) | 2.5 (3.0) | 3.1 (3.8) | 0.6 (0.8) | 12.4 (15.2) | 1.9 (2.3) | 19.9 (24.2) | 10.6 (12.9) | 8.1 (9.8) | 17.4 (21.2) | 82.1 |
| Harrow | 6.0 (6.9) | 13.1 (15.2) | 0.8 (0.9) | 3.6 (4.1) | 5.2 (6.0) | 8.4 (9.7) | 12.7 (14.7) | 10.0 (11.5) | 13.5 (15.7) | 13.1 (15.2) | 86.4 |
| Hart | 5.2 (7.0) | 7.2 (9.9) | 1.0 (1.4) | 0.0 (0.0) | 7.2 (9.9) | 11.3 (15.5) | 15.5 (21.1) | 8.2 (11.3) | 10.3 (14.1) | 7.2 (9.9) | 73.1 |
| Hartlepool | 7.5 (4.8) | 5.3 (3.4) | 3.2 (2.0) | 3.2 (2.0) | 23.5 (15.0) | 1.1 (0.7) | 42.7 (27.2) | 32.0 (20.4) | 7.5 (4.8) | 31.0 (19.7) | 156.9 |
| Hastings | 9.7 (7.4) | 9.7 (7.4) | 2.2 (1.7) | 1.1 (0.8) | 27.0 (20.7) | 11.9 (9.1) | 22.7 (17.4) | 6.5 (5.0) | 16.2 (12.4) | 23.7 (18.2) | 130.6 |
| Havant | 10.3 (10.7) | 6.3 (6.6) | 2.4 (2.5) | 3.2 (3.3) | 15.1 (15.6) | 4.8 (4.9) | 7.9 (8.2) | 10.3 (10.7) | 15.1 (15.6) | 21.4 (22.1) | 96.7 |
| Havering | 8.9 (9.2) | 10.4 (10.8) | 1.2 (1.2) | 1.5 (1.6) | 12.7 (13.2) | 7.3 (7.6) | 11.2 (11.6) | 10.0 (10.4) | 16.2 (16.8) | 17.0 (17.6) | 96.3 |
| Herefordshire | 6.2 (10.4) | 4.1 (7.0) | 0.0 (0.0) | 1.6 (2.6) | 13.0 (21.7) | 3.6 (6.1) | 7.3 (12.2) | 9.3 (15.7) | 3.6 (6.1) | 10.9 (18.3) | 59.6 |
| Hertsmere | 6.7 (7.0) | 9.5 (10.0) | 2.9 (3.0) | 1.0 (1.0) | 11.4 (12.0) | 4.8 (5.0) | 19.1 (20.0) | 14.3 (15.0) | 13.3 (14.0) | 12.4 (13.0) | 95.3 |
| High Peak | 4.3 (3.5) | 2.2 (1.8) | 4.3 (3.5) | 0.0 (0.0) | 21.6 (17.7) | 5.4 (4.4) | 24.8 (20.4) | 14.0 (11.5) | 21.6 (17.7) | 23.7 (19.5) | 121.9 |
| Hillingdon | 7.5 (8.7) | 11.7 (13.6) | 1.3 (1.5) | 2.6 (3.0) | 9.8 (11.4) | 7.2 (8.3) | 16.0 (18.6) | 6.8 (8.0) | 9.8 (11.4) | 13.4 (15.5) | 86.0 |
| Hinckley and Bosworth | 9.7 (10.4) | 2.7 (2.8) | 0.0 (0.0) | 0.9 (0.9) | 21.2 (22.6) | 2.7 (2.8) | 12.4 (13.2) | 8.8 (9.4) | 11.5 (12.3) | 23.9 (25.5) | 93.7 |
| Horsham | 4.2 (9.1) | 1.4 (3.0) | 0.7 (1.5) | 0.0 (0.0) | 7.6 (16.7) | 3.5 (7.6) | 7.0 (15.2) | 4.2 (9.1) | 7.6 (16.7) | 9.7 (21.2) | 45.9 |
| Hounslow | 9.9 (12.1) | 10.7 (13.0) | 0.4 (0.4) | 1.5 (1.8) | 6.6 (8.1) | 8.5 (10.3) | 16.2 (19.7) | 8.8 (10.8) | 9.6 (11.7) | 9.9 (12.1) | 82.1 |
| Hull City | 13.1 (8.7) | 8.9 (5.9) | 1.5 (1.0) | 0.8 (0.5) | 29.3 (19.3) | 2.7 (1.8) | 40.8 (27.0) | 18.1 (12.0) | 8.9 (5.9) | 27.3 (18.1) | 151.3 |
| Huntingdonshire | 5.6 (7.0) | 5.1 (6.3) | 1.1 (1.4) | 1.7 (2.1) | 14.6 (18.2) | 4.5 (5.6) | 10.7 (13.3) | 11.8 (14.7) | 11.8 (14.7) | 13.5 (16.8) | 80.4 |
| Hyndburn | 6.2 (4.0) | 9.9 (6.5) | 6.2 (4.0) | 6.2 (4.0) | 24.7 (16.1) | 8.6 (5.6) | 23.4 (15.3) | 21.0 (13.7) | 21.0 (13.7) | 25.9 (16.9) | 153.0 |
| Ipswich | 11.7 (9.0) | 5.8 (4.5) | 2.9 (2.3) | 2.2 (1.7) | 19.7 (15.3) | 14.6 (11.3) | 16.1 (12.4) | 16.8 (13.0) | 15.3 (11.9) | 24.1 (18.6) | 129.3 |
| Isle of Wight | 12.0 (12.4) | 1.4 (1.5) | 2.1 (2.2) | 1.4 (1.5) | 17.6 (18.2) | 5.6 (5.8) | 11.3 (11.7) | 10.6 (10.9) | 14.8 (15.3) | 19.8 (20.4) | 96.6 |
| Isles of Scilly | 0.0 (0.0) | 0.0 (0.0) | 0.0 (0.0) | 0.0 (0.0) | 134.9 (100.0) | 0.0 (0.0) | 0.0 (0.0) | 0.0 (0.0) | 0.0 (0.0) | 0.0 (0.0) | 134.9 |
| Islington | 9.9 (9.4) | 10.3 (9.8) | 1.6 (1.6) | 2.5 (2.4) | 6.6 (6.3) | 13.2 (12.6) | 17.7 (16.9) | 7.8 (7.5) | 15.3 (14.6) | 19.8 (18.9) | 104.8 |
| Kensington and Chelsea | 8.3 (12.4) | 5.1 (7.6) | 3.2 (4.8) | 0.0 (0.0) | 4.5 (6.7) | 4.5 (6.7) | 10.9 (16.2) | 10.9 (16.2) | 9.6 (14.3) | 10.2 (15.2) | 67.3 |
| Kettering | 4.9 (5.3) | 5.9 (6.4) | 2.9 (3.2) | 2.0 (2.1) | 18.7 (20.2) | 4.9 (5.3) | 10.8 (11.7) | 13.8 (14.9) | 8.8 (9.6) | 19.7 (21.3) | 92.4 |
|  | **Outlets (n (%)) per 100,000 population by cuisine type** | | | | | | | | | | |
| **Local Authority** | **Burger** | **Chicken** | **Desserts** | **Fast food** | **Fish & Chips** | **Kebab** | **Pizza** | **Sand/Caf/Bake** | **S Asian** | **SE & E Asian** | **Overall** |
| King's Lynn and West Norfolk | 7.9 (8.5) | 2.6 (2.8) | 1.3 (1.4) | 2.0 (2.1) | 29.7 (31.7) | 7.9 (8.5) | 9.2 (9.9) | 11.2 (12.0) | 6.6 (7.0) | 15.2 (16.2) | 93.8 |
| Kingston-Upon-Thames | 7.9 (10.4) | 8.5 (11.1) | 2.3 (3.0) | 0.0 (0.0) | 7.3 (9.6) | 9.0 (11.9) | 14.6 (19.3) | 9.6 (12.6) | 3.9 (5.2) | 13.0 (17.0) | 76.1 |
| Kirklees | 9.8 (7.0) | 10.5 (7.5) | 2.0 (1.5) | 1.6 (1.1) | 21.6 (15.4) | 7.0 (5.0) | 26.4 (18.8) | 19.8 (14.1) | 21.4 (15.3) | 20.0 (14.3) | 140.1 |
| Knowsley | 4.0 (6.3) | 0.7 (1.1) | 1.3 (2.1) | 0.7 (1.1) | 15.9 (25.3) | 0.7 (1.1) | 8.0 (12.6) | 13.3 (21.1) | 2.7 (4.2) | 15.9 (25.3) | 63.0 |
| Lambeth | 12.3 (11.2) | 19.0 (17.4) | 1.5 (1.4) | 1.8 (1.7) | 7.1 (6.4) | 5.8 (5.3) | 19.3 (17.6) | 10.1 (9.2) | 12.9 (11.8) | 19.6 (17.9) | 109.5 |
| Lancaster City | 6.8 (6.7) | 5.5 (5.4) | 2.1 (2.0) | 0.0 (0.0) | 17.8 (17.4) | 6.8 (6.7) | 17.1 (16.8) | 16.4 (16.1) | 9.6 (9.4) | 19.9 (19.5) | 102.0 |
| Leeds | 10.0 (8.6) | 6.6 (5.7) | 3.8 (3.3) | 0.3 (0.2) | 16.4 (14.1) | 3.9 (3.4) | 24.8 (21.4) | 15.1 (13.1) | 12.7 (11.0) | 22.3 (19.3) | 115.9 |
| Leicester City | 11.9 (8.5) | 11.6 (8.3) | 4.0 (2.8) | 4.2 (3.0) | 17.8 (12.8) | 9.0 (6.5) | 21.7 (15.7) | 16.1 (11.6) | 24.0 (17.3) | 18.6 (13.4) | 138.9 |
| Lewes | 3.9 (6.5) | 2.9 (4.8) | 0.0 (0.0) | 1.0 (1.6) | 13.6 (22.6) | 2.9 (4.8) | 11.6 (19.4) | 5.8 (9.7) | 6.8 (11.3) | 11.6 (19.4) | 60.0 |
| Lewisham | 8.8 (7.9) | 25.8 (23.0) | 1.0 (0.9) | 2.9 (2.6) | 11.4 (10.2) | 10.5 (9.3) | 12.8 (11.4) | 6.9 (6.1) | 10.8 (9.6) | 21.3 (19.0) | 112.1 |
| Lichfield | 1.9 (2.9) | 1.0 (1.4) | 1.0 (1.4) | 1.0 (1.4) | 18.1 (27.5) | 1.0 (1.4) | 8.6 (13.0) | 6.7 (10.1) | 11.5 (17.4) | 15.3 (23.2) | 65.9 |
| Lincoln City | 11.1 (8.3) | 6.0 (4.5) | 2.0 (1.5) | 0.0 (0.0) | 21.1 (15.9) | 4.0 (3.0) | 35.2 (26.5) | 16.1 (12.1) | 13.1 (9.8) | 24.2 (18.2) | 132.9 |
| Liverpool | 10.4 (8.0) | 7.6 (5.8) | 3.8 (2.9) | 1.8 (1.4) | 14.7 (11.2) | 5.0 (3.8) | 23.7 (18.2) | 20.1 (15.4) | 14.1 (10.8) | 29.3 (22.5) | 130.5 |
| Luton | 7.5 (8.0) | 18.3 (19.6) | 2.3 (2.5) | 4.7 (5.0) | 8.9 (9.5) | 7.0 (7.5) | 10.8 (11.6) | 9.9 (10.6) | 13.1 (14.1) | 10.8 (11.6) | 93.4 |
| Maidstone | 7.6 (10.8) | 4.7 (6.7) | 0.6 (0.8) | 2.3 (3.3) | 11.1 (15.8) | 2.9 (4.2) | 9.9 (14.2) | 7.0 (10.0) | 8.7 (12.5) | 15.1 (21.7) | 69.8 |
| Maldon | 6.2 (6.9) | 1.5 (1.7) | 3.1 (3.4) | 0.0 (0.0) | 20.0 (22.4) | 7.7 (8.6) | 10.8 (12.1) | 12.3 (13.8) | 12.3 (13.8) | 15.4 (17.2) | 89.3 |
| Malvern Hills | 7.6 (11.3) | 1.3 (1.9) | 1.3 (1.9) | 3.8 (5.7) | 10.2 (15.1) | 2.5 (3.8) | 11.4 (17.0) | 3.8 (5.7) | 8.9 (13.2) | 16.5 (24.5) | 67.3 |
| Manchester | 11.6 (9.0) | 15.9 (12.3) | 2.7 (2.1) | 1.8 (1.4) | 13.6 (10.5) | 7.1 (5.5) | 20.4 (15.8) | 17.9 (13.9) | 20.3 (15.7) | 17.9 (13.9) | 129.1 |
| Mansfield | 7.3 (6.9) | 6.4 (6.0) | 1.8 (1.7) | 1.8 (1.7) | 18.3 (17.2) | 3.7 (3.4) | 13.7 (12.9) | 16.5 (15.5) | 12.8 (12.1) | 23.8 (22.4) | 106.1 |
| Medway | 5.4 (6.2) | 4.3 (4.9) | 0.0 (0.0) | 2.5 (2.9) | 12.9 (14.8) | 13.6 (15.6) | 11.1 (12.8) | 7.9 (9.1) | 11.5 (13.2) | 17.9 (20.6) | 87.2 |
| Melton | 2.0 (2.3) | 5.9 (6.8) | 0.0 (0.0) | 5.9 (6.8) | 13.7 (15.9) | 2.0 (2.3) | 15.6 (18.2) | 13.7 (15.9) | 13.7 (15.9) | 13.7 (15.9) | 85.9 |
| Mendip | 3.5 (6.0) | 2.6 (4.5) | 0.0 (0.0) | 0.9 (1.5) | 13.8 (23.9) | 4.3 (7.5) | 10.4 (17.9) | 6.9 (11.9) | 2.6 (4.5) | 13.0 (22.4) | 58.0 |
| Merton | 5.8 (6.8) | 16.0 (18.8) | 1.0 (1.1) | 0.5 (0.6) | 8.2 (9.7) | 9.2 (10.8) | 10.7 (12.5) | 10.2 (11.9) | 9.2 (10.8) | 14.5 (17.0) | 85.2 |
| Mid Devon | 3.6 (7.9) | 1.2 (2.6) | 0.0 (0.0) | 0.0 (0.0) | 6.1 (13.2) | 7.3 (15.8) | 8.5 (18.4) | 6.1 (13.2) | 2.4 (5.3) | 10.9 (23.7) | 46.2 |
| Mid Suffolk | 3.9 (6.7) | 1.0 (1.7) | 1.0 (1.7) | 0.0 (0.0) | 19.3 (33.3) | 2.9 (5.0) | 5.8 (10.0) | 6.7 (11.7) | 6.7 (11.7) | 10.6 (18.3) | 57.8 |
| Mid Sussex | 8.6 (13.7) | 2.0 (3.2) | 0.0 (0.0) | 1.3 (2.1) | 11.9 (18.9) | 2.0 (3.2) | 9.9 (15.8) | 6.0 (9.5) | 11.3 (17.9) | 9.9 (15.8) | 62.9 |
| Middlesbrough | 9.2 (7.5) | 7.1 (5.8) | 2.1 (1.7) | 0.7 (0.6) | 14.9 (12.1) | 5.0 (4.0) | 40.4 (32.9) | 13.5 (11.0) | 11.3 (9.2) | 18.4 (15.0) | 122.7 |
| Milton Keynes | 11.1 (10.6) | 10.4 (9.9) | 1.9 (1.8) | 0.7 (0.7) | 12.6 (12.1) | 17.1 (16.3) | 15.6 (14.9) | 10.4 (9.9) | 10.8 (10.3) | 14.1 (13.5) | 104.7 |
| Mole Valley | 0.0 (0.0) | 5.7 (9.8) | 1.1 (2.0) | 1.1 (2.0) | 8.0 (13.7) | 4.6 (7.8) | 14.9 (25.5) | 12.6 (21.6) | 3.4 (5.9) | 6.9 (11.8) | 58.5 |
|  | **Outlets (n (%)) per 100,000 population by cuisine type** | | | | | | | | | | |
| **Local Authority** | **Burger** | **Chicken** | **Desserts** | **Fast food** | **Fish & Chips** | **Kebab** | **Pizza** | **Sand/Caf/Bake** | **S Asian** | **SE & E Asian** | **Overall** |
| New Forest | 7.2 (9.2) | 2.2 (2.8) | 3.3 (4.2) | 2.2 (2.8) | 10.0 (12.7) | 5.0 (6.3) | 7.8 (9.9) | 9.4 (12.0) | 13.9 (17.6) | 17.8 (22.5) | 78.9 |
| Newark and Sherwood | 5.7 (6.6) | 3.3 (3.8) | 0.8 (0.9) | 2.5 (2.8) | 18.8 (21.7) | 4.9 (5.7) | 13.1 (15.1) | 11.4 (13.2) | 7.4 (8.5) | 18.8 (21.7) | 86.6 |
| Newcastle Upon Tyne | 10.6 (7.6) | 7.9 (5.7) | 5.3 (3.8) | 0.7 (0.5) | 12.9 (9.3) | 5.3 (3.8) | 27.4 (19.8) | 31.0 (22.4) | 12.2 (8.8) | 25.1 (18.1) | 138.4 |
| Newcastle-Under-Lyme | 6.2 (6.0) | 3.9 (3.7) | 2.3 (2.2) | 3.1 (3.0) | 16.2 (15.7) | 10.8 (10.4) | 13.1 (12.7) | 13.1 (12.7) | 13.1 (12.7) | 21.6 (20.9) | 103.5 |
| Newham | 8.2 (9.3) | 18.4 (20.8) | 1.7 (1.9) | 4.2 (4.8) | 6.5 (7.3) | 7.6 (8.6) | 12.2 (13.7) | 8.2 (9.3) | 9.9 (11.2) | 11.6 (13.1) | 88.6 |
| North Devon | 7.2 (8.9) | 4.1 (5.1) | 2.1 (2.5) | 1.0 (1.3) | 17.5 (21.5) | 2.1 (2.5) | 11.3 (13.9) | 13.4 (16.5) | 5.1 (6.3) | 17.5 (21.5) | 81.3 |
| North East Derbyshire | 4.9 (6.8) | 2.0 (2.7) | 0.0 (0.0) | 2.0 (2.7) | 12.8 (17.8) | 2.0 (2.7) | 11.8 (16.4) | 4.9 (6.8) | 8.9 (12.3) | 22.7 (31.5) | 71.9 |
| North East Lincolnshire | 10.0 (7.5) | 3.8 (2.8) | 1.9 (1.4) | 1.9 (1.4) | 22.6 (17.0) | 8.1 (6.1) | 29.5 (22.2) | 13.8 (10.4) | 10.0 (7.5) | 31.3 (23.6) | 132.9 |
| North Hertfordshire | 8.2 (10.3) | 3.0 (3.7) | 0.7 (0.9) | 3.7 (4.7) | 11.2 (14.0) | 5.2 (6.5) | 9.7 (12.1) | 8.2 (10.3) | 15.7 (19.6) | 14.2 (17.8) | 80.1 |
| North Kesteven | 6.0 (8.1) | 1.7 (2.3) | 0.9 (1.2) | 0.9 (1.2) | 15.4 (20.9) | 3.4 (4.7) | 12.8 (17.4) | 7.7 (10.5) | 8.6 (11.6) | 16.3 (22.1) | 73.6 |
| North Lincolnshire | 7.5 (7.6) | 4.1 (4.1) | 3.5 (3.5) | 0.6 (0.6) | 18.0 (18.1) | 5.2 (5.3) | 20.3 (20.5) | 9.3 (9.4) | 9.3 (9.4) | 21.5 (21.6) | 99.3 |
| North Norfolk | 9.5 (9.3) | 2.9 (2.8) | 4.8 (4.6) | 1.9 (1.9) | 26.7 (25.9) | 6.7 (6.5) | 6.7 (6.5) | 14.3 (13.9) | 11.4 (11.1) | 18.1 (17.6) | 103.0 |
| North Somerset | 5.1 (7.5) | 2.8 (4.1) | 0.9 (1.4) | 2.8 (4.1) | 15.3 (22.4) | 5.1 (7.5) | 9.3 (13.6) | 6.5 (9.5) | 7.4 (10.9) | 13.0 (19.0) | 68.4 |
| North Tyneside | 5.3 (5.2) | 3.4 (3.3) | 1.0 (0.9) | 0.5 (0.5) | 16.8 (16.5) | 3.4 (3.3) | 16.8 (16.5) | 16.8 (16.5) | 12.5 (12.3) | 25.5 (25.0) | 102.0 |
| North Warwickshire | 13.8 (12.2) | 6.1 (5.4) | 0.0 (0.0) | 1.5 (1.4) | 27.6 (24.3) | 0.0 (0.0) | 15.3 (13.5) | 12.3 (10.8) | 19.9 (17.6) | 16.9 (14.9) | 113.4 |
| North West Leicestershire | 7.7 (9.8) | 2.9 (3.7) | 0.0 (0.0) | 0.0 (0.0) | 21.2 (26.8) | 2.9 (3.7) | 11.6 (14.6) | 11.6 (14.6) | 4.8 (6.1) | 16.4 (20.7) | 79.1 |
| Northampton | 7.1 (7.5) | 10.7 (11.3) | 0.4 (0.5) | 2.2 (2.4) | 11.6 (12.3) | 5.8 (6.1) | 12.9 (13.7) | 9.8 (10.4) | 10.7 (11.3) | 23.2 (24.5) | 94.4 |
| Northumberland | 5.6 (5.4) | 3.4 (3.3) | 4.3 (4.2) | 2.2 (2.1) | 17.1 (16.6) | 1.9 (1.8) | 19.8 (19.3) | 14.3 (13.9) | 13.0 (12.7) | 21.1 (20.5) | 102.7 |
| Norwich City | 14.9 (9.1) | 7.8 (4.8) | 4.3 (2.6) | 5.7 (3.5) | 27.7 (16.9) | 8.5 (5.2) | 29.2 (17.7) | 22.1 (13.4) | 14.2 (8.7) | 29.9 (18.2) | 164.3 |
| Nottingham City | 9.0 (7.2) | 12.9 (10.3) | 3.6 (2.9) | 3.3 (2.6) | 14.4 (11.5) | 6.6 (5.3) | 18.3 (14.6) | 18.3 (14.6) | 15.3 (12.2) | 23.4 (18.7) | 125.3 |
| Nuneaton and Bedworth | 8.5 (9.8) | 4.6 (5.4) | 0.0 (0.0) | 0.0 (0.0) | 15.4 (17.9) | 1.5 (1.8) | 14.6 (17.0) | 13.9 (16.1) | 11.5 (13.4) | 16.2 (18.7) | 86.2 |
| Oadby and Wigston | 3.5 (4.3) | 1.8 (2.1) | 0.0 (0.0) | 3.5 (4.3) | 12.3 (14.9) | 3.5 (4.3) | 17.5 (21.3) | 12.3 (14.9) | 7.0 (8.5) | 21.0 (25.5) | 82.4 |
| Oldham | 4.6 (3.9) | 11.0 (9.3) | 2.1 (1.8) | 1.3 (1.1) | 16.9 (14.3) | 3.0 (2.5) | 11.8 (10.0) | 17.3 (14.6) | 26.6 (22.5) | 23.6 (20.0) | 118.1 |
| Oxford City | 9.2 (10.9) | 7.9 (9.3) | 1.3 (1.6) | 0.7 (0.8) | 4.6 (5.4) | 13.8 (16.3) | 15.7 (18.6) | 12.5 (14.7) | 7.2 (8.5) | 11.8 (14.0) | 84.6 |
| Pendle | 2.2 (1.9) | 11.9 (10.6) | 1.1 (1.0) | 2.2 (1.9) | 26.1 (23.1) | 4.3 (3.8) | 14.1 (12.5) | 9.8 (8.7) | 15.2 (13.5) | 26.1 (23.1) | 112.9 |
| Peterborough City | 8.9 (8.7) | 7.9 (7.7) | 2.5 (2.4) | 3.0 (2.9) | 15.3 (14.9) | 4.4 (4.3) | 15.8 (15.4) | 11.4 (11.1) | 11.9 (11.5) | 21.8 (21.2) | 102.8 |
| Plymouth City | 7.2 (8.6) | 5.3 (6.3) | 0.4 (0.5) | 3.4 (4.1) | 11.8 (14.0) | 4.6 (5.4) | 10.7 (12.7) | 8.0 (9.5) | 11.4 (13.6) | 21.4 (25.3) | 84.3 |
| Portsmouth | 10.7 (8.4) | 4.2 (3.3) | 2.3 (1.8) | 3.3 (2.6) | 18.1 (14.2) | 12.1 (9.5) | 11.2 (8.8) | 22.3 (17.5) | 16.8 (13.1) | 26.5 (20.8) | 127.5 |
| Preston | 23.8 (15.2) | 11.9 (7.6) | 3.5 (2.2) | 1.4 (0.9) | 19.6 (12.6) | 7.7 (4.9) | 23.1 (14.8) | 21.7 (13.9) | 23.1 (14.8) | 20.3 (13.0) | 155.8 |
|  | **Outlets (n (%)) per 100,000 population by cuisine type** | | | | | | | | | | |
| **Local Authority** | **Burger** | **Chicken** | **Desserts** | **Fast food** | **Fish & Chips** | **Kebab** | **Pizza** | **Sand/Caf/Bake** | **S Asian** | **SE & E Asian** | **Overall** |
| Reading | 10.5 (8.8) | 9.3 (7.8) | 0.6 (0.5) | 1.2 (1.0) | 10.5 (8.8) | 11.7 (9.8) | 19.8 (16.6) | 19.2 (16.1) | 12.4 (10.4) | 24.1 (20.2) | 119.3 |
| Redbridge | 4.9 (7.3) | 10.2 (15.0) | 1.0 (1.5) | 3.9 (5.8) | 7.2 (10.7) | 6.9 (10.2) | 8.8 (13.1) | 6.6 (9.7) | 8.8 (13.1) | 9.2 (13.6) | 67.5 |
| Redcar and Cleveland | 5.8 (4.9) | 1.5 (1.2) | 3.6 (3.1) | 0.7 (0.6) | 24.8 (21.0) | 2.9 (2.5) | 36.5 (30.9) | 13.1 (11.1) | 7.3 (6.2) | 21.9 (18.5) | 118.1 |
| Redditch | 9.4 (10.4) | 4.7 (5.2) | 3.5 (3.9) | 1.2 (1.3) | 15.2 (16.9) | 2.3 (2.6) | 15.2 (16.9) | 7.0 (7.8) | 14.1 (15.6) | 17.6 (19.5) | 90.3 |
| Reigate and Banstead | 6.7 (11.8) | 4.0 (7.1) | 0.0 (0.0) | 0.0 (0.0) | 7.4 (12.9) | 6.1 (10.6) | 8.1 (14.1) | 6.1 (10.6) | 8.1 (14.1) | 10.8 (18.8) | 57.1 |
| Ribble Valley | 1.6 (1.8) | 4.9 (5.3) | 0.0 (0.0) | 1.6 (1.8) | 16.4 (17.5) | 1.6 (1.8) | 18.1 (19.3) | 16.4 (17.5) | 16.4 (17.5) | 16.4 (17.5) | 93.6 |
| Richmondshire | 7.4 (8.3) | 1.9 (2.1) | 1.9 (2.1) | 0.0 (0.0) | 22.3 (25.0) | 0.0 (0.0) | 16.8 (18.8) | 14.9 (16.7) | 11.2 (12.5) | 13.0 (14.6) | 89.3 |
| Richmond-Upon-Thames | 6.1 (9.6) | 2.0 (3.2) | 0.0 (0.0) | 0.5 (0.8) | 10.1 (16.0) | 3.5 (5.6) | 14.6 (23.2) | 8.1 (12.8) | 7.1 (11.2) | 11.1 (17.6) | 63.1 |
| Rochdale | 5.8 (4.2) | 12.1 (8.7) | 3.6 (2.6) | 0.0 (0.0) | 23.8 (17.2) | 9.9 (7.1) | 18.0 (12.9) | 21.1 (15.2) | 22.0 (15.9) | 22.5 (16.2) | 138.9 |
| Rochford | 3.4 (5.8) | 5.7 (9.6) | 0.0 (0.0) | 0.0 (0.0) | 5.7 (9.6) | 4.6 (7.7) | 11.4 (19.2) | 5.7 (9.6) | 6.9 (11.5) | 16.0 (26.9) | 59.5 |
| Rossendale | 5.6 (4.8) | 2.8 (2.4) | 1.4 (1.2) | 2.8 (2.4) | 29.4 (25.3) | 2.8 (2.4) | 19.6 (16.9) | 7.0 (6.0) | 23.8 (20.5) | 21.0 (18.1) | 116.1 |
| Rother | 1.0 (2.0) | 3.1 (6.1) | 0.0 (0.0) | 0.0 (0.0) | 10.4 (20.4) | 7.3 (14.3) | 4.2 (8.2) | 3.1 (6.1) | 7.3 (14.3) | 14.6 (28.6) | 51.0 |
| Rotherham | 10.5 (9.0) | 7.2 (6.1) | 3.0 (2.6) | 1.5 (1.3) | 20.7 (17.7) | 4.9 (4.2) | 21.9 (18.7) | 13.6 (11.6) | 12.1 (10.3) | 21.5 (18.4) | 116.8 |
| Rugby | 3.7 (4.3) | 3.7 (4.3) | 0.9 (1.1) | 0.9 (1.1) | 16.5 (19.4) | 3.7 (4.3) | 13.8 (16.1) | 11.0 (12.9) | 11.0 (12.9) | 20.2 (23.7) | 85.4 |
| Runnymede | 5.6 (7.9) | 4.5 (6.3) | 0.0 (0.0) | 0.0 (0.0) | 10.1 (14.3) | 6.7 (9.5) | 15.7 (22.2) | 8.9 (12.7) | 7.8 (11.1) | 11.2 (15.9) | 70.5 |
| Rushcliffe | 4.2 (6.7) | 0.8 (1.3) | 0.0 (0.0) | 0.8 (1.3) | 11.7 (18.7) | 0.0 (0.0) | 9.2 (14.7) | 10.9 (17.3) | 10.1 (16.0) | 15.1 (24.0) | 62.9 |
| Rushmoor | 9.5 (9.9) | 7.4 (7.7) | 0.0 (0.0) | 6.3 (6.6) | 11.6 (12.1) | 8.5 (8.8) | 18.0 (18.7) | 11.6 (12.1) | 9.5 (9.9) | 13.7 (14.3) | 96.2 |
| Rutland | 2.5 (3.7) | 2.5 (3.7) | 0.0 (0.0) | 0.0 (0.0) | 15.0 (22.2) | 2.5 (3.7) | 7.5 (11.1) | 5.0 (7.4) | 10.0 (14.8) | 22.5 (33.3) | 67.6 |
| Ryedale | 9.0 (8.9) | 3.6 (3.6) | 3.6 (3.6) | 3.6 (3.6) | 21.7 (21.4) | 0.0 (0.0) | 19.9 (19.6) | 19.9 (19.6) | 3.6 (3.6) | 16.3 (16.1) | 101.1 |
| Salford | 10.0 (7.8) | 8.9 (6.9) | 2.7 (2.1) | 0.8 (0.6) | 17.8 (13.8) | 5.4 (4.2) | 22.0 (17.1) | 19.7 (15.3) | 16.2 (12.6) | 25.5 (19.8) | 129.0 |
| Sandwell | 7.3 (5.9) | 9.1 (7.4) | 1.8 (1.5) | 1.5 (1.2) | 29.8 (24.2) | 6.4 (5.2) | 21.9 (17.8) | 10.7 (8.6) | 11.9 (9.6) | 22.8 (18.5) | 123.3 |
| Scarborough | 12.9 (8.3) | 2.8 (1.8) | 7.4 (4.7) | 1.8 (1.2) | 45.1 (29.0) | 5.5 (3.6) | 32.2 (20.7) | 21.1 (13.6) | 6.4 (4.1) | 20.2 (13.0) | 155.4 |
| Sedgemoor | 5.7 (6.5) | 5.7 (6.5) | 1.6 (1.9) | 1.6 (1.9) | 21.1 (24.3) | 9.7 (11.2) | 8.9 (10.3) | 8.1 (9.3) | 6.5 (7.5) | 17.9 (20.6) | 86.9 |
| Sefton | 7.6 (7.5) | 3.6 (3.6) | 1.8 (1.8) | 1.8 (1.8) | 15.9 (15.8) | 5.1 (5.0) | 18.8 (18.6) | 12.3 (12.2) | 10.9 (10.8) | 23.2 (22.9) | 100.9 |
| Selby | 4.4 (5.0) | 4.4 (5.0) | 3.3 (3.7) | 0.0 (0.0) | 15.4 (17.5) | 2.2 (2.5) | 17.7 (20.0) | 11.0 (12.5) | 12.1 (13.7) | 17.7 (20.0) | 88.3 |
| Sevenoaks | 3.3 (7.5) | 0.8 (1.9) | 0.0 (0.0) | 1.7 (3.8) | 6.6 (15.1) | 5.8 (13.2) | 9.1 (20.8) | 4.1 (9.4) | 6.6 (15.1) | 5.8 (13.2) | 43.9 |
| Sheffield | 7.7 (6.7) | 6.2 (5.3) | 1.0 (0.9) | 1.5 (1.3) | 16.4 (14.2) | 6.3 (5.5) | 21.7 (18.8) | 18.0 (15.6) | 13.2 (11.4) | 23.3 (20.2) | 115.2 |
| Shropshire | 4.3 (6.1) | 1.9 (2.6) | 0.3 (0.4) | 3.1 (4.4) | 17.0 (24.0) | 4.0 (5.7) | 8.7 (12.2) | 9.9 (14.0) | 6.2 (8.7) | 15.5 (21.8) | 70.9 |
| Slough | 8.7 (9.8) | 16.0 (18.2) | 0.0 (0.0) | 1.3 (1.5) | 8.7 (9.8) | 8.7 (9.8) | 16.7 (18.9) | 6.7 (7.6) | 8.7 (9.8) | 12.7 (14.4) | 88.3 |
|  | **Outlets (n (%)) per 100,000 population by cuisine type** | | | | | | | | | | |
| **Local Authority** | **Burger** | **Chicken** | **Desserts** | **Fast food** | **Fish & Chips** | **Kebab** | **Pizza** | **Sand/Caf/Bake** | **S Asian** | **SE & E Asian** | **Overall** |
| Solihull | 4.2 (5.7) | 4.2 (5.7) | 0.5 (0.6) | 0.5 (0.6) | 12.5 (17.2) | 2.8 (3.8) | 11.6 (15.9) | 12.9 (17.8) | 11.1 (15.3) | 12.5 (17.2) | 72.6 |
| Somerset West and Taunton | 7.1 (10.1) | 3.2 (4.6) | 0.0 (0.0) | 1.9 (2.8) | 16.8 (23.9) | 3.9 (5.5) | 11.0 (15.6) | 8.4 (11.9) | 1.9 (2.8) | 16.1 (22.9) | 70.3 |
| South Buckinghamshire | 7.1 (10.6) | 4.3 (6.4) | 0.0 (0.0) | 2.9 (4.3) | 7.1 (10.6) | 1.4 (2.1) | 8.6 (12.8) | 11.4 (17.0) | 15.7 (23.4) | 8.6 (12.8) | 67.1 |
| South Cambridgeshire | 4.4 (10.4) | 1.3 (3.0) | 0.0 (0.0) | 0.6 (1.5) | 5.7 (13.4) | 5.0 (11.9) | 7.5 (17.9) | 1.9 (4.5) | 6.9 (16.4) | 8.8 (20.9) | 42.1 |
| South Derbyshire | 1.9 (2.8) | 6.5 (9.7) | 0.0 (0.0) | 0.0 (0.0) | 17.7 (26.4) | 0.9 (1.4) | 7.5 (11.1) | 8.4 (12.5) | 8.4 (12.5) | 15.8 (23.6) | 67.1 |
| South Gloucestershire | 6.3 (10.2) | 3.5 (5.7) | 0.0 (0.0) | 3.2 (5.1) | 12.3 (19.9) | 4.9 (8.0) | 9.1 (14.8) | 6.3 (10.2) | 6.7 (10.8) | 9.5 (15.3) | 61.7 |
| South Hams | 4.6 (8.0) | 0.0 (0.0) | 3.4 (6.0) | 0.0 (0.0) | 9.2 (16.0) | 4.6 (8.0) | 8.0 (14.0) | 9.2 (16.0) | 6.9 (12.0) | 11.5 (20.0) | 57.5 |
| South Holland | 7.4 (8.6) | 3.2 (3.7) | 0.0 (0.0) | 4.2 (4.9) | 17.9 (21.0) | 4.2 (4.9) | 10.5 (12.3) | 7.4 (8.6) | 9.5 (11.1) | 21.0 (24.7) | 85.2 |
| South Kesteven | 6.3 (8.0) | 4.2 (5.3) | 0.0 (0.0) | 1.4 (1.8) | 14.7 (18.6) | 4.9 (6.2) | 14.0 (17.7) | 7.0 (8.8) | 8.4 (10.6) | 18.3 (23.0) | 79.3 |
| South Lakeland | 3.8 (3.5) | 7.6 (7.0) | 6.7 (6.1) | 1.0 (0.9) | 24.7 (22.6) | 2.9 (2.6) | 15.2 (13.9) | 15.2 (13.9) | 13.3 (12.2) | 19.0 (17.4) | 109.4 |
| South Norfolk | 3.5 (6.0) | 0.7 (1.2) | 0.0 (0.0) | 2.8 (4.8) | 14.2 (23.8) | 3.5 (6.0) | 8.5 (14.3) | 7.8 (13.1) | 4.3 (7.1) | 14.2 (23.8) | 59.6 |
| South Northamptonshire | 4.2 (8.3) | 0.0 (0.0) | 2.1 (4.2) | 0.0 (0.0) | 10.6 (20.8) | 3.2 (6.3) | 8.5 (16.7) | 6.3 (12.5) | 4.2 (8.3) | 11.6 (22.9) | 50.8 |
| South Oxfordshire | 4.2 (7.2) | 2.1 (3.6) | 0.0 (0.0) | 0.0 (0.0) | 10.6 (18.1) | 5.6 (9.6) | 9.2 (15.7) | 6.3 (10.8) | 6.3 (10.8) | 14.1 (24.1) | 58.4 |
| South Ribble | 7.2 (7.1) | 9.0 (8.9) | 1.8 (1.8) | 0.9 (0.9) | 18.1 (17.9) | 3.6 (3.6) | 12.6 (12.5) | 10.8 (10.7) | 18.1 (17.9) | 19.0 (18.7) | 101.1 |
| South Somerset | 8.9 (12.2) | 4.2 (5.7) | 0.6 (0.8) | 3.0 (4.1) | 10.1 (13.8) | 4.8 (6.5) | 7.1 (9.8) | 10.7 (14.6) | 6.5 (8.9) | 17.2 (23.6) | 73.1 |
| South Staffordshire | 3.6 (7.1) | 0.9 (1.8) | 0.9 (1.8) | 0.0 (0.0) | 14.2 (28.6) | 1.8 (3.6) | 5.3 (10.7) | 6.2 (12.5) | 4.4 (8.9) | 12.5 (25.0) | 49.8 |
| South Tyneside | 5.3 (4.7) | 7.9 (7.1) | 0.7 (0.6) | 0.0 (0.0) | 16.6 (14.8) | 2.6 (2.4) | 19.2 (17.2) | 15.9 (14.2) | 23.8 (21.3) | 19.9 (17.8) | 111.9 |
| Southampton | 9.1 (8.8) | 6.3 (6.1) | 1.2 (1.1) | 3.6 (3.4) | 13.5 (13.0) | 10.3 (9.9) | 16.6 (16.0) | 8.3 (8.0) | 12.7 (12.2) | 22.2 (21.4) | 103.8 |
| Southend-On-Sea | 8.7 (8.1) | 8.7 (8.1) | 2.2 (2.0) | 1.1 (1.0) | 19.1 (17.8) | 7.1 (6.6) | 10.9 (10.2) | 10.4 (9.6) | 11.5 (10.7) | 27.8 (25.9) | 107.6 |
| Southwark | 10.4 (8.1) | 17.6 (13.8) | 3.8 (2.9) | 0.6 (0.5) | 11.0 (8.6) | 8.5 (6.6) | 19.1 (15.0) | 16.0 (12.5) | 15.4 (12.0) | 25.4 (19.9) | 127.7 |
| Spelthorne | 9.0 (12.5) | 5.0 (6.9) | 1.0 (1.4) | 1.0 (1.4) | 8.0 (11.1) | 3.0 (4.2) | 16.0 (22.2) | 7.0 (9.7) | 7.0 (9.7) | 15.0 (20.8) | 72.1 |
| St Albans City | 2.7 (4.3) | 4.7 (7.5) | 1.3 (2.2) | 0.7 (1.1) | 9.4 (15.1) | 5.4 (8.6) | 10.8 (17.2) | 6.1 (9.7) | 8.8 (14.0) | 12.8 (20.4) | 62.6 |
| St Helens | 5.5 (5.5) | 2.8 (2.7) | 1.7 (1.6) | 0.6 (0.5) | 21.6 (21.4) | 1.7 (1.6) | 18.8 (18.7) | 8.9 (8.8) | 16.1 (15.9) | 23.3 (23.1) | 100.8 |
| Stafford | 2.2 (2.7) | 2.2 (2.7) | 0.7 (0.9) | 2.2 (2.7) | 14.6 (18.2) | 2.2 (2.7) | 15.3 (19.1) | 10.2 (12.7) | 12.4 (15.5) | 18.2 (22.7) | 80.1 |
| Staffordshire Moorlands | 10.2 (10.8) | 3.0 (3.2) | 7.1 (7.5) | 3.0 (3.2) | 17.3 (18.3) | 4.1 (4.3) | 13.2 (14.0) | 10.2 (10.8) | 7.1 (7.5) | 19.3 (20.4) | 94.5 |
| Stevenage | 8.0 (6.4) | 11.4 (9.2) | 6.8 (5.5) | 8.0 (6.4) | 15.9 (12.8) | 1.1 (0.9) | 13.7 (11.0) | 18.2 (14.7) | 17.1 (13.8) | 23.9 (19.3) | 124.1 |
| Stockport | 8.2 (6.3) | 6.5 (4.9) | 3.7 (2.9) | 1.4 (1.0) | 14.7 (11.2) | 5.8 (4.4) | 23.2 (17.7) | 16.4 (12.5) | 23.2 (17.7) | 27.9 (21.4) | 130.9 |
| Stockton On Tees | 7.6 (7.3) | 4.6 (4.4) | 0.0 (0.0) | 0.5 (0.5) | 13.2 (12.7) | 2.5 (2.4) | 32.4 (31.2) | 15.7 (15.1) | 10.1 (9.8) | 17.2 (16.6) | 103.9 |
| Stoke-On-Trent | 8.2 (6.9) | 7.4 (6.2) | 1.6 (1.3) | 3.5 (2.9) | 20.3 (17.0) | 8.6 (7.2) | 11.3 (9.5) | 20.7 (17.3) | 11.7 (9.8) | 26.1 (21.9) | 119.4 |
|  | **Outlets (n (%)) per 100,000 population by cuisine type** | | | | | | | | | | |
| **Local Authority** | **Burger** | **Chicken** | **Desserts** | **Fast food** | **Fish & Chips** | **Kebab** | **Pizza** | **Sand/Caf/Bake** | **S Asian** | **SE & E Asian** | **Overall** |
| Stratford-on-Avon | 5.4 (8.3) | 2.3 (3.6) | 0.8 (1.2) | 0.8 (1.2) | 14.6 (22.6) | 4.6 (7.1) | 6.9 (10.7) | 8.5 (13.1) | 9.2 (14.3) | 11.5 (17.9) | 64.6 |
| Stroud | 4.2 (5.5) | 2.5 (3.3) | 1.7 (2.2) | 3.3 (4.4) | 11.7 (15.4) | 4.2 (5.5) | 12.5 (16.5) | 13.3 (17.6) | 9.2 (12.1) | 13.3 (17.6) | 75.9 |
| Sunderland | 7.2 (5.3) | 3.6 (2.7) | 1.4 (1.1) | 0.4 (0.3) | 19.8 (14.7) | 2.9 (2.1) | 31.3 (23.3) | 25.9 (19.3) | 18.0 (13.4) | 24.1 (17.9) | 134.7 |
| Surrey Heath | 7.8 (10.3) | 3.4 (4.4) | 1.1 (1.5) | 0.0 (0.0) | 11.2 (14.7) | 2.2 (2.9) | 17.9 (23.5) | 6.7 (8.8) | 9.0 (11.8) | 16.8 (22.1) | 76.1 |
| Sutton | 5.8 (5.9) | 14.5 (14.6) | 0.0 (0.0) | 2.9 (2.9) | 16.0 (16.1) | 10.2 (10.2) | 12.6 (12.7) | 6.8 (6.8) | 10.7 (10.7) | 19.9 (20.0) | 99.3 |
| Swale | 6.7 (8.0) | 5.3 (6.4) | 0.0 (0.0) | 2.0 (2.4) | 13.3 (16.0) | 6.0 (7.2) | 10.7 (12.8) | 8.0 (9.6) | 15.3 (18.4) | 16.0 (19.2) | 83.3 |
| Swindon | 5.4 (7.3) | 7.2 (9.8) | 0.0 (0.0) | 3.6 (4.9) | 11.7 (15.9) | 9.5 (12.8) | 9.0 (12.2) | 8.1 (11.0) | 8.1 (11.0) | 11.3 (15.2) | 73.8 |
| Tameside | 8.4 (5.7) | 9.7 (6.6) | 4.0 (2.7) | 2.2 (1.5) | 17.2 (11.7) | 6.6 (4.5) | 16.8 (11.4) | 16.8 (11.4) | 27.8 (18.9) | 38.0 (25.7) | 147.5 |
| Tamworth | 3.9 (4.5) | 1.3 (1.5) | 0.0 (0.0) | 1.3 (1.5) | 18.3 (20.9) | 0.0 (0.0) | 14.3 (16.4) | 11.7 (13.4) | 14.3 (16.4) | 22.2 (25.4) | 87.4 |
| Tandridge | 6.8 (12.0) | 3.4 (6.0) | 1.1 (2.0) | 1.1 (2.0) | 10.2 (18.0) | 5.7 (10.0) | 7.9 (14.0) | 6.8 (12.0) | 4.5 (8.0) | 9.1 (16.0) | 56.7 |
| Teignbridge | 5.2 (6.1) | 1.5 (1.7) | 0.7 (0.9) | 1.5 (1.7) | 20.9 (24.3) | 3.7 (4.3) | 11.9 (13.9) | 9.7 (11.3) | 7.5 (8.7) | 23.1 (27.0) | 85.7 |
| Telford and Wrekin Council | 2.8 (3.4) | 5.6 (6.8) | 0.6 (0.7) | 1.7 (2.0) | 16.7 (20.3) | 5.0 (6.1) | 10.0 (12.2) | 13.3 (16.2) | 9.5 (11.5) | 17.2 (20.9) | 82.3 |
| Tendring | 12.3 (12.7) | 4.1 (4.2) | 2.7 (2.8) | 2.0 (2.1) | 24.6 (25.4) | 4.8 (4.9) | 8.9 (9.2) | 7.5 (7.7) | 7.5 (7.7) | 22.5 (23.2) | 96.9 |
| Test Valley | 5.5 (10.6) | 4.8 (9.1) | 0.0 (0.0) | 0.8 (1.5) | 6.3 (12.1) | 0.8 (1.5) | 8.7 (16.7) | 11.1 (21.2) | 4.0 (7.6) | 10.3 (19.7) | 52.3 |
| Tewkesbury | 3.2 (6.5) | 2.1 (4.3) | 0.0 (0.0) | 1.1 (2.2) | 7.4 (15.2) | 5.3 (10.9) | 6.3 (13.0) | 10.5 (21.7) | 4.2 (8.7) | 8.4 (17.4) | 48.4 |
| Thanet | 7.0 (6.0) | 7.0 (6.0) | 2.1 (1.8) | 5.6 (4.8) | 25.4 (21.6) | 10.6 (9.0) | 15.5 (13.2) | 9.9 (8.4) | 6.3 (5.4) | 28.2 (24.0) | 117.7 |
| Three Rivers | 3.2 (5.8) | 4.3 (7.7) | 0.0 (0.0) | 2.1 (3.8) | 6.4 (11.5) | 4.3 (7.7) | 11.8 (21.2) | 3.2 (5.8) | 8.6 (15.4) | 11.8 (21.2) | 55.7 |
| Thurrock | 13.2 (13.1) | 7.5 (7.4) | 2.3 (2.3) | 1.1 (1.1) | 10.3 (10.2) | 10.9 (10.8) | 13.8 (13.6) | 10.9 (10.8) | 16.1 (15.9) | 14.9 (14.8) | 101.0 |
| Tonbridge and Malling | 4.5 (7.5) | 3.0 (5.0) | 0.0 (0.0) | 0.8 (1.3) | 9.1 (15.0) | 6.8 (11.3) | 9.1 (15.0) | 5.3 (8.8) | 10.6 (17.5) | 11.4 (18.8) | 60.5 |
| Torbay | 8.1 (7.9) | 2.2 (2.2) | 3.7 (3.6) | 2.2 (2.2) | 16.1 (15.8) | 4.4 (4.3) | 8.8 (8.6) | 16.1 (15.8) | 8.8 (8.6) | 31.6 (30.9) | 102.0 |
| Torridge | 8.8 (10.7) | 2.9 (3.6) | 1.5 (1.8) | 2.9 (3.6) | 16.1 (19.6) | 2.9 (3.6) | 10.3 (12.5) | 11.7 (14.3) | 7.3 (8.9) | 17.6 (21.4) | 82.0 |
| Tower Hamlets | 11.4 (9.3) | 13.5 (11.1) | 2.8 (2.3) | 1.2 (1.0) | 4.9 (4.0) | 9.2 (7.6) | 19.4 (15.9) | 15.4 (12.6) | 18.5 (15.2) | 25.6 (21.0) | 121.9 |
| Trafford | 8.8 (7.0) | 8.8 (7.0) | 3.8 (3.0) | 0.8 (0.7) | 13.9 (11.0) | 6.7 (5.4) | 16.9 (13.4) | 14.3 (11.4) | 19.4 (15.4) | 32.4 (25.8) | 126.0 |
| Tunbridge Wells | 4.2 (6.2) | 3.4 (5.0) | 0.0 (0.0) | 3.4 (5.0) | 9.3 (13.8) | 3.4 (5.0) | 13.5 (20.0) | 10.1 (15.0) | 10.9 (16.2) | 9.3 (13.8) | 67.4 |
| Uttlesford | 11.0 (17.5) | 1.1 (1.8) | 0.0 (0.0) | 0.0 (0.0) | 12.1 (19.3) | 4.4 (7.0) | 12.1 (19.3) | 4.4 (7.0) | 11.0 (17.5) | 6.6 (10.5) | 62.4 |
| Vale of White Horse | 3.7 (7.4) | 1.5 (2.9) | 0.0 (0.0) | 0.0 (0.0) | 7.4 (14.7) | 7.4 (14.7) | 7.4 (14.7) | 7.4 (14.7) | 5.1 (10.3) | 10.3 (20.6) | 50.0 |
| Wakefield | 12.9 (8.7) | 5.2 (3.5) | 4.0 (2.7) | 2.6 (1.7) | 27.0 (18.2) | 4.0 (2.7) | 25.6 (17.2) | 22.1 (14.9) | 18.7 (12.6) | 26.4 (17.8) | 148.4 |
| Walsall | 4.9 (4.5) | 6.0 (5.5) | 1.4 (1.3) | 0.7 (0.6) | 29.8 (27.5) | 2.1 (1.9) | 15.8 (14.6) | 12.6 (11.7) | 12.6 (11.7) | 22.4 (20.7) | 108.2 |
| Waltham Forest | 7.6 (7.9) | 20.2 (21.0) | 1.1 (1.1) | 2.2 (2.2) | 8.3 (8.6) | 13.0 (13.5) | 13.4 (13.9) | 9.4 (9.7) | 8.7 (9.0) | 12.6 (13.1) | 96.4 |
|  | **Outlets (n (%)) per 100,000 population by cuisine type** | | | | | | | | | | |
| **Local Authority** | **Burger** | **Chicken** | **Desserts** | **Fast food** | **Fish & Chips** | **Kebab** | **Pizza** | **Sand/Caf/Bake** | **S Asian** | **SE & E Asian** | **Overall** |
| Wandsworth | 7.3 (8.6) | 10.3 (12.2) | 0.9 (1.1) | 3.0 (3.6) | 7.0 (8.3) | 7.3 (8.6) | 17.9 (21.2) | 8.2 (9.7) | 8.8 (10.4) | 13.6 (16.2) | 84.3 |
| Warrington | 8.1 (7.8) | 7.1 (6.8) | 1.0 (0.9) | 2.4 (2.3) | 12.9 (12.3) | 8.1 (7.8) | 11.4 (11.0) | 20.5 (19.6) | 12.4 (11.9) | 20.5 (19.6) | 104.3 |
| Warwick | 6.3 (7.9) | 1.4 (1.8) | 1.4 (1.8) | 0.0 (0.0) | 16.0 (20.2) | 2.1 (2.6) | 10.4 (13.2) | 12.5 (15.8) | 9.7 (12.3) | 19.5 (24.6) | 79.3 |
| Watford | 9.3 (10.1) | 10.4 (11.2) | 1.0 (1.1) | 2.1 (2.2) | 12.4 (13.5) | 4.1 (4.5) | 18.6 (20.2) | 7.2 (7.9) | 11.4 (12.4) | 15.5 (16.9) | 92.2 |
| Waverley | 0.8 (1.6) | 1.6 (3.1) | 0.0 (0.0) | 1.6 (3.1) | 7.9 (15.6) | 5.5 (10.9) | 11.9 (23.4) | 5.5 (10.9) | 3.2 (6.3) | 12.7 (25.0) | 50.7 |
| Wealden | 6.2 (10.6) | 1.2 (2.1) | 0.6 (1.1) | 0.6 (1.1) | 12.4 (21.3) | 6.2 (10.6) | 8.1 (13.8) | 5.6 (9.6) | 7.4 (12.8) | 9.9 (17.0) | 58.2 |
| Wellingborough | 6.3 (6.6) | 7.5 (7.9) | 0.0 (0.0) | 3.8 (3.9) | 12.5 (13.2) | 10.0 (10.5) | 17.6 (18.4) | 6.3 (6.6) | 11.3 (11.8) | 20.1 (21.1) | 95.3 |
| Welwyn Hatfield | 4.9 (8.2) | 4.9 (8.2) | 0.8 (1.4) | 1.6 (2.7) | 7.3 (12.3) | 3.3 (5.5) | 10.6 (17.8) | 5.7 (9.6) | 10.6 (17.8) | 9.8 (16.4) | 59.3 |
| West Berkshire | 9.5 (11.1) | 4.4 (5.2) | 1.9 (2.2) | 2.5 (3.0) | 5.0 (5.9) | 7.6 (8.9) | 10.1 (11.9) | 12.0 (14.1) | 13.9 (16.3) | 18.3 (21.5) | 85.2 |
| West Devon | 10.8 (12.5) | 3.6 (4.2) | 9.0 (10.4) | 1.8 (2.1) | 10.8 (12.5) | 5.4 (6.3) | 9.0 (10.4) | 10.8 (12.5) | 10.8 (12.5) | 14.3 (16.7) | 86.0 |
| West Lancashire | 6.1 (8.3) | 3.5 (4.8) | 2.6 (3.6) | 1.7 (2.4) | 15.7 (21.4) | 5.2 (7.1) | 9.6 (13.1) | 9.6 (13.1) | 7.9 (10.7) | 11.4 (15.5) | 73.5 |
| West Lindsey | 6.3 (8.0) | 1.0 (1.3) | 1.0 (1.3) | 1.0 (1.3) | 18.8 (24.0) | 3.1 (4.0) | 14.6 (18.7) | 6.3 (8.0) | 6.3 (8.0) | 19.9 (25.3) | 78.4 |
| West Oxfordshire | 2.7 (4.3) | 0.9 (1.4) | 0.9 (1.4) | 2.7 (4.3) | 11.7 (18.6) | 9.9 (15.7) | 8.1 (12.9) | 7.2 (11.4) | 7.2 (11.4) | 11.7 (18.6) | 63.3 |
| West Suffolk | 7.8 (8.9) | 5.0 (5.7) | 0.6 (0.6) | 2.2 (2.5) | 14.0 (15.8) | 5.6 (6.3) | 13.4 (15.2) | 9.5 (10.8) | 7.8 (8.9) | 22.3 (25.3) | 88.2 |
| Westminster | 19.9 (18.4) | 9.2 (8.5) | 2.7 (2.5) | 0.4 (0.4) | 5.4 (5.0) | 4.2 (3.9) | 24.1 (22.3) | 16.1 (14.9) | 10.7 (9.9) | 15.3 (14.2) | 107.9 |
| Wigan | 6.1 (5.5) | 4.9 (4.4) | 0.6 (0.5) | 2.7 (2.5) | 21.3 (19.1) | 4.6 (4.1) | 22.5 (20.2) | 8.2 (7.4) | 15.2 (13.7) | 25.3 (22.7) | 111.4 |
| Wiltshire | 6.0 (8.8) | 2.2 (3.2) | 0.4 (0.6) | 3.6 (5.3) | 9.8 (14.4) | 5.6 (8.2) | 10.0 (14.7) | 9.0 (13.2) | 6.6 (9.7) | 14.8 (21.8) | 68.0 |
| Winchester City | 7.2 (12.3) | 4.0 (6.8) | 0.0 (0.0) | 0.0 (0.0) | 8.0 (13.7) | 3.2 (5.5) | 8.8 (15.1) | 6.4 (11.0) | 8.8 (15.1) | 12.0 (20.5) | 58.5 |
| Windsor and Maidenhead | 5.9 (8.5) | 4.6 (6.6) | 1.3 (1.9) | 0.0 (0.0) | 11.2 (16.0) | 5.9 (8.5) | 13.9 (19.8) | 7.9 (11.3) | 9.2 (13.2) | 9.9 (14.2) | 70.0 |
| Wirral | 7.4 (7.2) | 3.1 (3.0) | 2.5 (2.4) | 1.2 (1.2) | 18.2 (17.8) | 4.0 (3.9) | 19.4 (19.0) | 15.7 (15.4) | 8.6 (8.4) | 22.2 (21.7) | 102.5 |
| Woking | 5.0 (5.4) | 9.9 (10.9) | 2.0 (2.2) | 2.0 (2.2) | 12.9 (14.1) | 7.9 (8.7) | 13.9 (15.2) | 9.9 (10.9) | 10.9 (12.0) | 16.9 (18.5) | 91.3 |
| Wokingham | 1.2 (3.2) | 0.0 (0.0) | 0.6 (1.6) | 0.0 (0.0) | 2.9 (8.1) | 5.3 (14.5) | 8.8 (24.2) | 3.5 (9.7) | 2.9 (8.1) | 11.1 (30.6) | 36.2 |
| Wolverhampton | 6.8 (5.9) | 4.9 (4.3) | 4.2 (3.6) | 1.1 (1.0) | 28.5 (24.8) | 4.6 (4.0) | 14.4 (12.5) | 16.7 (14.5) | 14.0 (12.2) | 19.7 (17.2) | 115.1 |
| Worcester City | 7.9 (7.1) | 7.9 (7.1) | 0.0 (0.0) | 1.0 (0.9) | 18.8 (16.8) | 5.9 (5.3) | 18.8 (16.8) | 20.7 (18.6) | 10.9 (9.7) | 19.8 (17.7) | 111.6 |
| Worthing | 8.1 (7.7) | 11.8 (11.1) | 1.8 (1.7) | 0.9 (0.9) | 18.1 (17.1) | 5.4 (5.1) | 12.7 (12.0) | 9.0 (8.5) | 12.7 (12.0) | 25.3 (23.9) | 105.8 |
| Wychavon | 6.2 (9.1) | 3.1 (4.5) | 0.0 (0.0) | 0.8 (1.1) | 14.7 (21.6) | 3.9 (5.7) | 10.0 (14.8) | 9.3 (13.6) | 5.4 (8.0) | 14.7 (21.6) | 68.0 |
| Wycombe | 4.6 (6.2) | 7.4 (10.2) | 1.1 (1.6) | 0.6 (0.8) | 6.9 (9.4) | 5.7 (7.8) | 16.0 (21.9) | 9.7 (13.3) | 10.3 (14.1) | 10.9 (14.8) | 73.3 |
| Wyre | 8.9 (7.0) | 2.7 (2.1) | 6.2 (4.9) | 2.7 (2.1) | 26.8 (21.0) | 3.6 (2.8) | 21.4 (16.8) | 19.6 (15.4) | 10.7 (8.4) | 25.0 (19.6) | 127.6 |
| Wyre Forest | 2.0 (2.1) | 3.0 (3.1) | 1.0 (1.0) | 3.0 (3.1) | 20.7 (21.6) | 2.0 (2.1) | 15.8 (16.5) | 11.8 (12.4) | 11.8 (12.4) | 24.7 (25.8) | 95.8 |
|  | **Outlets (n (%)) per 100,000 population by cuisine type** | | | | | | | | | | |
| **Local Authority** | **Burger** | **Chicken** | **Desserts** | **Fast food** | **Fish & Chips** | **Kebab** | **Pizza** | **Sand/Caf/Bake** | **S Asian** | **SE & E Asian** | **Overall** |
| York | 4.7 (4.6) | 2.4 (2.3) | 1.9 (1.8) | 0.9 (0.9) | 15.2 (14.7) | 3.8 (3.7) | 22.3 (21.7) | 18.0 (17.5) | 12.3 (12.0) | 21.4 (20.7) | 103.0 |
| England | 7.7 (8.0) | 6.8 (7.1) | 1.8 (1.9) | 1.8 (1.9) | 14.8 (15.4) | 5.9 (6.1) | 15.5 (16.1) | 12.2 (12.7) | 11.5 (12.7) | 18.2 (18.9) | 96.4 |


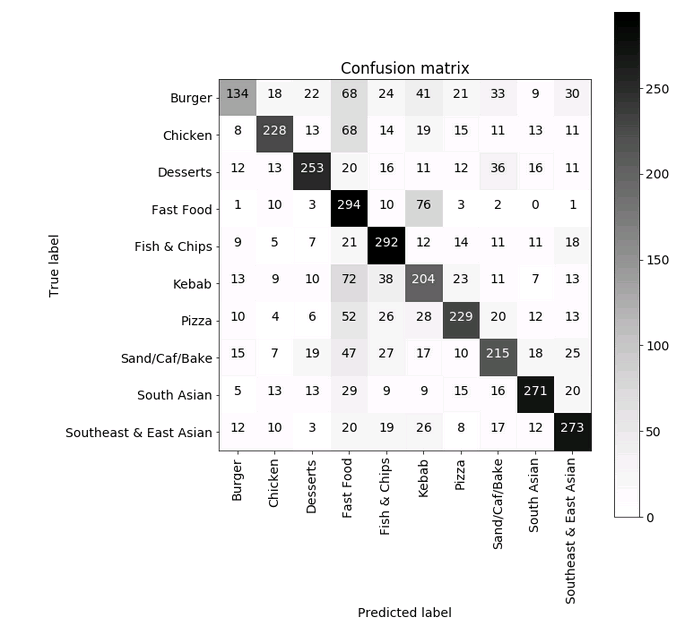


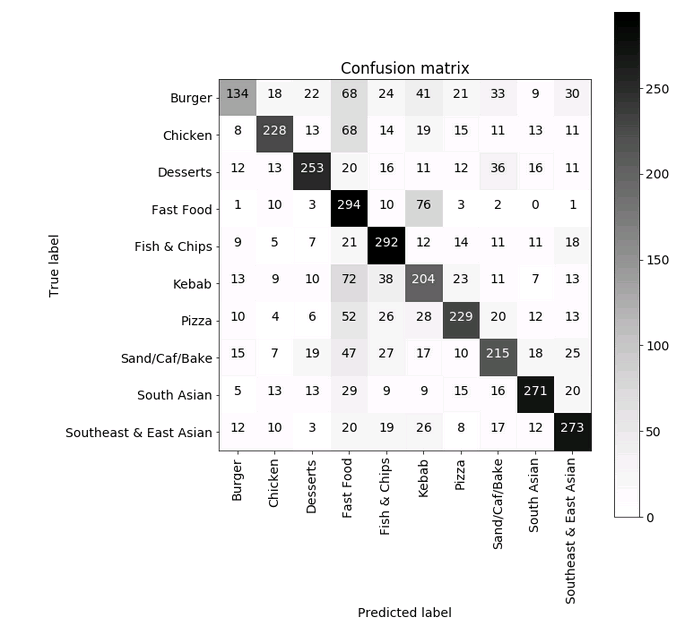


250

200

150

100

50

0

**Actual cuisine type**

**Predicted cuisine type**

Fig A1: Confusion matrix, showing specific instances of misclassification, according to the naïve classifier. Rows total to 400 outlets.


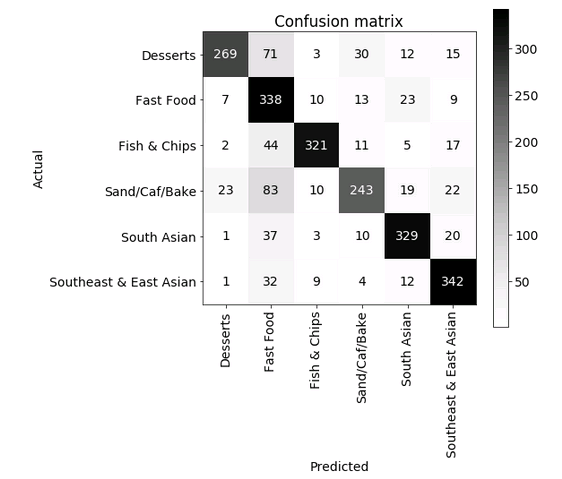

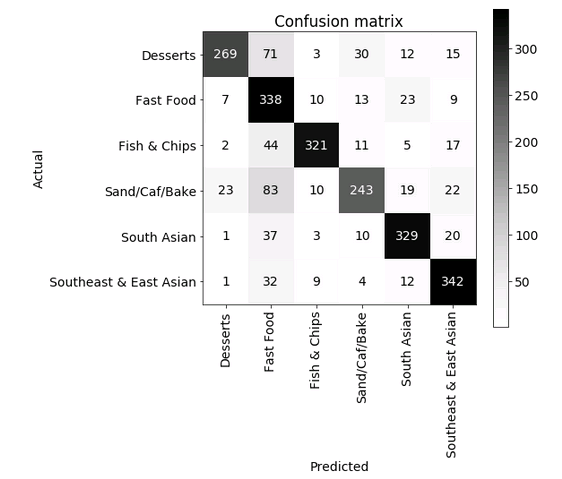


**Actual cuisine type**

**Predicted cuisine type**

Fig A2: Confusion matrix, showing specific instances of misclassification, according to the six-point classifier. Rows total to 400 outlets.


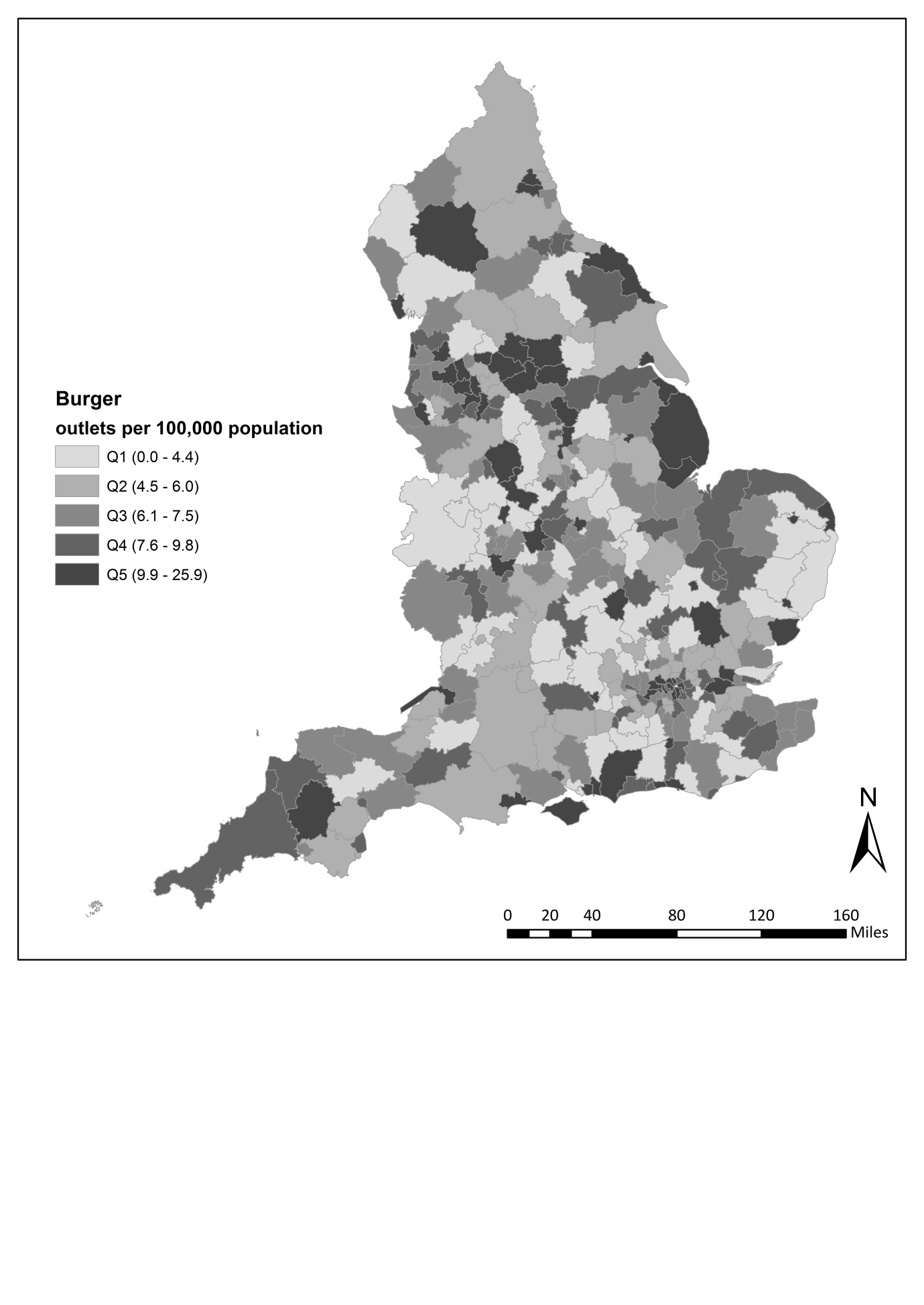


Fig A3: Number of burger outlets per local authority per 100,000 population (quintiles (Q)). Excludes the City of London as an extreme outlier, with 318.9 outlets / 100,000 population.

Source: Office for National Statistics licensed under the Open Government Licence v.3.0. Contains OS data © Crown copyright and database right 2020.


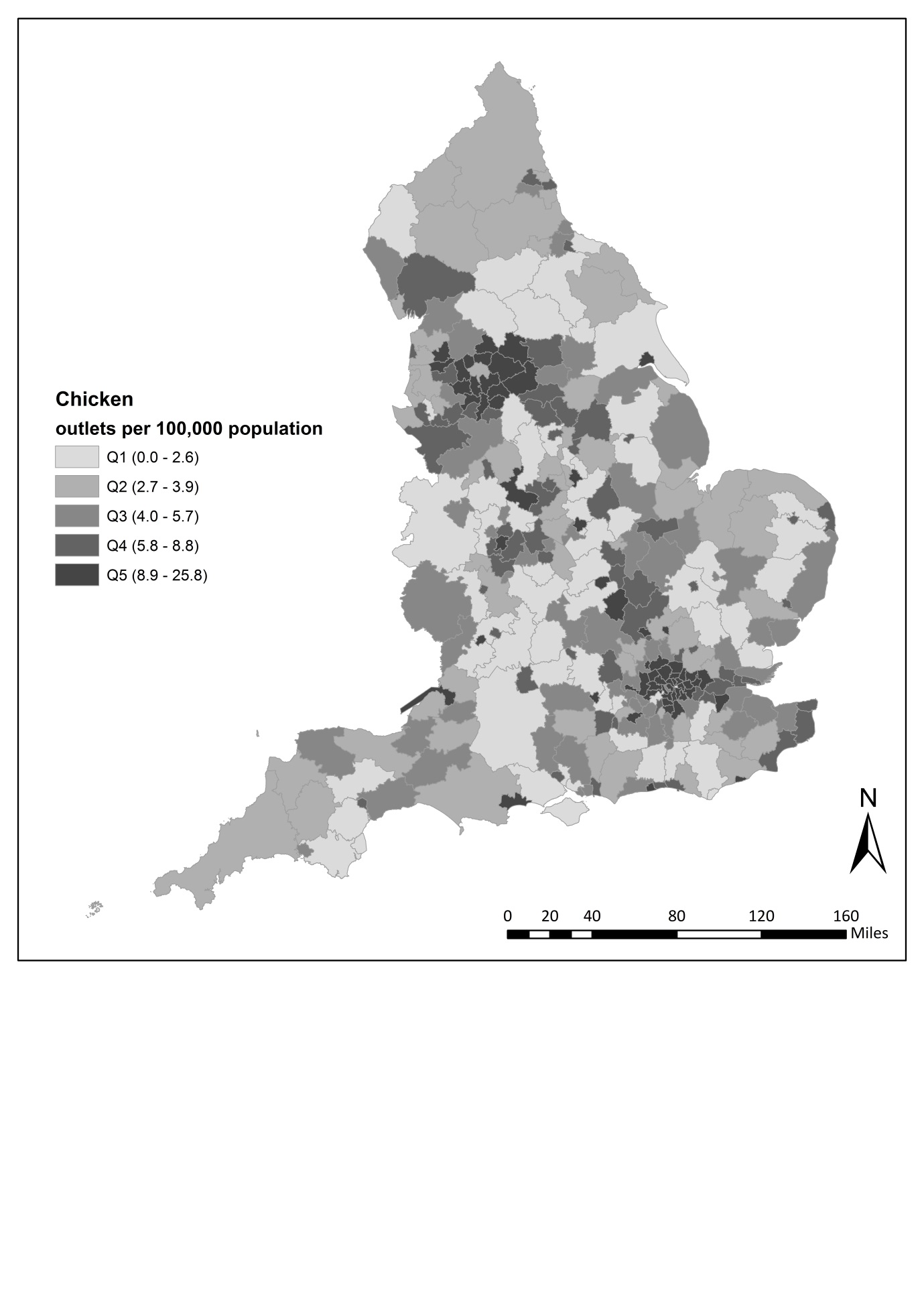


Fig A4: Number of chicken outlets per local authority per 100,000 population (quintiles (Q)). Excludes the City of London as an extreme outlier, with 113.2 outlets / 100,000 population.

Source: Office for National Statistics licensed under the Open Government Licence v.3.0. Contains OS data © Crown copyright and database right 2020.


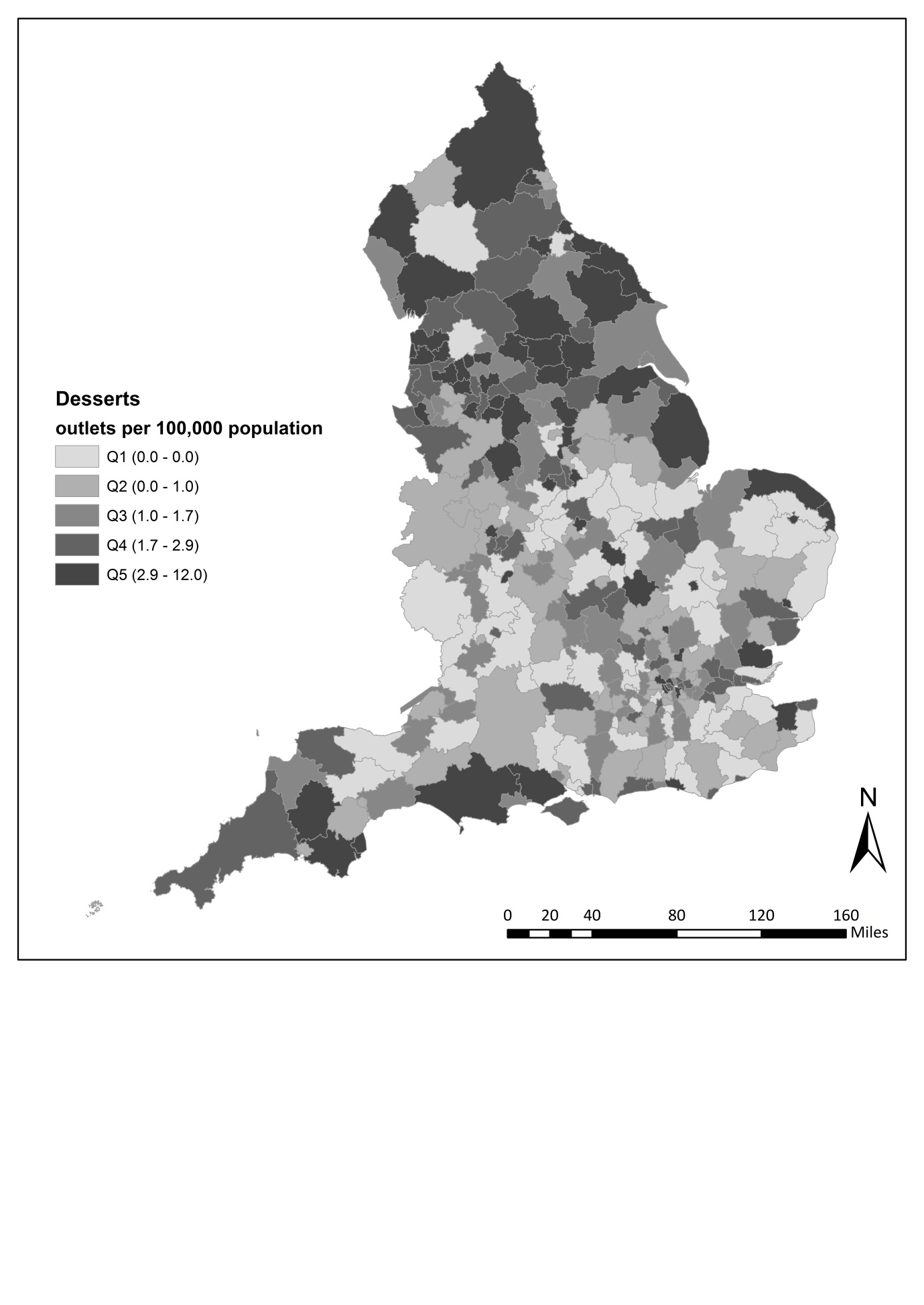


Fig A5: Number of dessert outlets per local authority per 100,000 population (quintiles (Q)). Excludes the City of London as an extreme outlier, with 41.1 outlets / 100,000 population.

Source: Office for National Statistics licensed under the Open Government Licence v.3.0. Contains OS data © Crown copyright and database right 2020.


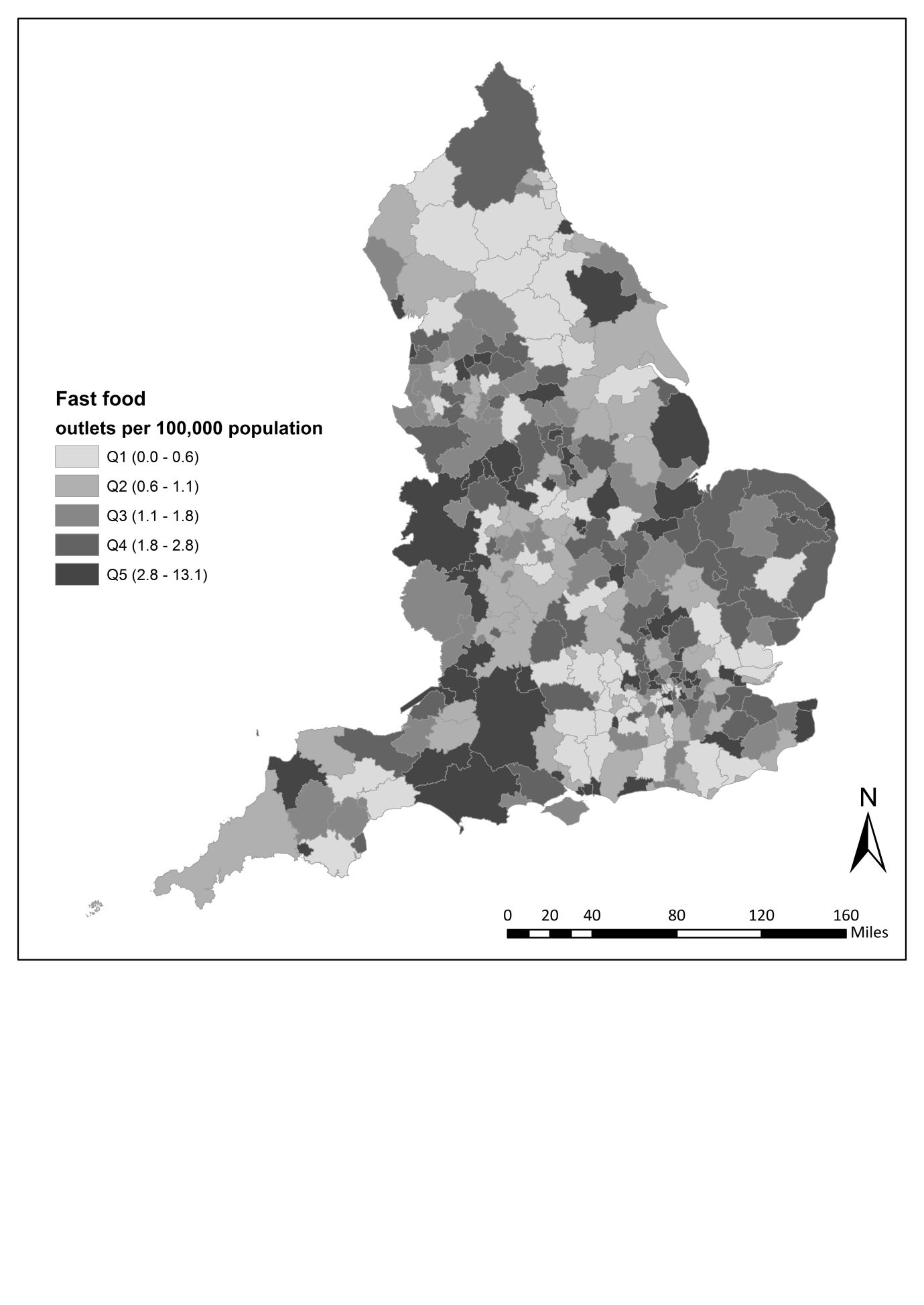


Fig A6: Number of multi fast-food outlets per local authority per 100,000 population (quintiles (Q)).

Source: Office for National Statistics licensed under the Open Government Licence v.3.0. Contains OS data © Crown copyright and database right 2020.


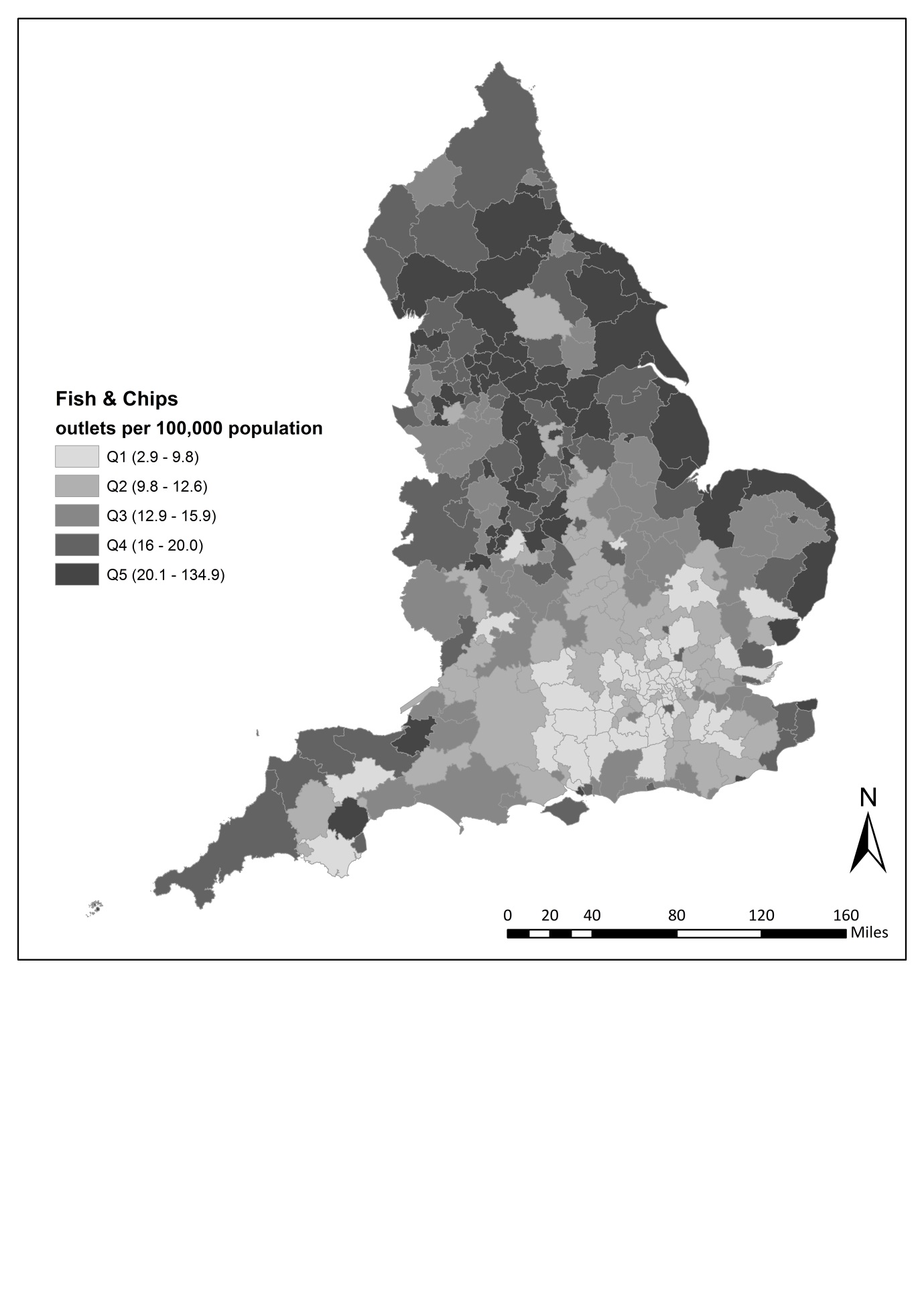


Fig A7: Number of fish & chip shops per local authority per 100,000 population (quintiles (Q)). Excludes the City of London as an extreme outlier, with 298.3 outlets / 100,000 population.

Source: Office for National Statistics licensed under the Open Government Licence v.3.0. Contains OS data © Crown copyright and database right 2020.


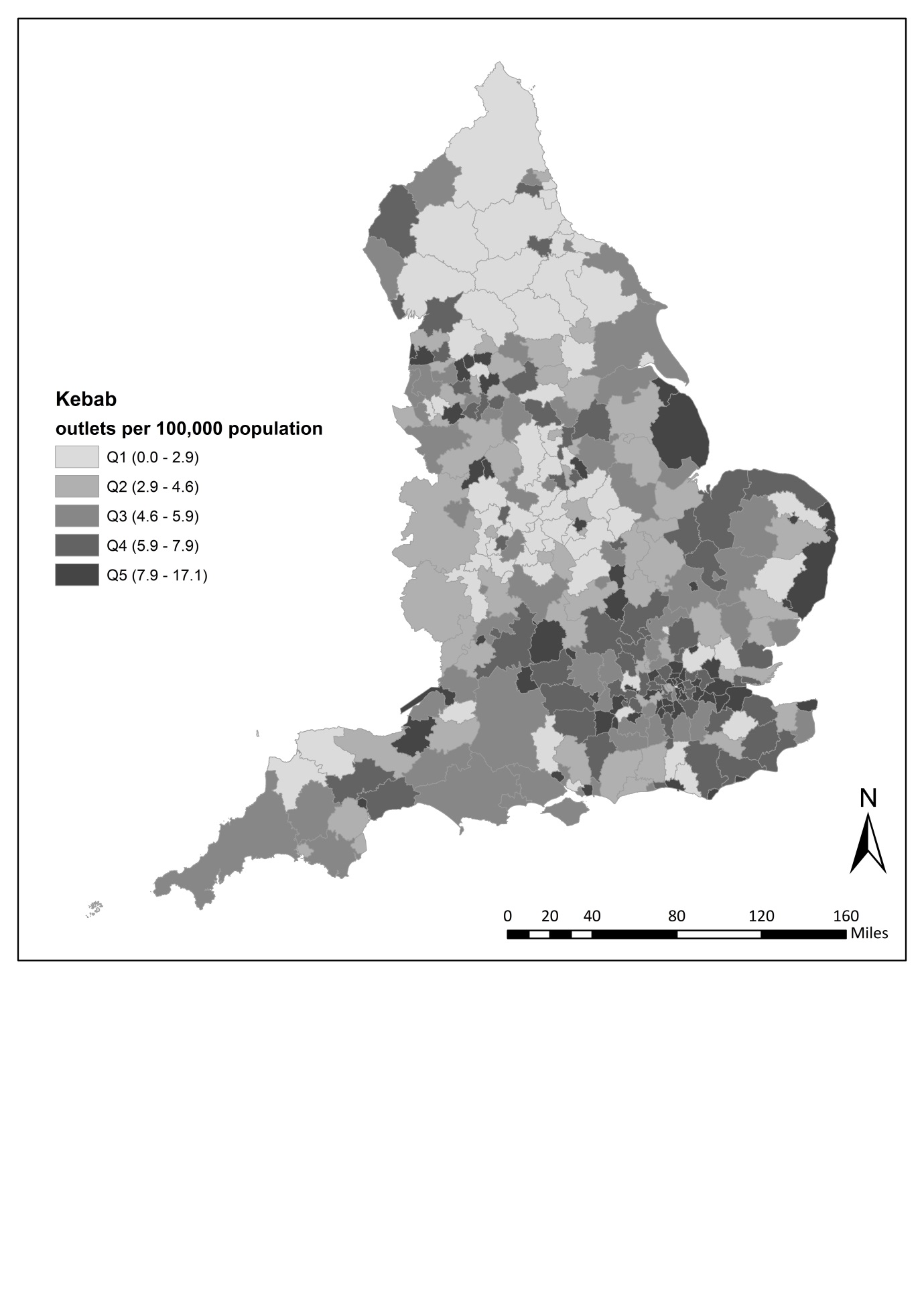


Fig A8: Number of kebab shops per local authority per 100,000 population (quintiles (Q)). Excludes the City of London as an extreme outlier, with 185.2 outlets / 100,000 population.

Source: Office for National Statistics licensed under the Open Government Licence v.3.0. Contains OS data © Crown copyright and database right 2020.


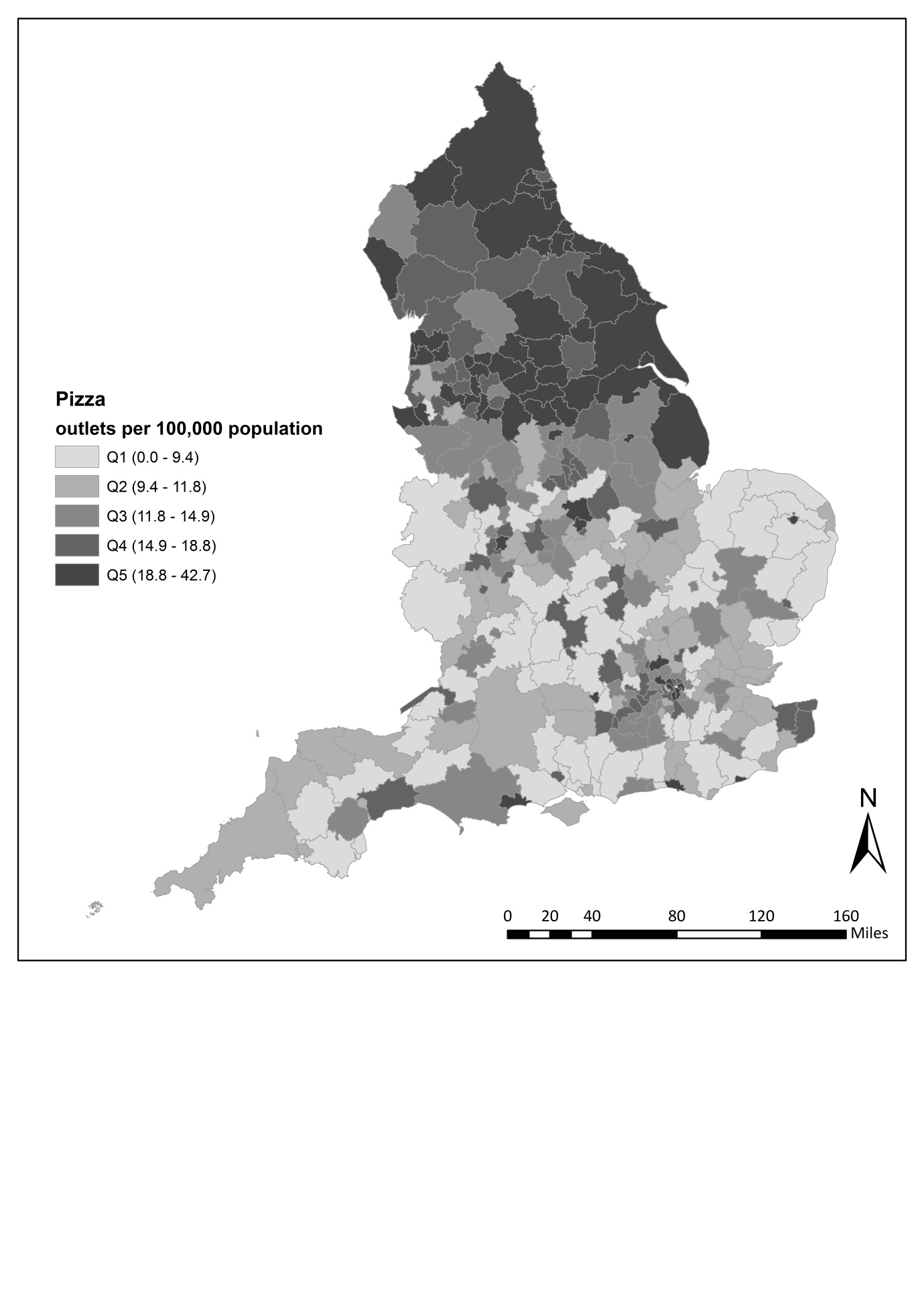


Fig A9: Number of pizza outlets per local authority per 100,000 population (quintiles (Q)). Excludes the City of London as an extreme outlier, with 421.8 outlets / 100,000 population.

Source: Office for National Statistics licensed under the Open Government Licence v.3.0. Contains OS data © Crown copyright and database right 2020.


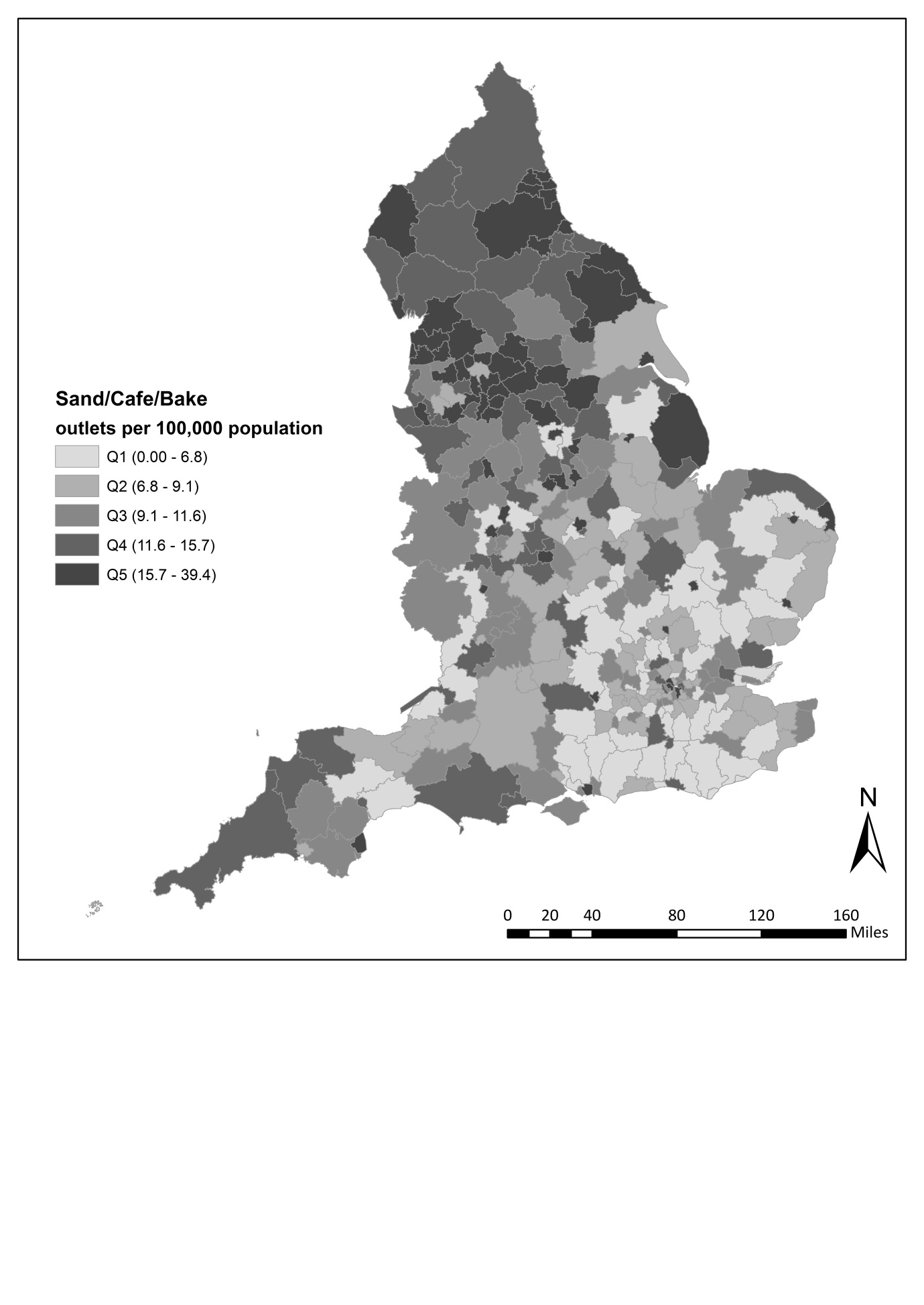


Fig A10: Number of sandwich shops/cafés/bakeries per local authority per 100,000 population (quintiles (Q)). Excludes the City of London as an extreme outlier, with 1172.7 outlets / 100,000 population.

Source: Office for National Statistics licensed under the Open Government Licence v.3.0. Contains OS data © Crown copyright and database right 2020.


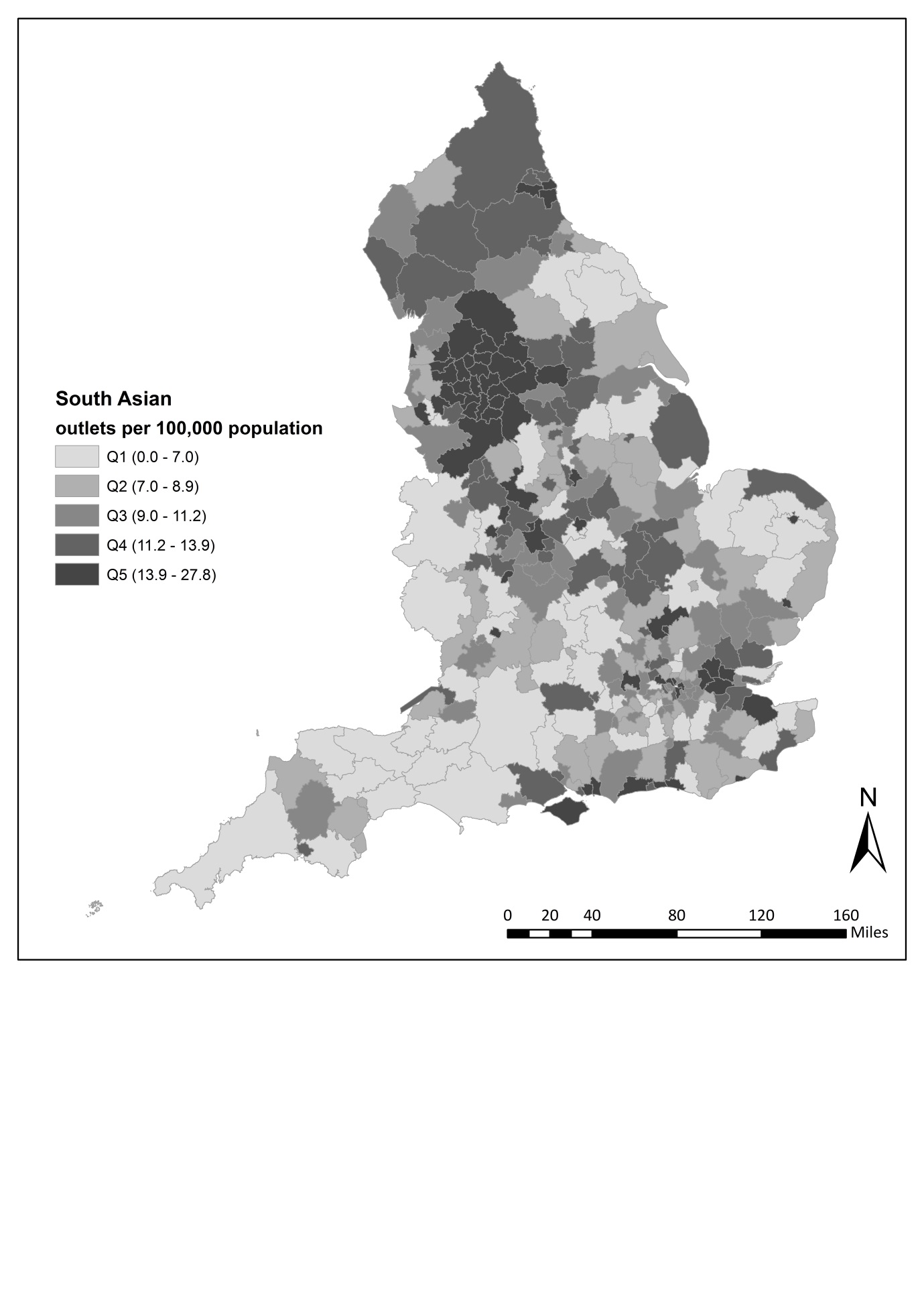


Fig A11: Number of South Asian outlets per local authority per 100,000 population (quintiles (Q)). Excludes the City of London as an extreme outlier, with 761.2 outlets / 100,000 population.

Source: Office for National Statistics licensed under the Open Government Licence v.3.0. Contains OS data © Crown copyright and database right 2020.


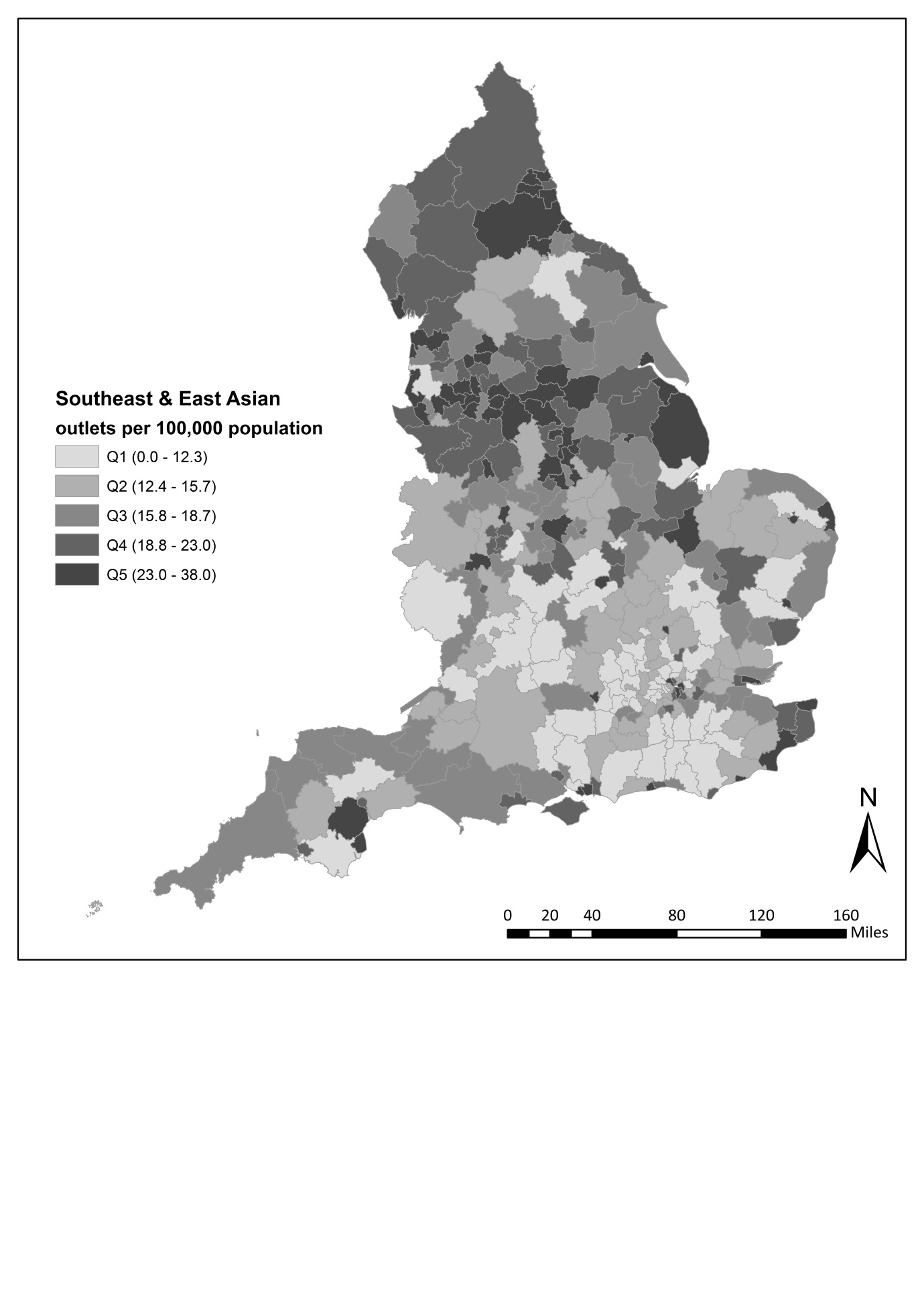


Fig A12: Number of Southeast & East Asian outlets per local authority per 100,000 population (quintiles (Q)). Excludes the City of London as an extreme outlier, with 586.4 outlets / 100,000 population.

Source: Office for National Statistics licensed under the Open Government Licence v.3.0. Contains OS data © Crown copyright and database right 2020.
